# diPaRIS: Dynamic and Interpretable Protein‐RNA Interactions Prediction With U‐Shaped Network and Novel Structure Encoding

**DOI:** 10.1002/advs.202506314

**Published:** 2025-08-29

**Authors:** Lishen Zhang, Chengqian Lu, Xiaoqing Peng, Fei Guo, Hongdong Li, Jianxin Wang

**Affiliations:** ^1^ School of Computer Science and Engineering Central South University Changsha 410083 China; ^2^ Xiangjiang Laboratory Changsha 410205 China; ^3^ Hunan Provincial Key Lab on Bioinformatics Central South University Changsha 410083 China; ^4^ School of Computer Science Xiangtan University Xiangtan 411105 China; ^5^ Center for Medical Genetics & Hunan Key Laboratory of Medical Genetics School of Life Sciences Central South University Changsha 410083 China; ^6^ Xinjiang Engineering Research Center of Big Data and Intelligent Software School of Software Xinjiang University Urumqi 830091 China

**Keywords:** deep learning, interpretable analysis, Protein‐RNA interactions, RNA structure encoding

## Abstract

Protein‐RNA interactions play a critical role in various biological processes and disease development. Proteins interact with RNAs through dynamic binding sites that exhibit specific structural patterns under various cellular conditions. While current computational methods take RNA structures in vivo into account, they fall short in capturing the structure contextual association of nucleotides, limiting predictive accuracy. Here, diPaRIS, a deep learning method, is proposed to predict dynamic protein‐RNA interactions with improved accuracy and enhanced interpretability by integrating RNA structures in vivo. diPaRIS introduces a novel encoding scheme for SHAPE‐seq to encode nucleotide correlations, providing a more comprehensive representation of RNA structures. Leveraging a U‐shaped network architecture, diPaRIS not only improves prediction performance but also enables interpretable analysis by learning sequence binding motifs and generating attribution maps. Benchmarking diPaRIS across 44 datasets shows its superiority over existing methods. The model consistently achieves the highest accuracy, AUC, AUPR, and F1‐score across all datasets. Additionally, diPaRIS excels in cross‐cell line predictions, consistently outperforming the second‐best method across all datasets. Predictions by diPaRIS reflect the conservation of protein‐RNA binding and facilitate further functional interpretation of genetic variants in complex diseases. The findings highlight that diPaRIS effectively predicts protein‐RNA interactions and interprets potential gene‐disease associations.

## Graphical abstract

This study presents diPaRIS, a deep learning method designed to predict dynamic protein‐ RNA interactions with enhanced accuracy. By integrating RNA structures in vivo through a novel encoding scheme for nucleotide correlations, diPaRIS improves the interpretability of binding motifs and outperforms current state‐of‐the‐art methods across multiple datasets. The results provide new insights into protein‐RNA binding preferences and their role in disease development.

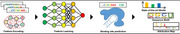

## 1 Introduction

RNA‐binding proteins (RBPs) are a group of proteins that interact with RNAs through binding sites,^[^ ^1^ ^]^ playing essential roles in transcription, translation, and other biological processes.^[^ ^2^ ^]^ For instance, DEAD/ H‐box RNA helicases (DDXs) reconfigure complex RNA structures like hairpins and mRNP complexes, thereby influencing mRNA synthesis and processing.^[^ ^3^ ^]^ Insulin‐like growth factor 2 mRNA‐binding proteins (IGF2BPs) bind to methylated mRNAs, orchestrating storage, stability, and translation.^[^ ^4^ ^]^ Dysregulation of these protein‐RNA interactions, driven by genetic variants, contributes to the development of many diseases, particularly malignant tumors.^[^ ^5^ ^]^ Dynamic protein‐RNA interactions, which involve variations in binding sites driven by factors like selective gene expression and alternative splicing across different cellular environments in vivo, are crucial for regulating gene expression and cellular processes. Accurately predicting these context‐dependent RNA‐protein interactions is essential for unraveling regulatory mechanisms and understanding their biological implications in disease development. High‐throughput sequencing techniques, such as RNA immunoprecipitation sequencing (RIP‐seq)^[^ ^6^ ^]^ and cross‐linking immunoprecipitation sequencing (CLIP‐seq),^[^ ^7^ ^]^ provide precise maps of RBP binding sites in vivo, offering gold‐standard datasets for developing computational models. However, these experimental methods are time‐consuming, labor‐intensive, and expensive. Furthermore, due to technical limitations, many RBP binding sites remain undetected.^[^ ^8^ ^]^ Therefore, efficient and accurate computational models to predict RBP binding sites are becoming increasingly important.^[^ ^9^ ^]^

Computational methods have been developed to predict RBP binding sites utilizing RNA sequence and structure information.^[^ ^10^ ^]^ Sequence‐based approaches, such as DeepBind,^[^ ^11^ ^]^ introduced by Alipanahi et al., employs convolutional neural networks (CNNs) to model proximal sequence dependencies and identify binding motifs from CLIP‐seq data. By generating sequence motifs, DeepBind not only determines how genetic variants affect binding sites but also helps identify core regulatory factors associated with specific diseases. DeepRiPe,^[^ ^12^ ^]^ a sequence‐based method, combines CNNs with Recurrent Neural Networks (RNNs) to capture distant sequential dependencies and enhance predictive performance. DeepRiPe identifies sequence motifs within broader transcript context patterns, aiding in the understanding of RBP regulatory mechanisms. However, sequence‐based methods fail to account for RNA structural information, which is crucial for regulating protein‐RNA interactions under diverse cellular conditions.^[^ ^13^ ^]^

This weakness has prompted the development of structure‐based approaches. Sun et al. developed PrismNet,^[^ ^14^ ^]^ a model that integrates RNA structural data derived from in vivo click selective 2'‐hydroxyl acylation and profiling experiments (icSHAPEs)^[^ ^15^ ^]^ sequencing. By employing residual CNNs networks augmented with a squeeze‐and‐excitation (SE) module, PrismNet captures global information more effectively, resulting in improved prediction. Zhu et al. proposed HDRNet,^[^ ^16^ ^]^ which combines *k‐mer* embedding sequences with structures from icSHAPE‐seq data. HDRNet uses multi‐scale residual networks to capture contextual dependencies between nucleotides. Although HDRNet improved prediction accuracy, its encoding strategy limits resolution at the single‐nucleotide level, reducing its ability to interpret fine‐grained sequence features. Despite the advancements of these structure‐based models, they encode the structure of each nucleotide individually, failing to capture the dynamic interdependence between adjacent nucleotides that defines RNA folding and function.^[^ ^17^ ^]^ Due to this oversight, the models are unable to predict protein‐RNA interactions accurately in diverse biological contexts.^[^ ^18^ ^]^

To address this challenge, we propose a novel deep learning method that integrates RNA structures in vivo to predict **d** ynamic and **i** nterpretable **p** rotein‐ **R** NA **i** nteraction **s** (**diPaRIS**) with improved accuracy and enhanced interpretability. To effectively capture these dynamic biological realities and improve prediction accuracy, diPaRIS employs a novel encoding scheme that represents dynamic RNA structures and leverages robust feature integration to model the complexities of protein‐RNA binding under diverse conditions. diPaRIS employs a U‐shaped deep residual network for feature extraction, using a contraction path to locate core binding site elements and a symmetric expansion path to enhance binding site features. Attention mechanisms are incorporated to capture both proximal and distant nucleotide dependencies, enhancing feature relevance. Finally, a pyramid pooling module with global expectation pooling^[^ ^19^ ^]^ further refines predictions by aggregating multi‐scale features to boost accuracy.

Benchmarking results on 44 datasets involving different proteins and cellular conditions demonstrate that diPaRIS outperforms state‐of‐the‐art methods on six performance metrics, including accuracy (ACC), area under the receiver operating characteristic curve (AUC), area under the precision‐recall curve (AUPR), precision, F1‐score, and recall. diPaRIS achieves the highest ACC, AUC, AUPR, and F1‐score across all datasets. Furthermore, when tested on datasets involving the same proteins across different cell lines, diPaRIS surpasses the second‐best method in every dataset.

diPaRIS also enables interpretable analysis by learning sequence binding motifs and generating attribution maps from the model. When analyzing datasets from different cell lines and proteins, diPaRIS effectively captures motifs that reflect the conservation of protein‐RNA binding. By extracting attribution maps, diPaRIS provides insights into molecular interactions and functional mechanism changes caused by genetic variants in complex diseases. Our analysis highlights that diPaRIS not only effectively predicts RBP binding sites but also provides insights into the potential functional influence of genetic variants on protein‐RNA interactions and disease progression.

## 2 Results

### 2.1 Overview of diPaRIS

**Figure 1:**
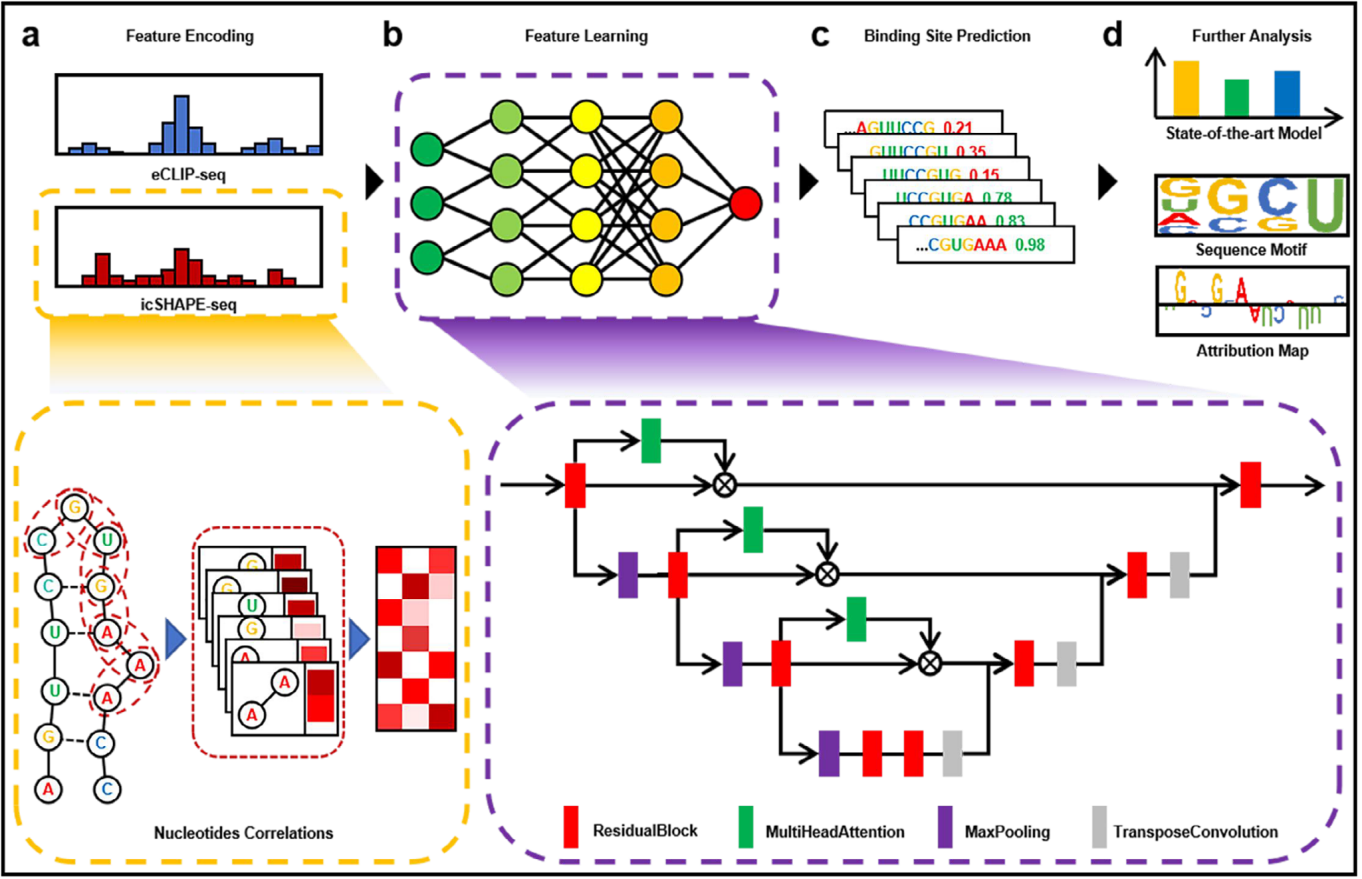
diPaRIS architecture. a) Feature encoding: encoding sequence features (derived from eCLIP‐seq) and structural features (obtained from icSHAPE‐seq). b) Feature learning: learning concatenated sequence and structure features through a U‐shaped network integrated with multi‐head attention mechanisms. c) Binding site prediction: diPaRIS outputs a predicted score for each binding site. d) Further analysis: utilizing the predictions from diPaRIS for downstream analysis.

diPaRIS is a method for predicting protein binding sites, providing precise and robust predictions by leveraging encoded sequences (derived from enhanced CLIP‐seq, eCLIP‐seq) and structural features (obtained from icSHAPE‐seq). Its key contribution is the development of the icSHAPE‐ **D** ynamic **S** tructure (**icSHAPE‐DS**) encoding scheme, designed to capture the dynamic RNA structures by accounting for structural correlations between adjacent nucleotides (**Figure** **1**a, Experimental Section).

The method consists of three modules: feature encoding, feature learning, and binding site prediction. First, sequence and structural features are extracted from sequencing data. These features are subsequently processed and aligned through a self‐attention mechanism consisting of CNNs and a bidirectional Long Short‐Term Memory (Bi‐LSTM) network. Next, sequence and structural features are learned through a U‐shaped network integrating multi‐head attention mechanisms to extract meaningful patterns (Figure 1b, Experimental Section). Finally, after aggregating features from different regional scales using pyramid pooling and global expectation pooling,^[^ ^19^ ^]^ diPaRIS outputs a predicted score for each binding site using a multilayer perceptron (MLP) (Figure 1c). These predictions are then used for downstream analysis of binding sites (Figure 1d).

### 2.2 Data Processing and Performance Evaluation

In diPaRIS, eCLIP‐seq is used to generate sequence features of protein binding sites, while icSHAPE‐seq is employed to characterize their structural features. To ensure consistency and mitigate batch effects, eCLIP‐seq data were collected from the same batch in the ENCODE database.^[^ ^20^ ^]^ The binding sites were required to meet stringent enrichment criteria relative to input controls and to be reproducibly identified across biological replicates in HepG2 (human hepatocellular carcinoma cells) or K562 (human chronic myeloid leukemia cells) cell lines.^[^ ^8^ ^]^ Structural data were obtained from icSHAPE‐seq experiments in Zhang's lab,^[^ ^14^ ^]^ which provided in vivo structural context for these binding sites. In total, we collected 223 eCLIP‐seq datasets, which encompass information on 844,854 binding sites, along with two corresponding icSHAPE‐seq datasets for these cell lines. All sequencing data were quality‐controlled and processed using the human genome GRCh37/hg19 as a reference.^[^ ^21^ ^]^ Each analyzed binding site is covered by both sequencing data to provide comprehensive sequence and structural information. After rigorous filtering, a total of 302,146 binding sites remained for further analysis. Following protocols from previous studies,^[^ ^11^, ^14^, ^16^ ^]^ binding sites were standardized to 101 nucleotides in length to generate uniform positive samples. To fully capture the complexity of true negative interactions as much as possible, negative samples were randomly generated from non‐binding regions in equal numbers to positive samples, processed via the same pipeline. To ensure sufficient samples for model training, we retained only datasets with >2000 samples. In total, 44 datasets were constructed, comprising 464,112 samples. Each dataset was labeled as “flag protein ‐ background cell line” (e.g., IGF2BP1‐K562), covering 34 RBPs in HepG2 or K562 cell lines (Table S1). Potential biases inherent in public datasets reflect those noted in existing literature, and the generation of negative samples from non‐binding regions represents an approximation that may not fully capture the complexity of true negative interactions. However, we mitigated these biases by extensively testing across 44 diverse datasets.

For each dataset, a predictive model was trained using 80% of the samples and the remaining 20% was used for independent testing. Model performance was assessed through fivefold cross‐validation. The training model learns parameters that minimize cross‐entropy loss. diPaRIS and other state‐of‐the‐art models were trained under the same criteria, and their performance was compared using the following metrics: ACC, AUC, AUPR, F1‐score, precision, and recall. These metrics collectively provide a comprehensive evaluation of each model's predictive capability.

### 2.3 diPaRIS Outperforms State‐Of‐the‐Art Methods

**Figure 2:**
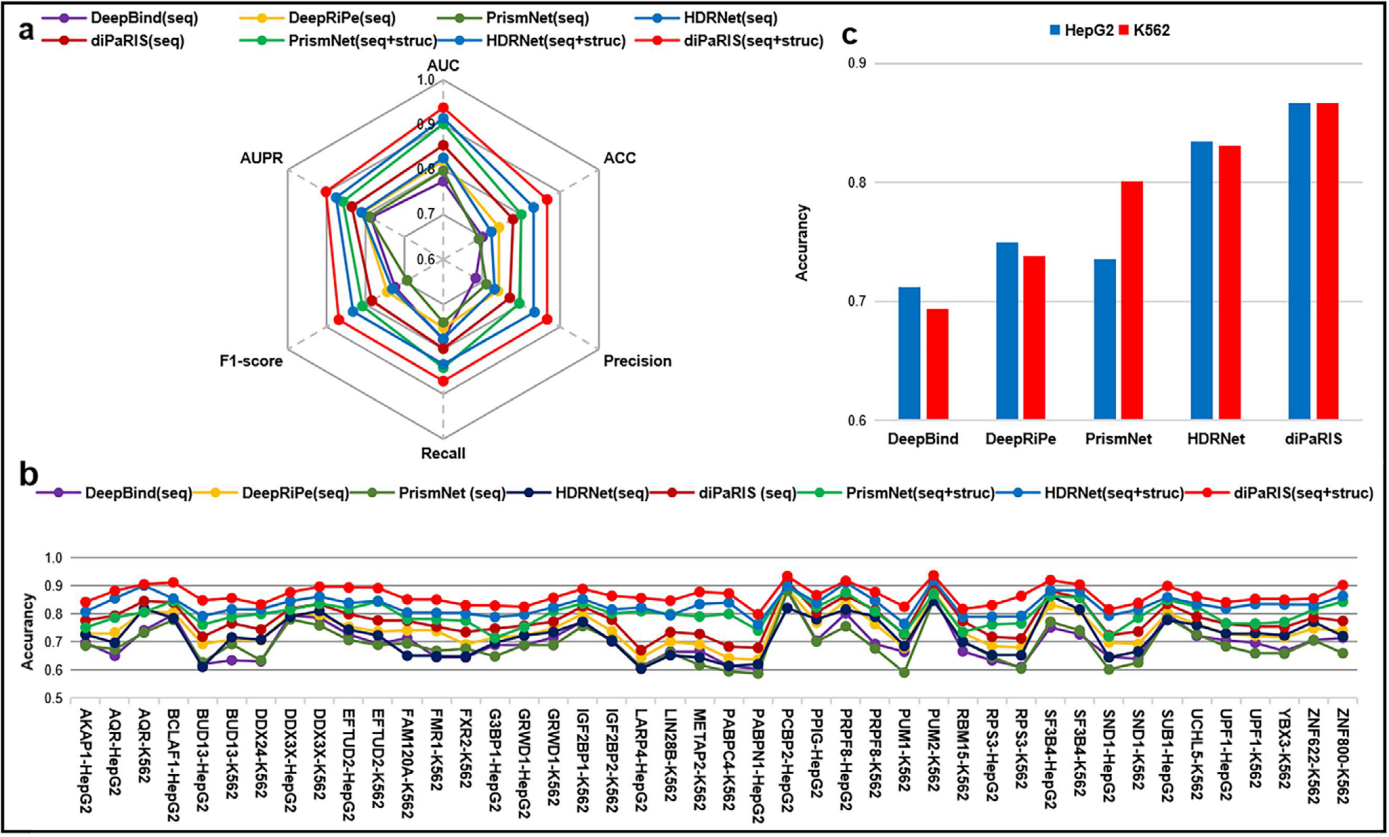
Comparison of diPaRIS performance with state‐of‐the‐art methods. a) The average performance of diPaRIS and other comparative models for all datasets based on AUC, AUPR, ACC, F1‐score, precision, and recall. b) ACC scores of different methods on each dataset. In (a) and (b), (seq) means the model uses only sequence features as input, while (seq+struc) means it uses both sequence and structure features. c) Average ACC scores across all datasets for HepG2 and K562 cell lines.

To evaluate the predictive performance of diPaRIS, we compared it with four state‐of‐the‐art methods: DeepBind,^[^ ^11^ ^]^ DeepRiPe,^[^ ^12^ ^]^ PrismNet,^[^ ^14^ ^]^ and HDRNet.^[^ ^16^ ^]^ Comparative methods are trained on each of the 44 datasets and subsequently evaluated on their respective test datasets. As shown in **Figure** **2**a, diPaRIS achieves superior performance compared to the comparative methods across all datasets. Our model outperforms state‐of‐the‐art methods across all six metrics, notably achieving an average AUC of 0.9381 and an AUPR of 0.9017, both exceeding 0.9. To determine the significance of the performance differences between diPaRIS and the competing methods, a statistical test was conducted. The t‐test results indicate that diPaRIS significantly improves predictive performance across all metrics (p‐value < 0.05, Table S2, Supporting Information).

To provide a more detailed view, the ACC performance for each dataset is presented in Figure 2b (the performances of other metrics are provided in Figure S1 and Tables S3 to S8, Supporting Information). diPaRIS achieves the highest ACC scores on all datasets. It performs particularly well on the PUM2‐K562, PCBP2‐HepG2, and SF3B4‐HepG2 datasets, with ACC values of 0.9384, 0.9355, and 0.9209, respectively. Moreover, diPaRIS demonstrates significant improvements in ACC for the RPS3‐K562, PUM1‐K562, and BCLAF1‐HepG2 datasets, with increases of 8.20%, 7.31%, and 6.63%, respectively, compared to the second‐best methods. In addition, diPaRIS also excels in AUC, AUPR, and F1‐score across all datasets, outperforming competing methods. Baseline models sometimes showed higher Precision or Recall individually, but this improvement often came at the expense of the other metric. In contrast, diPaRIS delivers balanced performance, consistently attaining top rankings in accuracy and F1 scores.

We compared diPaRIS to other methods to assess its robustness and performance across different cell conditions. The average ACC of all datasets in the context of HepG2 or K562 cell lines is displayed in Figure 2c. diPaRIS demonstrates consistent and superior predictive power across the two cell lines, with an average ACC of 0.8669 for HepG2 and 0.8666 for K562. These results surpass those of the second‐best method, HDRNet, which achieved an ACC of 0.8342 for HepG2 and 0.8308 for K562.

### 2.4 Structural Information Effectively Improves the Model's Performance

**Figure 3:**
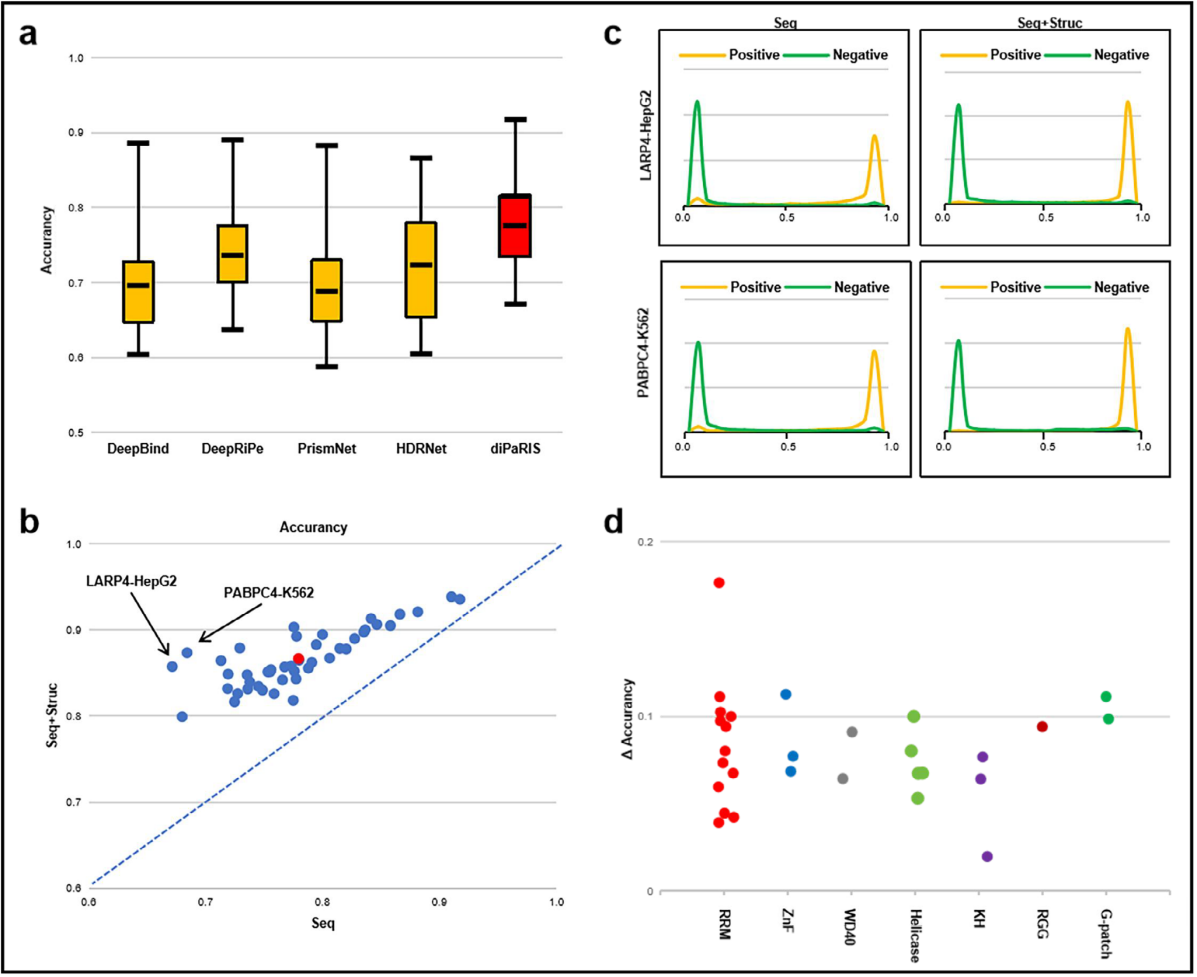
Structural information enhances model performance. a) ACC performance across all datasets. Each method takes only the one‐hot encoding sequence feature as input. The center line represents the median; box bounds indicate the upper and lower quartiles. b) The improvement in ACC performance for each dataset as predicted by diPaRIS, with and without structural features. The vertical coordinates represent the performance when using both sequence and structural features (Seq+Struc), while the horizontal coordinates represent the performance when using sequence features only (Seq). Red dots in the figures indicate the average ACC differences across all datasets. c) The distribution of predicted scores from diPaRIS with and without structural features for the LARP4‐HepG2 and PABPC4‐K562 datasets. d) The difference in ACC obtained by diPaRIS with and without structural features across various domains.

For the comparative methods, DeepBind and DeepRiPe rely solely on sequence‐based information, while PrismNet, HDRNet, and diPaRIS integrate both sequence and structural features. To ensure a fair comparison of predictive performance across all methods, we standardized the input data using one‐hot encoding. The average performance across all datasets is shown in Figure 2a, while detailed metrics for each dataset are displayed in Figure 2b and Figure S1 (Supporting Information). Notably, diPaRIS outperforms state‐of‐the‐art methods across all six metrics when using standardized input data. **Figure** **3**a displays the distribution of ACC metrics for each method using this standardized sequence data. diPaRIS demonstrates the highest median ACC, followed by HDRNet, DeepRiPe, PrismNet, and DeepBind. Detailed performance metrics for AUC, AUPR, F1‐score, Precision, and Recall are provided in Figure S2 and Tables S9 to S14 (Supporting Information). Notably, diPaRIS achieves the highest median values across all these additional metrics. Furthermore, the range of metric values for diPaRIS is narrow, indicating more consistent performance compared to other models.

To highlight the benefits of incorporating structural features, we compared the performance of diPaRIS with and without using structural data. The results of ACC metrics are shown in Figure 3b (the results for other metrics are shown in Figure S3, Supporting Information). Across all datasets, incorporating structural features leads to significant improvements. The performances on LARP4‐HepG2^[^ ^22^ ^]^ and PABPC4‐K562^[^ ^23^ ^]^ datasets show the most improvement when structural features are included. Functional enrichment analyses on these two datasets (Figures S4 and S5, Supporting Information) indicate that the enriched functions are closely linked to specific RNA structures, such as binding to the polyA region of mRNA.^[^ ^24^ ^]^ Further, we analyzed the distribution of predicted scores of positive and negative samples by diPaRIS for these two datasets (Figure 3c). Compared to using only sequence data, integrating both sequence and structural data leads to better separation between positive and negative samples. This indicates that structural features improve the model's ability to predict binding sites. Prior studies^[^ ^14^, ^16^ ^]^ have primarily used icSHAPE‐seq data directly as input without effective encoding of RNA structural features or fully leveraging attention mechanisms. This approach limits the models' ability to fully leverage structural information and to focus on critical regions of the RNA, thereby constraining their predictive performance. To evaluate the contributions of icSHAPE‐DS and attention mechanisms to diPaRIS's performance, we tested diPaRIS with and without our proposed encoding scheme and attention mechanisms. The detailed performance metrics for each dataset are provided in Tables S3 to S8 (Supporting Information), while Table S15 (Supporting Information) presents the average performance across all datasets, showing that the attention mechanism has a more significant impact: removing attention leads to a more reduction in performance compared to removing icSHAPE‐DS.

We further explored the impact of structural information on proteins with distinct structural domains. The flag proteins in our datasets mainly contain seven conserved families of protein‐binding domains: G‐Patch domain, helicase domain, K homology (KH) RNA‐binding domain, RGG domain, RNA recognition motif (RRM) domain, WD40 domain, and zinc finger (ZnF) domain. The associations between the proteins and their respective protein‐binding domains are detailed in Table S16 (Supporting Information). Figure 3d illustrates the improvement in ACC between scenarios that utilize structural information and those that rely solely on sequence data. It reveals that proteins with G‐patch structural domains are the most significantly affected, followed by those with ZnF structural domains, while proteins with KH structural domains are the least affected. Notably, several proteins containing RRM domains also demonstrate a significant effect. The G‐patch domain is characterized by the tandem connection of the C‐terminal RRM domain and an N‐terminal unknown domain.^[^ ^25^ ^]^ Additionally, when combined with other RNA‐binding domains, ZnFs can enhance the affinity and specificity of RNA‐binding proteins.^[^ ^26^ ^]^ Thus, RRM‐containing proteins with a significant impact may possess similar combinatorial structural architectures, which could enhance their functional efficacy and lead to improved predictive performance.

### 2.5 Predicting RBP Binding Sites in Different Cell Lines

**Figure 4:**
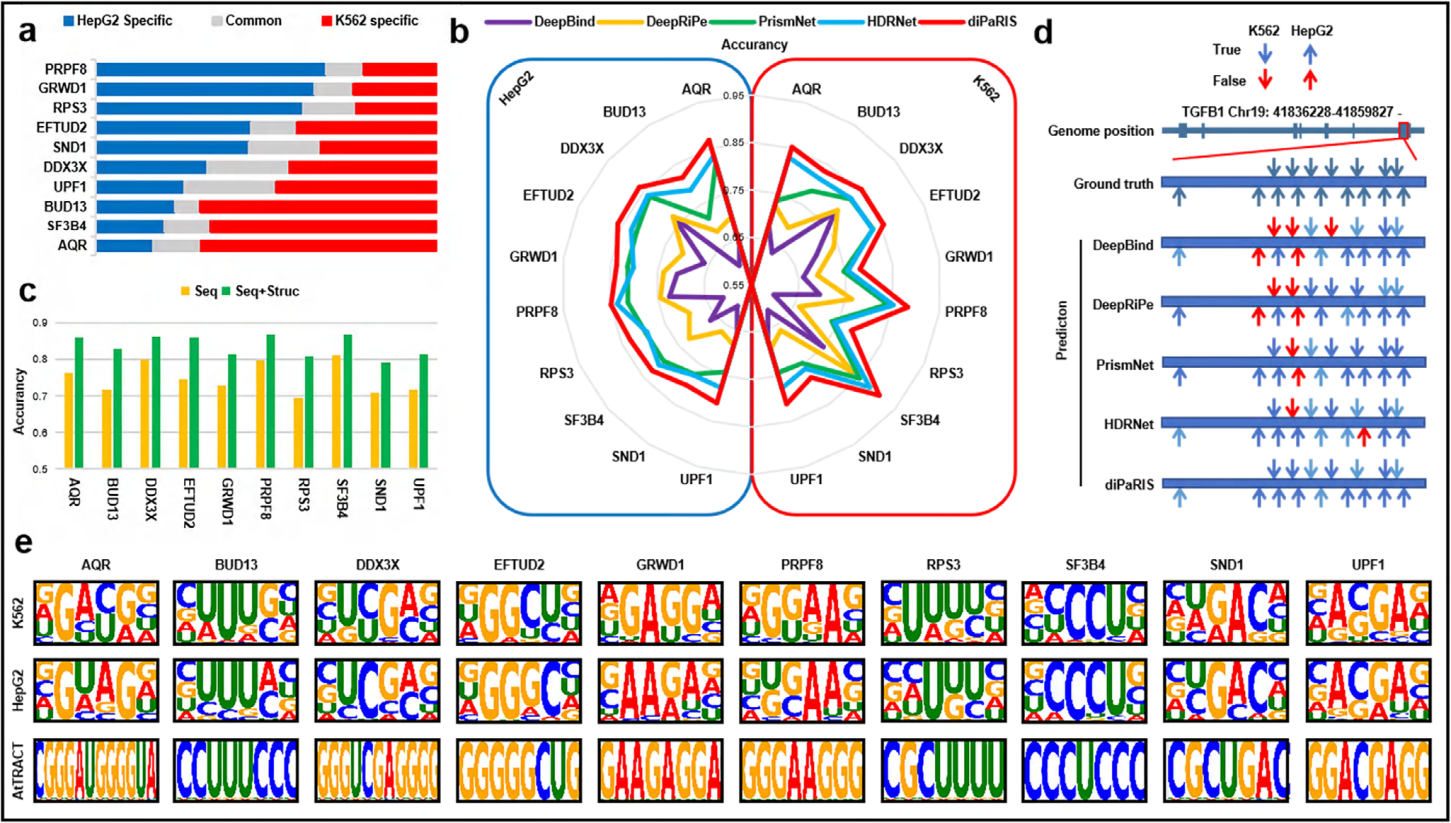
Predicting binding sites under different cell lines. a) Statistics on the proportion of binding sites under HepG2 and K562 cell lines. The blue part represents binding sites only under the HepG2 cell line. The red part represents binding sites only under the K562 cell line. The grey part represents common binding sites that appear under both cell lines. b) ACC performance of cross‐test on each dataset. The left side shows the performance under the HepG2 cell line while the right side shows the performance under the K562 cell line. c) ACC performance of cross‐test on each protein predicted by diPaRIS, whether using structural features. The green bars represent the performance using both sequence and structural features (Seq+Struc), while the yellow bars represent the performance using sequence features only (Seq). d) Predicted vs observed binding sites of DDX3X on the TGFB1 transcript. Arrows orientated downwards indicate binding sites under the K562 cell line while arrows orientated upwards indicate binding sites under the HepG2 cell line. Blue arrows indicate correctly predicted binding sites and red arrows indicate incorrectly predicted binding sites. e) Sequence motifs constructed from PWMs learned from samples by diPaRIS and PWMs obtained from the ATtRACT database.

RBP binding sites are known to be tissue‐specific and can vary under different cellular conditions.^[^ ^14^, ^16^ ^]^ Among our 44 datasets, 20 contain binding sites for the same ten proteins across either HepG2 or K562 cell lines. We analyzed these datasets to further investigate protein binding sites under different cellular conditions. The distribution of binding sites for the same proteins across different cell environments is shown in **Figure** **4**a. The proportion of common binding sites between the two cell lines is 15.8%, suggesting that most binding sites in our datasets are tissue‐specific and unique to a single cell line.

diPaRIS effectively learns the patterns of binding sites in different cell lines, resulting in more accurate predictions compared to existing methods. To assess its performance, we employed a cross‐test approach, training models on datasets from one cell line and evaluating them on datasets for the same protein in another cell line. The ACC result is shown in Figure 4b. diPaRIS achieves the highest accuracy in all datasets, with an average ACC exceeding 0.83 across all of them. The t‐test results indicate that diPaRIS significantly improves the ACC of cross‐tests in both HepG2 and K562 cell lines (p‐value < 0.05, Table S17). Detailed performance for the other metrics can be found in Figure S6 and Tables S18 to S23 (Supporting Information). diPaRIS consistently outperforms competing methods in AUC, AUPR, and F1‐score across all datasets. We conducted an independent cross‐cell line test for the comparative methods by predicting IGF2BP1 binding sites in the HepG2 cell line using a model trained on the IGF2BP1‐K562 dataset. As shown in Table S24 (Supporting Information), diPaRIS achieved the highest number of correct predictions. To further evaluate diPaRIS's cross‐cell line performance, we also tested diPaRIS's cross‐cell line performance by predicting ZNF800 binding sites in the HepG2 and HEK293 cell lines^[^ ^8^, ^27^ ^]^ using a model trained on the ZNF800‐K562 dataset. According to Table S25, the prediction accuracy for binding sites in both cell lines exceeded 71%. These results highlight diPaRIS's superior generalization performance.

To evaluate the contributions of icSHAPE‐DS and attention mechanisms to cross‐test performance, we tested diPaRIS with and without our proposed encoding scheme and attention mechanisms. The detailed performance metrics for each dataset are provided in Tables S18 to S23 (Supporting Information). Removing attention leads to a greater reduction in ACC, AUC, AUPR, and F1‐score compared to removing icSHAPE‐DS, indicating that the attention mechanism has a more significant impact. We further investigated the impact of structural features on the model's predictive ability. Cross‐tests using diPaRIS alternately with and without structural features indicate that including structural information significantly enhances predictions, with an average ACC increase of 11.82% across all datasets (Figure 4c). This result underscores the crucial role of structural information in accurately predicting RBP binding sites across different cell lines.

To further evaluate the performance of diPaRIS in clinically relevant scenarios, we conducted cross‐tests on molecular targets involved in tumor progression. DEAD‐box helicase 3 X‐linked (DDX3X) impacts various stages of the RNA life cycle and regulates the expression of hundreds of genes.^[^ ^28^ ^]^ Transforming growth factor‐β (TGFB) is a polypeptide that regulates many biological processes, and previous studies have found that TGFB1 can cause epithelial‐mesenchymal transition (EMT), leading to increased migration of cancer cells.^[^ ^29^ ^]^ Figure 4d illustrates the cross‐test results of predicted DDX3X binding sites on the TGFB1 transcript by all comparative methods. The immunoprecipitation experiment detects that the TGFB1 transcript harbours seven DDX3X binding sites in the K562 cell line and nine in the HepG2 cell line.^[^ ^8^ ^]^ diPaRIS predicts all binding sites in both cell lines. In contrast, other comparative methods produce incorrect predictions in both K562 and HepG2 cell lines, with varying accuracy across binding sites. For example, the region Chr19: 41,859,366‐41,859,466 is only predicted by HDRNet and diPaRIS in HepG2 cell line; in K562 cell line, only diPaRIS makes a correct prediction for this region. To interpret the predictions of comparative methods, we generated attribution maps (Experimental Section) for this region, as illustrated in Figures S7 and S8 (Supporting Information). The weights predicted by diPaRIS for certain intervals significantly differ from those of the comparative methods, which may contribute to the differences in predictions. HDRNet is excluded from generating the attribution map due to its low feature resolution, which is insufficient for analyzing weights at the single‐nucleotide level.

Analysis of motifs generated by diPaRIS indicates its ability to recognize and learn conserved binding site patterns for a given protein across different cell lines. We extracted learning weights from the trained model and constructed position weight matrices (PWM, Experimental Section).^[^ ^30^ ^]^ We selected motifs with the strongest positional specificity to compare between cell lines and also contrasted them with motifs from the ATtRACT database.^[^ ^31^ ^]^ The elucidated sequence motifs are illustrated in Figure 4e, revealing that sequences attributed to the same protein but derived from different cell lines share a high degree of similarity. This similarity is less pronounced in motifs from distinct proteins, reflecting RBPs' conserved molecular functions under different cellular conditions. Compared to motifs generated by other methods (Figures S9 to S12, Supporting Information), those produced by diPaRIS show more highly weighted loci, indicating greater specificity.

### 2.6 Cross‐Protein Predictions Reflect Protein Conservation

**Figure 5:**
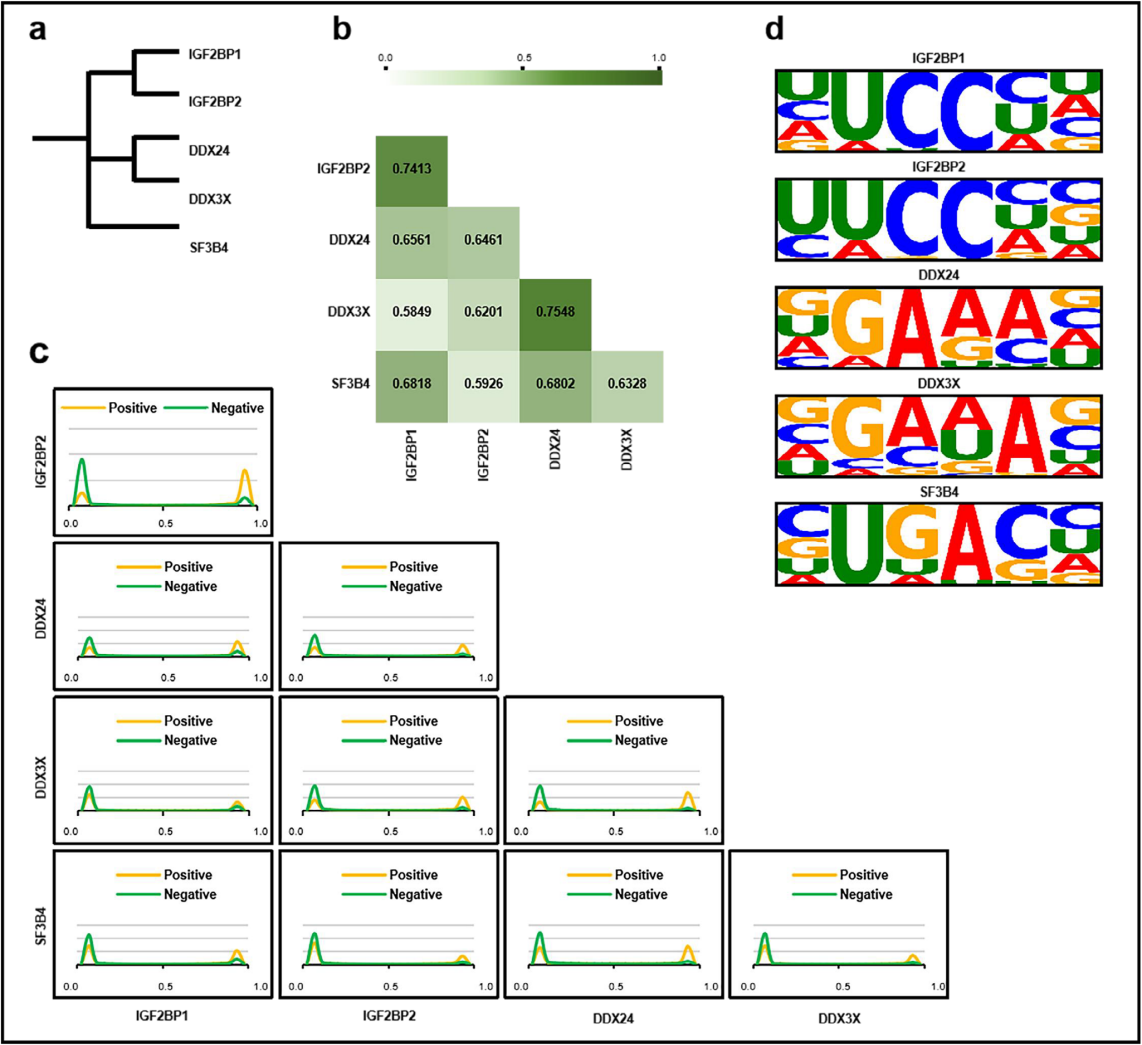
Cross‐test performance of diPaRIS on protein pairs. a) homologous relationships of selected proteins. b) ACC scores of diPaRIS for each protein pair in cross‐test scenarios. c) The distribution of predicted scores for cross‐test samples of each protein under the K562 cell line. d) Sequence motifs constructed from PWMs learned from cross‐test samples by diPaRIS.

Analysis of motifs generated by diPaRIS indicates that it can effectively recognize and learn conserved patterns of binding sites for different proteins. Previous studies suggested that proteins with close homologous relationships exhibit more significant functional similarities.^[^ ^32^ ^]^ To test it, we made predictions using diPaRIS in the K562 cell line and observed that the binding site patterns identified by diPaRIS are consistent with the expected conservation. Among the 34 flag proteins in our datasets, we identified two pairs belonging to the same family: IGF2BP1 and IGF2BP2 from the IGF2BPs family, which share high sequence similarity,^[^ ^4^ ^]^ and DDX24 and DDX3X from the DDX family, which also exhibit greater sequence similarity than most proteins.^[^ ^3^ ^]^ All datasets were derived from the K562 cell line. To serve as a control, we included an additional dataset for SF3B4,^[^ ^33^ ^]^ a splicing‐related protein that lacks clear association with the IGF2BPs and DDX protein families. The homologous relationships among these proteins are depicted in **Figure** **5**a.

We employed a cross‐test approach, training models on datasets from one protein and testing on datasets from another. Figure 5b displays the predicted ACC metrics of diPaRIS for each protein pair. The results indicate that diPaRIS achieves higher ACC scores for proteins with closer homology, such as DDX24‐DDX3X and IGF2BP1‐IGF2BP2, with scores of 0.7548 and 0.7413, respectively. For protein pairs with more distant homology, their ACC scores do not exceed 0.7. Specifically, for the pairs DDX3X‐IGF2BP1 and SF3B4‐IGF2BP2, the ACC scores fall below 0.6, reflecting lower predictive performance. The detailed cross‐test performances of all metrics for all comparative methods are shown in Tables S26 to S31 (Supporting Information). In addition to ACC, diPaRIS also shows significant predictive differences between high‐ and low‐homology protein pairs across multiple metrics, including AUC, AUPR, and F1‐score, effectively capturing the conservation of protein‐RNA binding compared to other state‐of‐the‐art methods. We also performed an independent test to predict binding sites of an additional family member IGF2BP3 in the HepG2 cell line, using models trained on either the IGF2BP1‐K562 or IGF2BP2‐K562 datasets. The results show that diPaRIS achieves superior generalization performance compared to the other methods (Table S32, Supporting Information).

We further analyzed the distribution of predicted scores for each pair of proteins in the K562 cell line. As presented in Figure 5c, for protein pairs with closer homology, the predicted scores of positive samples and negative samples show better separation than the protein pair with more distant homology.

We also constructed sequence motifs by extracting learning weights from diPaRIS and selected those with the strongest positional specificity for comparison between proteins, allowing to visualize the conservation of binding sites among proteins with varying levels of homology. The sequence motifs generated by diPaRIS are shown in Figure 5d, while those from other methods are provided in Figure S13 (Supporting Information). The results indicate that sequence motifs of proteins within the same family are more similar to each other compared to those from different families. Notably, the sequence motif of SF3B4 shows no significant similarity with any other proteins, indicating that proteins with distant homology have non‐similar binding site patterns, resulting in lower ACC performance in cross‐tests.

### 2.7 Predicting the Potential Effects of SNV on Protein Binding

Single nucleotide variants (SNVs), a primary type of genetic variant, represent enduring alterations in the nucleotide composition of DNA sequences constituting genes.^[^ ^34^ ^]^ These changes may disrupt RBP recognition of RNA substrates, potentially impairing gene function, and contributing to disease progression.^[^ ^35^ ^]^ We utilized diPaRIS to quantify and analyze the potential effects of SNVs on protein‐RNA interactions through a case study involving DDX3X and TGFB1. Both of these molecules exhibit significant variant frequencies in leukaemia, and their associations with cancer pathology have been well‐documented.^[^ ^36^ ^]^ However, whether the interactions between these two genes affect leukemia progression has not been reported.

**Figure 6:**
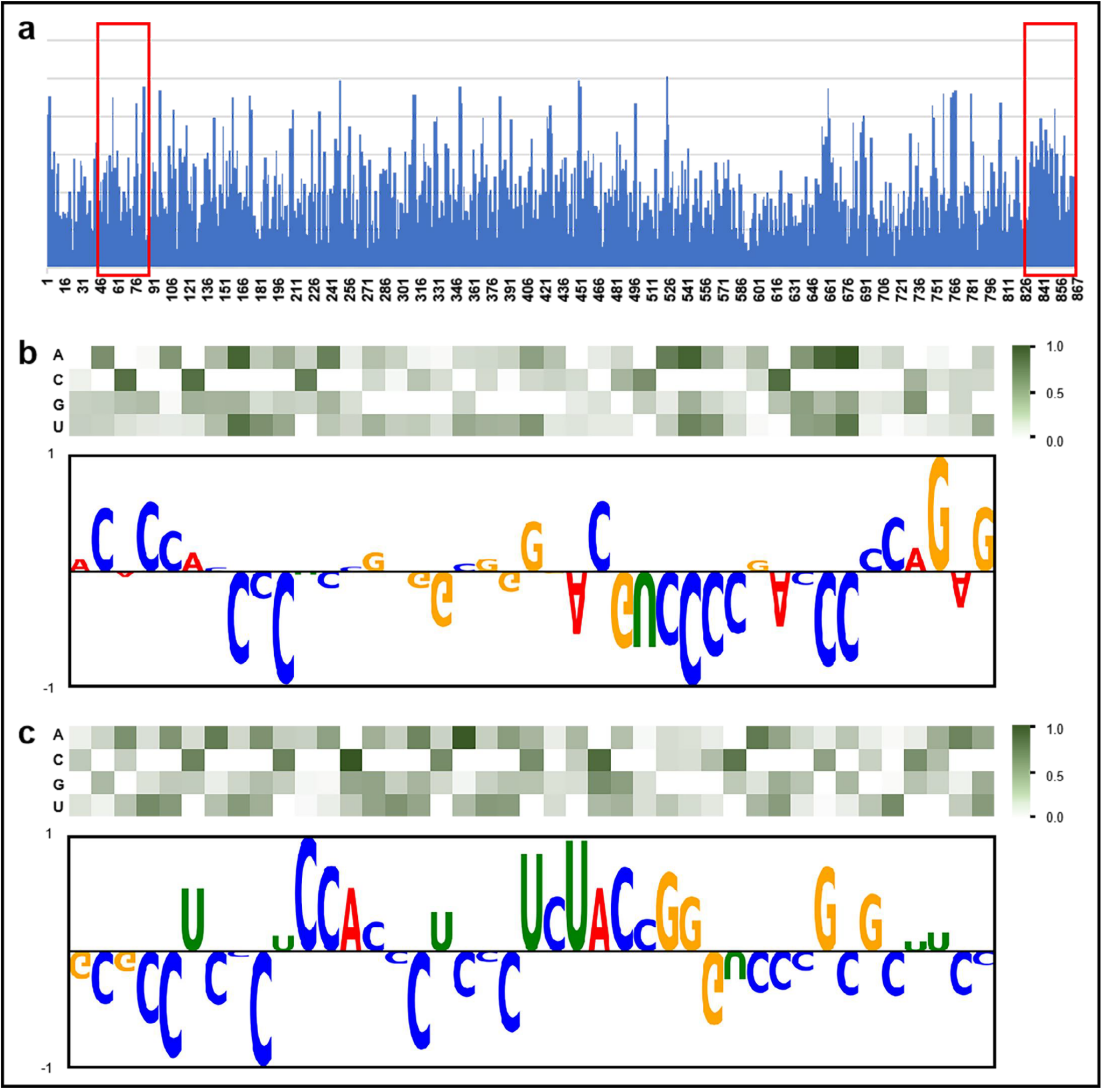
Predicting the potential impact of genetic variants in the 3' UTR region of TGFB1 on DDX3X binding. a) Non‐directional mutation score at each nucleotide in the 3' UTR region of TGFB1 on DDX3X binding. The two regions with the highest cumulative scores, each 41 nucleotides in length, are highlighted in red boxes. b) Region 1 (positions 49‐89): Mutation scores for different types of single‐nucleotide mutations constructed from the PWM. The attribution map displays the contribution weight of each nucleotide in the original TGFB1 3' UTR sequence to DDX3X binding. c) Region 2 (positions 827‐867): Mutation scores for different types of single‐nucleotide mutations constructed from the PWM. The attribution map displays the contribution weight of each nucleotide in the original TGFB1 3' UTR sequence to DDX3X binding.

To assess the impact of SNVs on the binding affinity of DDX3X to the TGFB1 transcript, we utilized diPaRIS to compute the nucleotide mutation scores within the TGFB1 3' UTR (Experimental Section). Previous studies have shown that raw eCLIP‐seq peaks, which correspond to binding sites, typically span approximately 40 nucleotides.^[^ ^2^, ^8^ ^]^ Therefore, we aggregated the non‐directional mutation scores for each nucleotide within subsequences of 41 nucleotides to identify regions highly susceptible to genetic variants. The two regions with the highest mutation scores are highlighted in red in **Figure** **6**a. Region 1 (positions 49‐89) lies within 100 nt of the coding start site, potentially encompassing cis‐acting regulatory elements such as enhancers.^[^ ^37^ ^]^ Region 2 (positions 827‐867) is adjacent to the transcription start site, possibly influencing alternative splicing of transcripts.^[^ ^38^ ^]^

We further analyzed specific SNVs with the most pronounced effects on protein binding. Figure 6b shows the impact of different single‐nucleotide variants in Region 1 on DDX3X binding. Variants at positions 56, 75, and 83, where cytosine is replaced by adenine, exhibit the most significant reduction in DDX3X binding affinity. We also extracted learning weights from the trained model to construct an attribution map for the original sequence of Region 1. The map reveals that cytosine and guanine are the most frequent nucleotides, appearing 20 and 12 times, respectively. In contrast, adenine and uracil occur seven and two times, respectively. Nucleotides 56, 75, and 83 exhibit significant negative weights, indicating that they are critical for maintaining binding stability and have a substantial impact when mutated.

A similar analysis was performed for Region 2 on DDX3X binding. Figure 6c shows the effect of different SNVs in Region 2. Variants at positions 839 and 850, where adenine is replaced by cytosine, and at position 844, where cytosine is replaced by adenine, result in the most significant changes in binding affinity. The attribution map for the original sequence of Region 2 shows that cytosine is the most abundant nucleotide with 24 occurrences, followed by uracil and guanine with eight and seven occurrences, respectively. Adenine is the least abundant nucleotide with only two occurrences. Nucleotide 844 has a negative weight in the attribution map, consistent with observations from Region 1. In contrast, the higher positive weights of nucleotides 839 and 850 underscore the importance of these positions for DDX3X recognition, given the scarcity of adenine in this region.

### 2.8 Predicting the Potential Impact of A‐to‐I Modifications on Disease Development

Utilizing diPaRIS, we investigated the impact of adenosine‐to‐inosine (A‐to‐I) RNA editing events on protein‐RNA binding affinities in the K562 cell line, aiming to understand how these modifications might influence disease progression. A‐to‐I RNA editing, catalyzed by the adenosine deaminase RNAs specific (ADARs) family of enzymes, is a key gene regulatory mechanism that diversifies the transcriptome.^[^ ^39^ ^]^ A‐to‐I editing is notably altered in various cancers, including leukemias.^[^ ^40^ ^]^ We gathered RBP‐associated RNA editing sites in K562 cell line from prior studies.^[^ ^8^, ^41^ ^]^ Since inosine is primarily interpreted as guanosine by cellular systems, A‐to‐I editing corresponds to an A‐to‐G transition. We predicted a total of 905 editing sites in the K562 cell line using diPaRIS. The prediction results indicate that 46 binding sites are significantly altered before and after editing. Detailed annotations and predicted scores are provided in Table S33 (Supporting Information).

**Figure 7:**
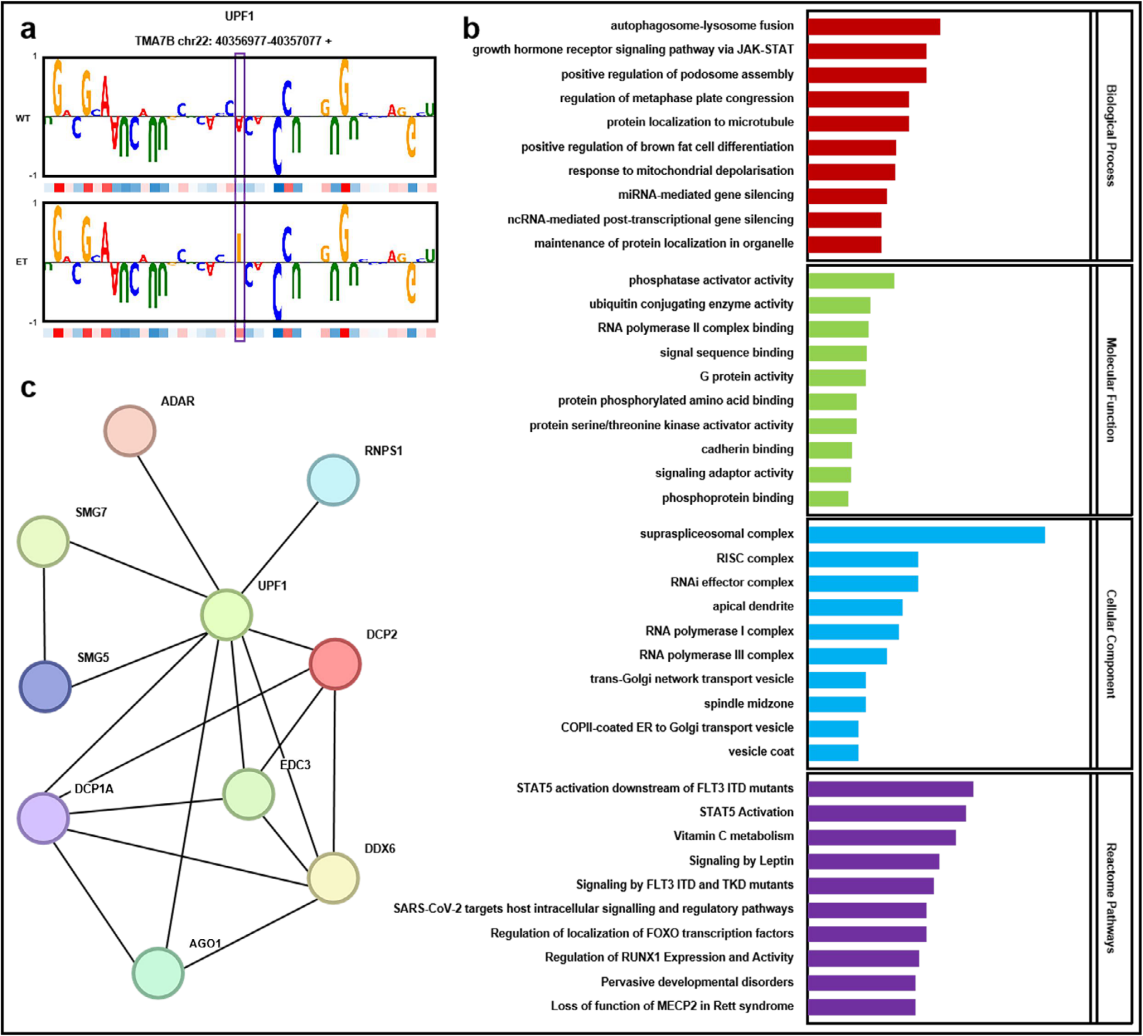
Predicting the potential impact of RNA editing on UPF1 binding and its implications in leukaemia using diPaRIS. a) Attribution map constructed on the PWM learned around 40357027 on the Chr22 sense strand for UPF1 binding. WT (wild type) indicates the unedited RNA sequence, whereas ET (edited type) reflects the sequence variant generated by RNA editing at this position. Purple box indicates the RNA editing site, where the weight changes significantly before and after editing. b) Gene Ontology and Reactome pathway enrichment analysis of genes whose transcripts bind to UPF1. c) Interaction network between UPF1 and UPF1‐binding genes.

We then examined how these A‐to‐I editing events affect protein binding sites. Among all the proteins, UPF1 RNA helicase and ATPase (UPF1) had the highest number of significantly altered editing sites, with 14 showing notable changes in binding affinity before and after editing. For example, one altered site is located at position 40,357,027 on the Chr22 sense strand. Before editing, diPaRIS predicted a binding score of 0.1582 for this site, which increases to 0.5658 after editing, indicating a stronger interaction with UPF1 post‐editing. The attribution maps of this site, shown in **Figure** **7**a, reveal that the model‘s prediction weights for the site and its neighboring nucleotides changed significantly before and after editing. This suggests that the editing event substantially alters the binding affinity of UPF1, potentially impacting the gene's regulatory mechanisms and contributing to disease progression.

To further investigate the functional implications of altered binding sites, we performed Gene Ontology (GO) and Reactome pathway enrichment analyses on genes whose transcripts bind to UPF1. The top 10 enriched terms are shown in Figure 7b. In the biological process category, genes are enriched in the growth hormone receptor signaling pathways via JAK‐STAT (GO:0060397). For molecular function, the genes show enrichment in ubiquitin‐conjugating enzyme activity (GO:0061631). In the cellular component category, the genes are involved in the RNA‐induced silencing complex (RISC) (GO:0016442). These GO terms suggest a link to immune‐related functions. Reactome pathway analysis further reveals enrichment in pathways such as STAT5 activation downstream of FLT3 ITD mutants (R‐HSA‐9702518) and general STAT5 activation (R‐HSA‐9645135), which are known to play roles in various biological processes, including immunity.^[^ ^42^ ^]^ These functional analyses demonstrate that transcripts binding to UPF1 mainly regulate tumour progression through immune‐related pathways.^[^ ^43^ ^]^ Consequently, the binding sites of the UPF1 protein may influence the immune system, potentially affecting leukaemia progression.

To identify potential genes affected by altered UPF1 function, we constructed an interaction network using UPF1‐binding genes (Experimental Section). As shown in Figure 7c, the nine genes most closely associated with UPF1 include ADAR, AGO1, DCP1A, DCP2, DDX6, EDC3, RNPS1, SMG5, and SMG7. Previous studies have suggested that these genes are closely linked to tumorigenesis. For instance, ADAR, known for its role in catalyzing A‐to‐I editing, is overexpressed in leukaemia and is correlated with increased disease progression.^[^ ^44^ ^]^ Argonaute RISC component 1 (AGO1) is a key protein in miRNA processing, and the importance of AGO1 post‐translational modifications in cancer has been reported.^[^ ^45^ ^]^ DEAD‐box helicase 6 (DDX6) has been identified as a target gene for genomic alterations in leukemia.^[^ ^46^ ^]^ These results suggest that the binding sites of UPF1 may regulate these genes, potentially influencing leukemia development and progression.

## 3 Discussion

Proteins are extensively involved in and regulate the entire biological process of RNA, from transcription to degradation and extinction. Any disruption in these regulatory mechanisms can precipitate the onset and progression of a myriad of diseases. Studying protein‐RNA interactions is crucial for understanding their role in biological processes. CLIP sequencing technology allows high‐throughput detection of RBP binding sites at the transcript level, providing a basis for predicting protein‐RNA interactions through computational calculations. SHAPE sequencing provides structural information on binding sites in vivo. However, current computational methods have limitations in describing structural patterns of binding sites without proper encoding, which is not conducive to comprehensive model learning to extract structural information from binding sites. Such shortcomings pose significant challenges to better predicting specific interactions between RNA and proteins in diverse tissues.

We proposed a novel deep learning method, diPaRIS, to predict RBP binding sites. Specifically, we utilized sequence and structure information to characterize the properties of the binding sites. To comprehensively represent dynamic RNA structures in vivo, we developed a novel encoding scheme called icSHAPE‐DS. diPaRIS employs a U‐shaped deep residual neural network for feature learning, and attention mechanisms help the model capture both proximal and distant nucleotide dependencies. Finally, diPaRIS predicts binding scores using an MLP based on the features aggregated through pyramid pooling and global expectation pooling.

We benchmarked diPaRIS against four state‐of‐the‐art methods across 44 datasets involving different proteins and cell conditions. Our method consistently outperforms existing methods across multiple evaluation criteria. Furthermore, when the trained model is applied to datasets containing the same proteins across different cell lines, diPaRIS demonstrates superior performance compared to state‐of‐the‐art methods. Additionally, diPaRIS also offers interpretable analysis by learning sequence binding motifs and generating attribution maps. Our method effectively captures motifs that reflect the conservation of protein‐RNA binding across datasets from different cell lines and proteins. Furthermore, diPaRIS also interprets the potential impact of genetic variants on protein‐RNA interactions by generating attribution maps. Our investigation provides insights into molecular interactions and functional mechanisms altered by genetic variants in complex diseases.

Overall, diPaRIS comprehensively characterizes RBP binding sites using a novel structural encoding scheme. As a new deep learning method, diPaRIS provides more accurate predictions than existing methods and robustly reflects the conservation of protein‐RNA binding. Furthermore, our model offers valuable insights into the molecular underpinnings of binding site interactions and their implications in human disease pathogenesis.

## 4 Experimental Section

### Data Processing

Datasets were constructed by employing two complementary sequencing technologies: eCLIP‐seq for the generation of sequence features, and icSHAPE‐seq for the characterization of structural features. To ensure comprehensive integration of sequence and structural features, each analyzed binding site is covered by both datasets. First, eCLIP‐seq data were sourced from the ENCODE database^[^ ^20^ ^]^ in BED format^[^ ^8^ ^]^ to capture transcriptome‐wide in vivo binding sites. Binding sites were rigorously selected based on stringent enrichment criteria relative to input controls and reproducibility across biological replicates in HepG2 and K562 cell lines,^[^ ^8^ ^]^ ensuring robust, and reliable detection. Simultaneously, icSHAPE‐seq data from Zhang's lab^[^ ^14^ ^]^ provided single‐nucleotide resolution of RNA structural contexts in wiggle format. In total 223 eCLIP‐seq datasets, which include data on 844,854 binding sites, as well as two corresponding icSHAPE‐seq datasets for these cell lines, were collected. All data were processed using the human genome GRCh37/hg19 as a reference^[^ ^21^ ^]^ to ensure genomic alignment consistency. Next, sequences corresponding to eCLIP‐seq annotations were extracted from the reference genome and paired with structural profiles from icSHAPE‐seq. Following stringent filtering, a total of 302,146 binding sites were retained for further analysis. To standardize input for model training, binding sites were uniformly trimmed to 101 nucleotides in length,^[^ ^11^, ^14^, ^16^ ^]^ aligning with established protocols. To fully capture the complexity of true negative interactions as much as possible, random sampling from non‐binding regions across all datasets was applied to generate negative samples. Parallel processing ensured consistency between positive samples (binding sites) and negative samples (non‐binding regions), with equal numbers of both labeled as ‘1’ and ‘0’, respectively. Finally, to ensure statistical robustness and minimize sampling bias, only datasets with >2000 samples were retained for model training. This yielded 44 datasets comprising 464,112 samples, each labeled as “flag protein ‐ background cell line” (e.g., IGF2BP1‐K562). These datasets encompass binding sites for 34 RBPs in HepG2 or K562 cell lines and are summarized in Table S1 (Supporting Information).

*Feature encoding*: To enable the deep learning model to learn biological insights from sequencing data, the sequence and structure of the binding sites were encoded using two distinct schemes. Sequence information was encoded using the conventional one‐hot scheme,^[^ ^11^, ^12^, ^14^ ^]^ where each nucleotide (A, C, G, U) is represented by a 4D vector, producing a 101×4 matrix for each sequence. All matrix elements were initialized to zero, with the element corresponding to the nucleotide at each position set to 1.

For structural information, a novel encoding scheme called icSHAPE‐DS (Figure 1a) was developed based on SHAPE sequencing, which detects dynamic RNA structures in vivo across different alternative splicing transcripts that were mapped to the reference genome. The icSHAPE‐DS scheme encodes each sample's structure as a 100×7 matrix, initialized with zeros. It captures key aspects of RNA folding dynamics, including maximum probability structures, changes in structure formation probability, and information entropy, using a 7D vector to represent each pair of adjacent nucleotides.

*Maximum probability structures*: Maximum probability structures were extracted from icSHAPE‐seq data, which were experimentally validated and better reflect physiological conditions than traditional minimum free energy predictions. Nucleotides with icSHAPE values above 0.233 were defined as forming loops and assigned 1 to the corresponding position.^[^ ^14^ ^]^

*Changes in structure formation probability*: Alternative splicing transcripts contribute to the formation of dynamic RNA structures, a crucial characteristic captured during RNA structure experiments in vivo. Four dimensions was used to capture changes in the probability of structure formation between adjacent nucleotides, as well as to indicate the presence or absence of nucleotide data. For two consecutive nucleotides, dimensions 1 and 2 record which nucleotide had a higher icSHAPE‐seq value. If the first nucleotide had a higher value than the second, the dimensions were set to [1, 0]; otherwise, they were set to [0, 1]. These dimensions reflect whether the probability of forming a structure increases or decreases between consecutive nucleotides. Dimensions 3 and 4 record missing data. If only the first nucleotide's value was missing, the dimensions were set to [0, 1]; if only the second nucleotide's value was missing, they were set to [1, 0]. If both nucleotides were missing, all four dimensions were set to ‐1 to indicate the absence of structural information. This ensures that consecutive missing nucleotides were clearly distinguished from valid ones.

*Information entropy*. Due to the limited sequencing coverage of icSHAPE‐seq data, an additional dimension was included to capture the information entropy for each nucleotide pair, ensuring that regions not fully covered by sequencing are still accurately represented in the encoded features.

### diPaRIS

The method integrates three modules–feature encoding, feature learning, and binding site prediction, as illustrated in Figure 1.

*Feature* *Encoding*: First, sequence and structural features of binding sites were extracted from sequencing data, following the approach described earlier. After extraction, the convolution module was used to process and align these features. The CNN applied a weight‐sharing strategy to extract various feature weights from the original encoding using multiple detectors. This method, widely used in previous studies,^[^ ^11^, ^12^, ^14^, ^16^ ^]^ successfully localizes motifs and extracts sample attribution maps. To capture more relevant features, the extracted features were fed into a Bi‐LSTM module. LSTM networks were known for learning long‐range dependencies in features.^[^ ^12^, ^47^ ^]^ The output of the Bi‐LSTM was used as a weight matrix to adjust the output weights of each CNN detector.^[^ ^48^ ^]^ After this processing, the dimensions of sequence and structural features were aligned and concatenated into a unified feature matrix.

*Feature Learning*: A U‐shaped deep neural network was employed for feature learning (Figure 1b). The network consists of four modules: downsampling, jump connection, bottleneck, and upsampling.

*Downsampling*: Downsampling connects local sub‐fragment features of the binding site to form new binding site fragment features over a long distance.
  First, the features are fed into a residual block with a bottleneck structure. The bottleneck design reduces computational load, while residual skip connections allow gradients to propagate directly back to earlier layers, effectively mitigating the vanishing gradient problem in deep networks. Additionally, the non‐linear decoupling characteristic of the bottleneck structure and the cross‐layer information fusion provided by the residual blocks enable the fine extraction of more complex features from local information. The outputs were fed into the jump connection module and a max‐pooling layer to reduce feature redundancy by decreasing the sampling amount. After pooling, the features were input to the next downsampling module. In each module, the number of feature channels was doubled and halve the feature length.
*Jump connection*: To enhance the localization accuracy of high‐impact regions during the up‐sampling process and to better differentiate surrounding low‐impact regions, features were fed into a module that adjusts feature weights using a multi‐head attention mechanism.^[^ ^14^ ^]^ The output of the Transformer adjusts the weights of the Downsampling module. The features were then input to the upsampling module.
*Bottleneck*: After three consecutive downsampling modules, the features were fed into the bottleneck module. Given that the features integrate both sequence and structural information, diPaRIS employed two successive residual block with a bottleneck structure to combine contextual information, resulting in a richer and more efficient feature representation. The features were then input to the upsampling module.
*Upsampling*: Upsampling amplifies high‐impact regions in the binding site to form new, distinguishable binding site fragment features. First, input features were fed into the transposed convolutional layer for expansion. Then, the output was concatenated with the feature from the jump connection module. The features were then fed into a residual block with a bottleneck structure, which amplifies impactful signals locally before passing them to the next upsampling module. At each upsampling step, the number of feature channels was halved and double the feature length. After three successive upsampling modules, the features were reshaped to the original input dimensions, integrating multi‐scale features while preserving feature details and maintaining the symmetry of the network structure.

*Binding Site Prediction*: The binding site prediction was based on features learned by the U‐shaped network, which were further refined through a pyramid pooling module. This module used three different scales of average pooling layers to capture distribution patterns of features across various regional scales. Information was aggregated by concatenating pooled vectors from different regions. Finally, a global expectation pooling layer^[^ ^19^ ^]^ was used to classify the samples. The pooled features were then passed through an MLP for nonlinear transformation, with predicted scores calculated using a softmax function. Samples with predicted scores above 0.5 were classified as binding sites, while those at or below 0.5 were classified as non‐binding sites (Figure 1c).

*Parameter setting* In the feature alignment phase, sequence features were extracted using 64 2D convolution kernels of size (6, 4) and structural features were extracted using 64 2D convolution kernels of size (10, 7). After extraction, sequence and structure features were sampled using a 32‐unit Bi‐LSTM.

During feature learning, the downsampling process uniformly uses 2D convolution kernels of sizes (1, 1) or (3, 3) with a pooling size of 2, and the number of channels was 32, 64, and 128. Within each residual block, the input feature map first passes through a (1, 1) convolution layer to create a shortcut connection. It then goes through two (3, 3) convolution layers where the intermediate channel count was reduced to one‐quarter. Finally, another (1, 1) convolution layer restores the channel, and the output is added to the shortcut connection to form the final output. The upsampling process follows a similar architecture, using 1D convolution kernels of sizes 1 and 3, along with transposed convolution kernels of size 3 and stride 2. The number of channels sequentially decreases to 128, 64, and 32. The jump connection module had the same number of attention heads and feature channels. To avoid overfitting, dropout layers with a rate of 0.3 were added after the transformer. The 1D and 2D convolution kernels coexist in the bottleneck module with 256 channels.

At the binding site prediction stage, the processed features were pyramid‐pooled using three different scale pooling layers, sampling the features of each channel as vectors of length 1, 5, and 25, respectively.

After pooling, the features were concatenated for global expectation pooling. The pooled features were then passed through an MLP with four fully connected layers, where the number of units was sequentially halved. Finally, a softmax activation function was applied to produce the output. Details of the parameter settings can be found in Table S34 (Supporting Information).

The model was trained using the TensorFlow 2.10.1 environment. The network was trained using the Adam optimizer with a batch size of 16 and an initial learning rate of 0.0005. The learning rate was dynamically adjusted based on epoch loss to address optimization challenges in early training.

*State‐of‐the‐art methods*: To elucidate the effectiveness of the model, diPaRIS was compared with several deep learning models and machine learning algorithms as follows.

*DeepBind*. This deep learning model predicts RBP binding sites by leveraging proximal sequential dependencies extracted using CNNs.^[^ ^11^ ^]^ DeepBind visualized a weighted ensemble of PWMs, which were utilized to uncover the regulatory roles of RBPs in alternative splicing and analyze disease‐associated genetic variants.
*DeepRiPe*. The model integrates CNNs and RNNs to capture both proximal and distal sequential dependencies, thereby enhancing the prediction of binding sites.^[^ ^12^ ^]^ DeepRiPe identifies sequence motifs within broader transcript context patterns consistent with known studies and offers interpretability for assessing the potential impact of sequence variants on binding sites.
*PrismNet*. This model leverages RNA structural data in vivo to predict RBP binding sites with high accuracy.^[^ ^14^ ^]^ It employed a residual CNN‐based deep learning network to capture nucleotide dependencies, incorporating a squeeze‐and‐excitation (SE) module to enhance feature extraction across various channels.^[^ ^48^ ^]^ PrismNet identified enrichment among dynamic RBP binding sites for structure‐altering variants (riboswitches), potentially linking genetic diseases to dysregulated RBP binding.
*HDRNet*. This model integrates k‐mer embedding sequences with RNA structures in vivo to enhance the identification of RBP binding sites.^[^ ^16^ ^]^ HDRNet used hierarchical multi‐scale residual networks to understand the contextual dependencies between nucleotides and then classifies samples by stacking several pyramid convolutional blocks. While HDRNet offers valuable insights into the pathological mechanisms of RNA‐RBP interactions, its use of k‐mer embedding features results in lower resolution, limiting its ability to analyze weight changes at the single‐nucleotide level.

### Generating sequence motifs, attribution maps and calculating mutation score

In diPaRIS, the one‐hot encoding scheme was used to capture sequence information. Each nucleotide was independently coded by its position and species. The CNN used different detectors to extract distinct binding site patterns from the samples. For the *i* ^*th*^ weight detector, the position was identified with the highest weight in each sample and extract a subsequence based on the length *l* of the weight detector. By computing the proportions of each nucleotide species in the subsequences obtained from all samples, a length‐*l* PWM was generated. Graphing this matrix offered a visual representation of the *i* ^*th*^ binding site pattern.^[^ ^31^ ^]^ MEME Suite was utilized^[^ ^49^ ^]^ for enrichment analysis, motif comparison, and other analyses related to the generated motifs.

For the *i* ^*th*^ sample, the weight detectors assign different weights to each nucleotide according to its position. Summing the weights of all the weight detectors at each nucleotide of the sample gave a position weight matrix of length 101. Graphing this matrix provided a visual representation of the attribution map for the *i* ^*th*^ sample.^[^ ^12^ ^]^

The mutation score was calculated using the PWM derived from the attribution map.^[^ ^12^, ^50^ ^]^ First, the PWM was computed for the original sample. Next, the species of each nucleotide in the sample were systematically varied, and the PWM was recalculated for each variant. For each position, the PWM was calculated three times, once for each of the three alternative nucleotide species. The mutation score for each species was defined as the absolute value of the difference between the original PWM and the PWM for the variant at the same position. The non‐directional mutation score was obtained by summing the mutation scores for all three nucleotide species at the variant position.

*Function enrichment analysis and interaction network construction*: Function enrichment analysis includes Gene Ontology and Reactome pathway enrichment analysis performed using the PANTHER knowledgebase.^[^ ^51^ ^]^ The database was updated on October 9, 2023. A list of genes was input using the gene symbol specification. Enrichment was evaluated using Fisher's exact test, with p‐values adjusted by the false discovery rate (FDR). The analysis included Biological Processes, Molecular Functions, and Cellular Components, as well as Reactome Pathways. Results were sorted by fold enrichment in descending order.

An interaction network was constructed for UPF1‐binding genes in the K562 cell line using STRING.^[^ ^52^ ^]^ A gene list, defined by gene symbols, was entered into the STRING database. The interactions of the top ten associated genes, including UPF1 itself, which was the common target among these binding genes, were visualized.

## Conflict of Interest

The authors declare no conflict of interest.

## Author Contributions

J.X.W., C.Q.L., and L.S.Z. conceived and designed the project. L.S.Z. conducted the analyses. L.S.Z., H.D.L, and J.X.W. wrote the paper, which was revised and proofread by X.Q.P., F.G., H.D.L., and J.X.W. All authors approved the final manuscript.

## Supporting information

Supporting Information

## Data Availability

The data that support the findings of this study are openly available in ENCODE at https://www.encodeproject.org/, reference number 8.
