# Supplementary material for: diPaRIS: Dynamic and Interpretable Protein‐RNA Interactions Prediction With U‐Shaped Network and Novel Structure Encoding
Source: Adv Sci (Weinh). 2025 Aug 29;12(38):e06314. doi: 10.1002/advs.202506314 (PMC12520504; doi:10.1002/advs.202506314)
Supplement: Supplementary file 1 — Supporting Information [file ADVS-12-e06314-s001.docx]

Supporting Information

**diPaRIS: Dynamic and Interpretable Protein-RNA Interactions Prediction with U-shaped Network and Novel Structures Coding**

*Lishen Zhang, Chengqian Lu, Xiaoqing Peng, Fei Guo, Hongdong Li*, and Jianxin Wang**

## Supplementary Figures


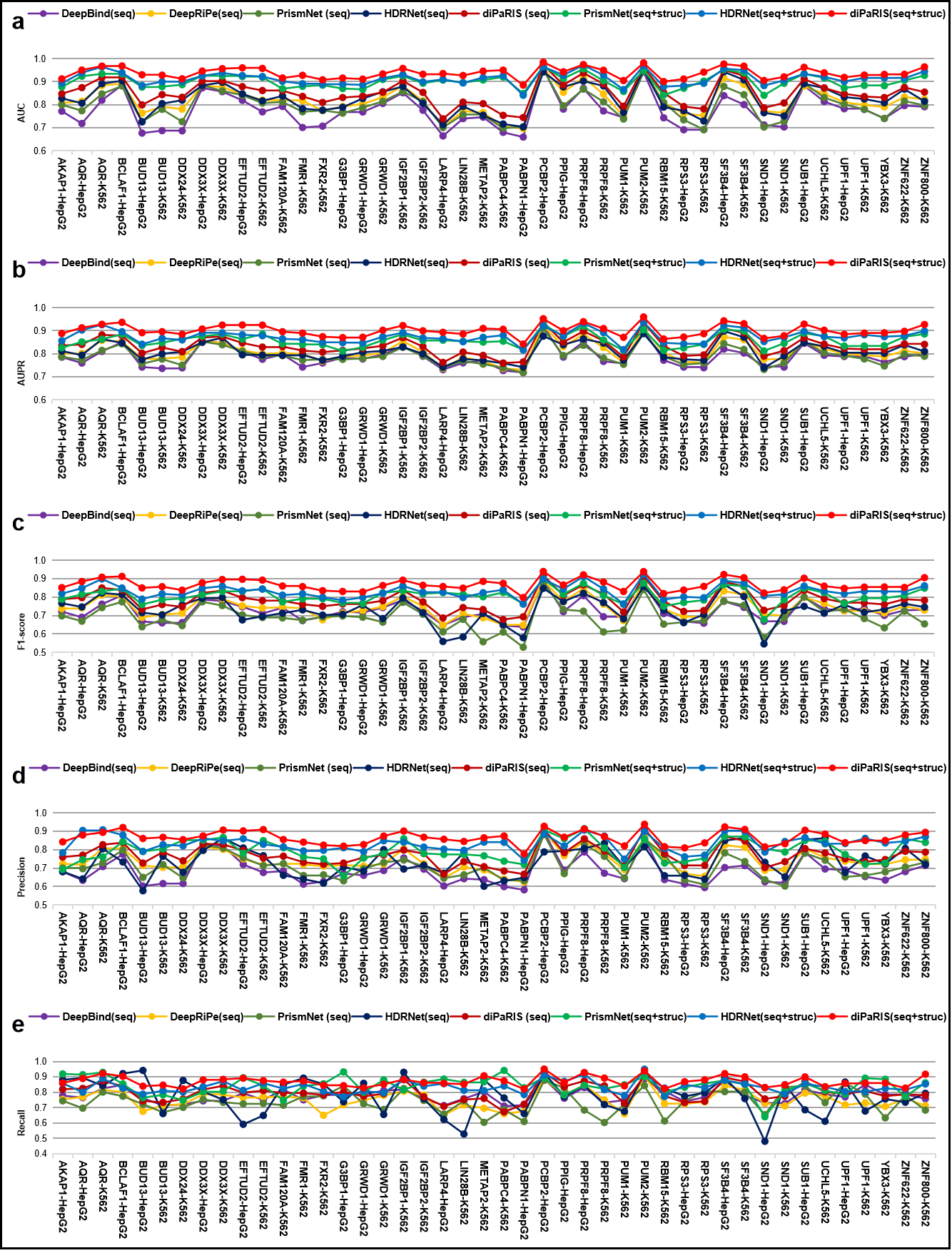


**Figure S1** Performance of comparative methods on each dataset. Sub-figures **a**, **b**, **c**, **d**, and **e** represent the performance across AUC, AUPR, F1-score, precision, and recall metrics, respectively. In these Sub-figures, (seq) means the model uses only sequence features as input, while (seq+struc) means it uses both sequence and structure features.Our method outperforms all others, achieving the highest AUC, AUPR, and F1-score across all 44 datasets, the highest precision on 38 datasets, and the highest recall on 27 datasets.


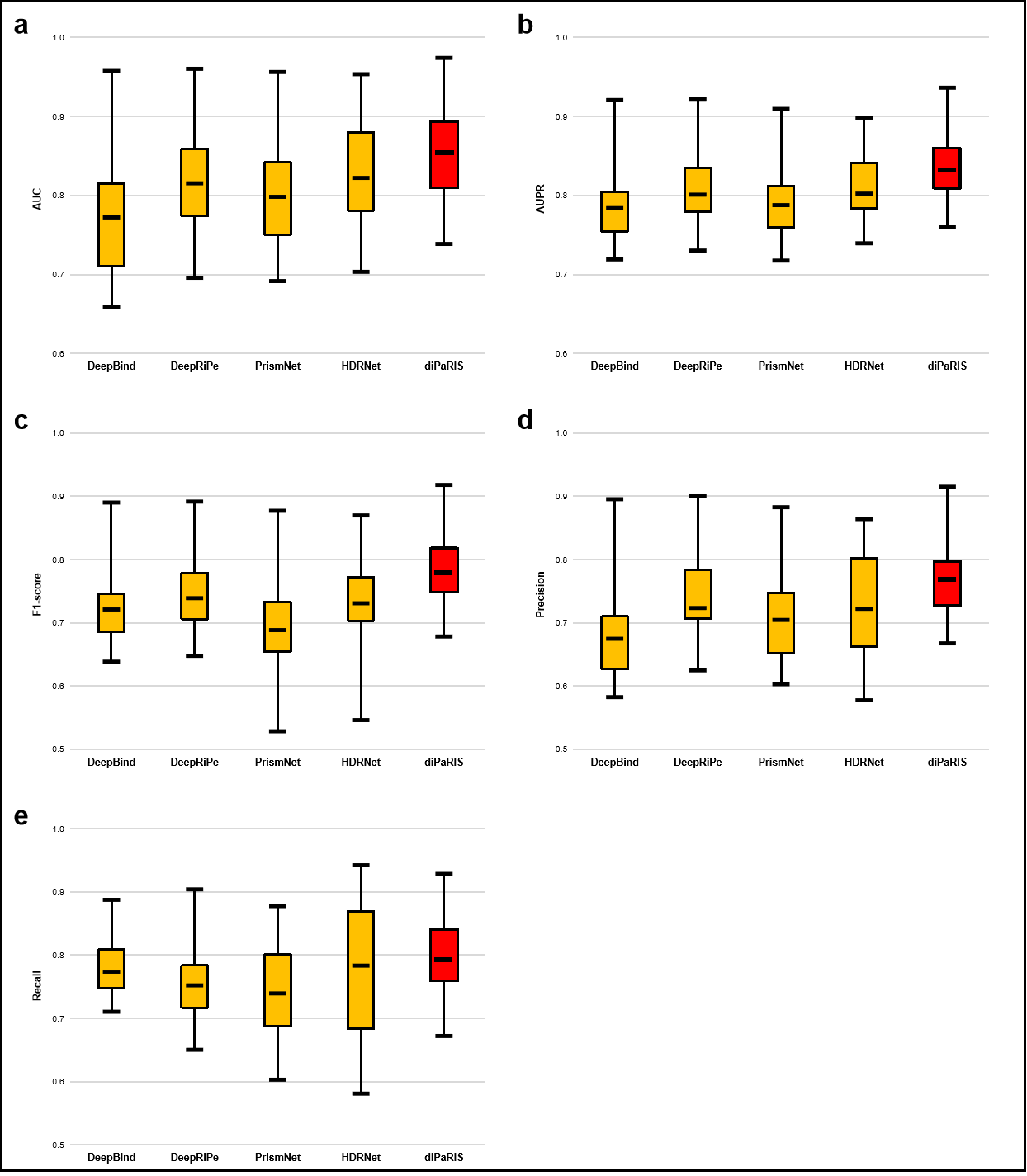


**Figure S2** Performance of comparative methods using only the one-hot encoded sequence feature as input across all datasets. Sub-figures **a**, **b**, **c**, **d**, and **e** represent the performance for AUC, AUPR, F1-score, precision, and recall metrics, respectively. The center line represents the median, while the box bounds indicate the upper and lower quartiles. diPaRIS consistently achieves the highest average scores across all metrics.

**
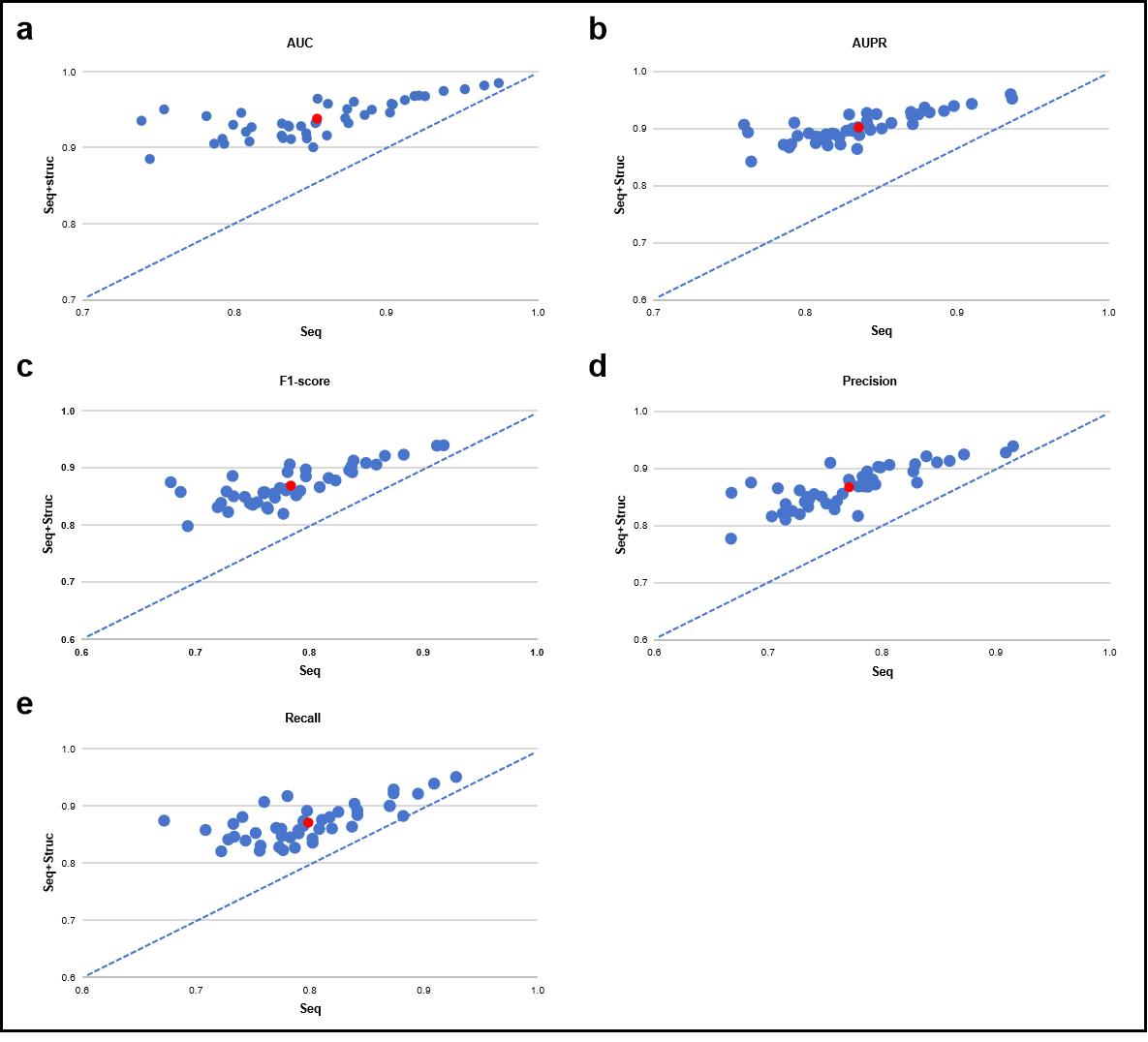
**

**Figure S3** Performance improvement for each metric on each dataset when using sequence data alone versus using both sequence and structural data. Sub-figures **a**, **b**, **c**, **d**, and **e** show the differences across the AUC, AUPR, F1-score, precision, and recall metrics, respectively. The vertical axis represents the performance when using both sequence and structural features (Seq+Struc), while the horizontal axis represents the performance when using sequence features only (Seq). Red dots in the figures indicate the average metric differences across all datasets. Across all metrics, nearly all datasets show performance improvements when incorporating structural features.

**
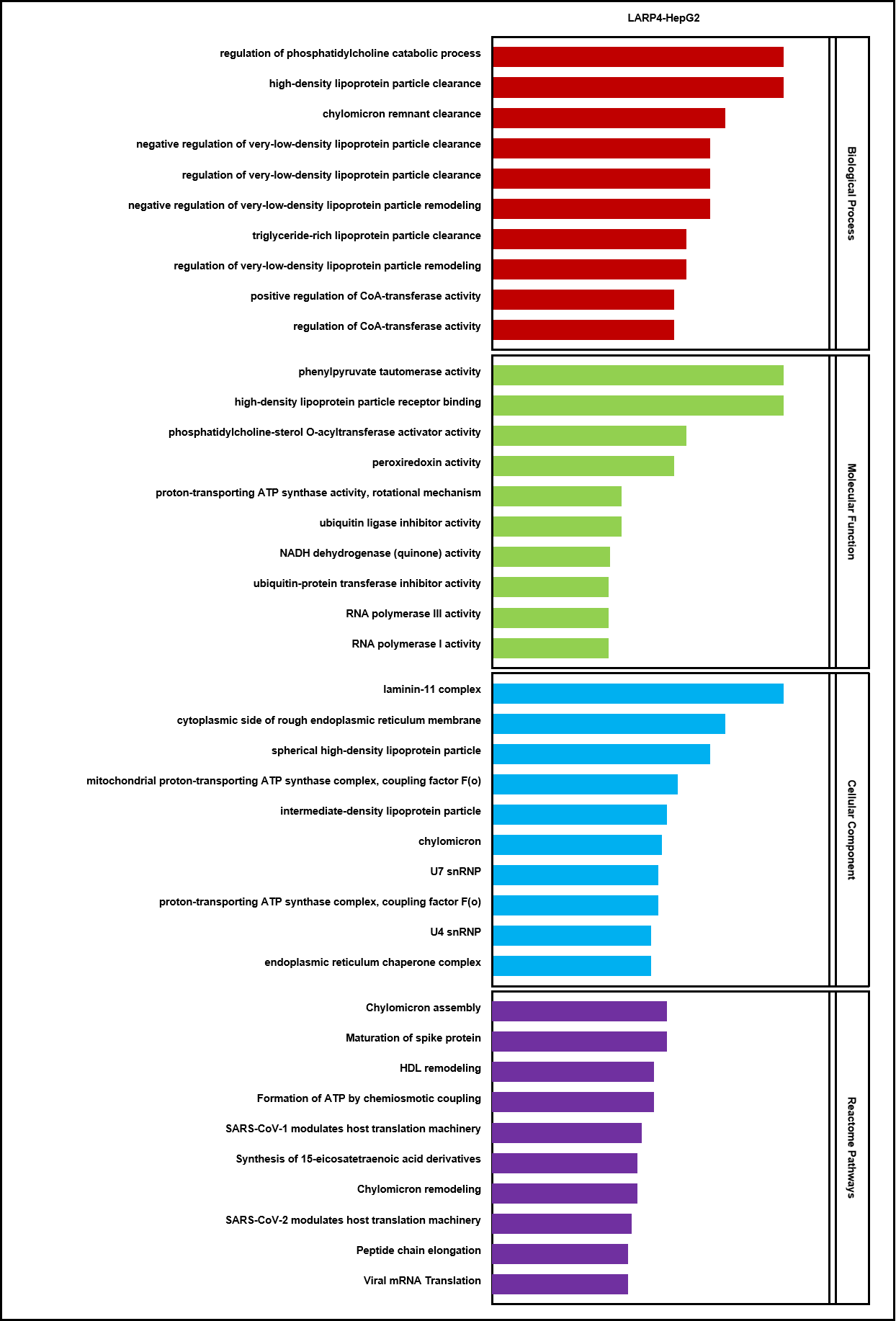
**

**Figure S4** Gene Ontology and Reactome pathway enrichment of LARP4-binding genes in the HepG2 cell line. The enrichment analysis was conducted using the PANTHER knowledgebase^[1]^. The figure displays the top 10 entries for biological processes, molecular functions, cellular components, and Reactome pathways.

**
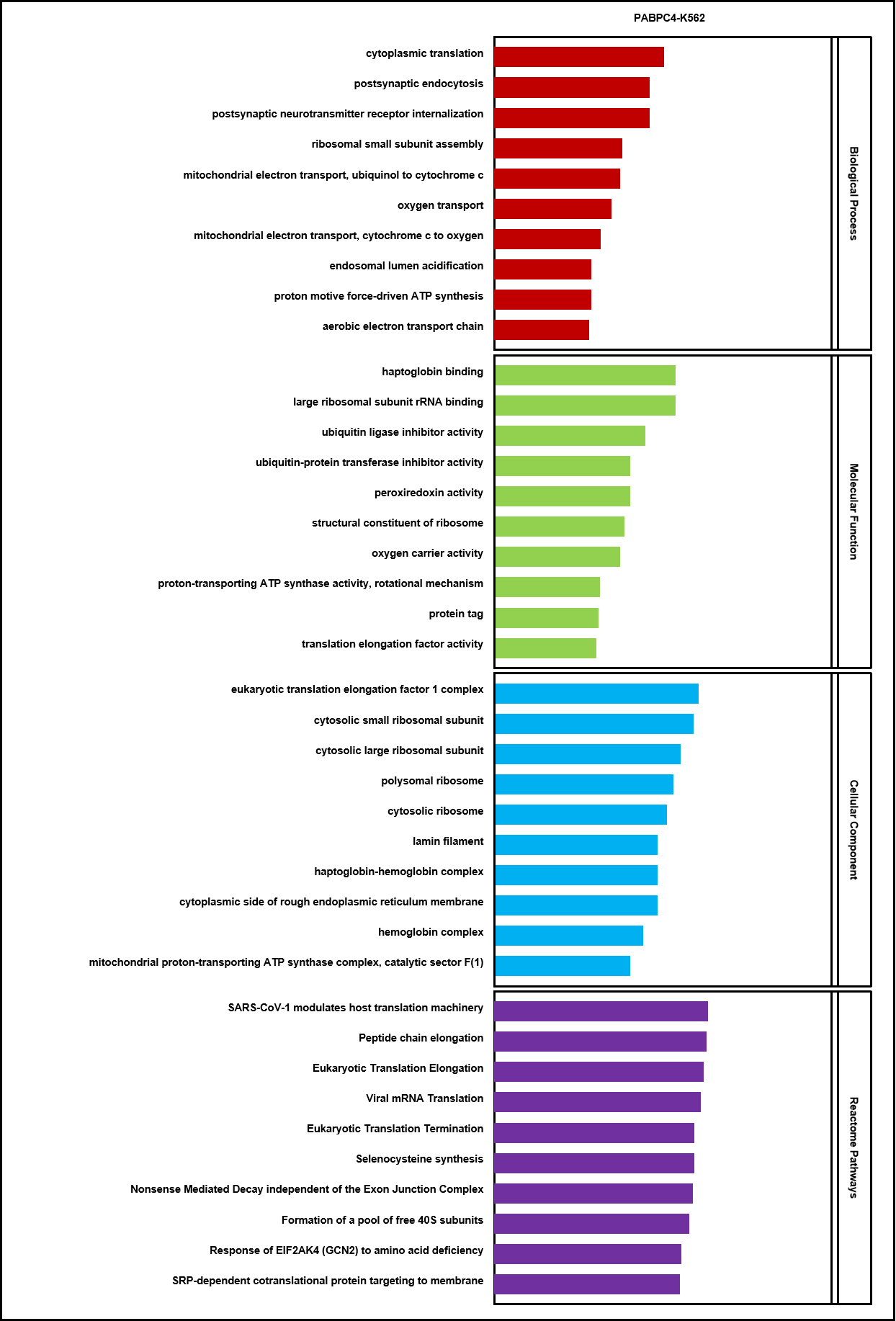
**

**Figure S5** Gene Ontology and Reactome pathway enrichment of PABPC4-binding genes in the K562 cell line. The enrichment analysis was conducted using the PANTHER knowledgebase^[1]^. The figure displays the top 10 entries for biological processes, molecular functions, cellular components, and Reactome pathways.

**
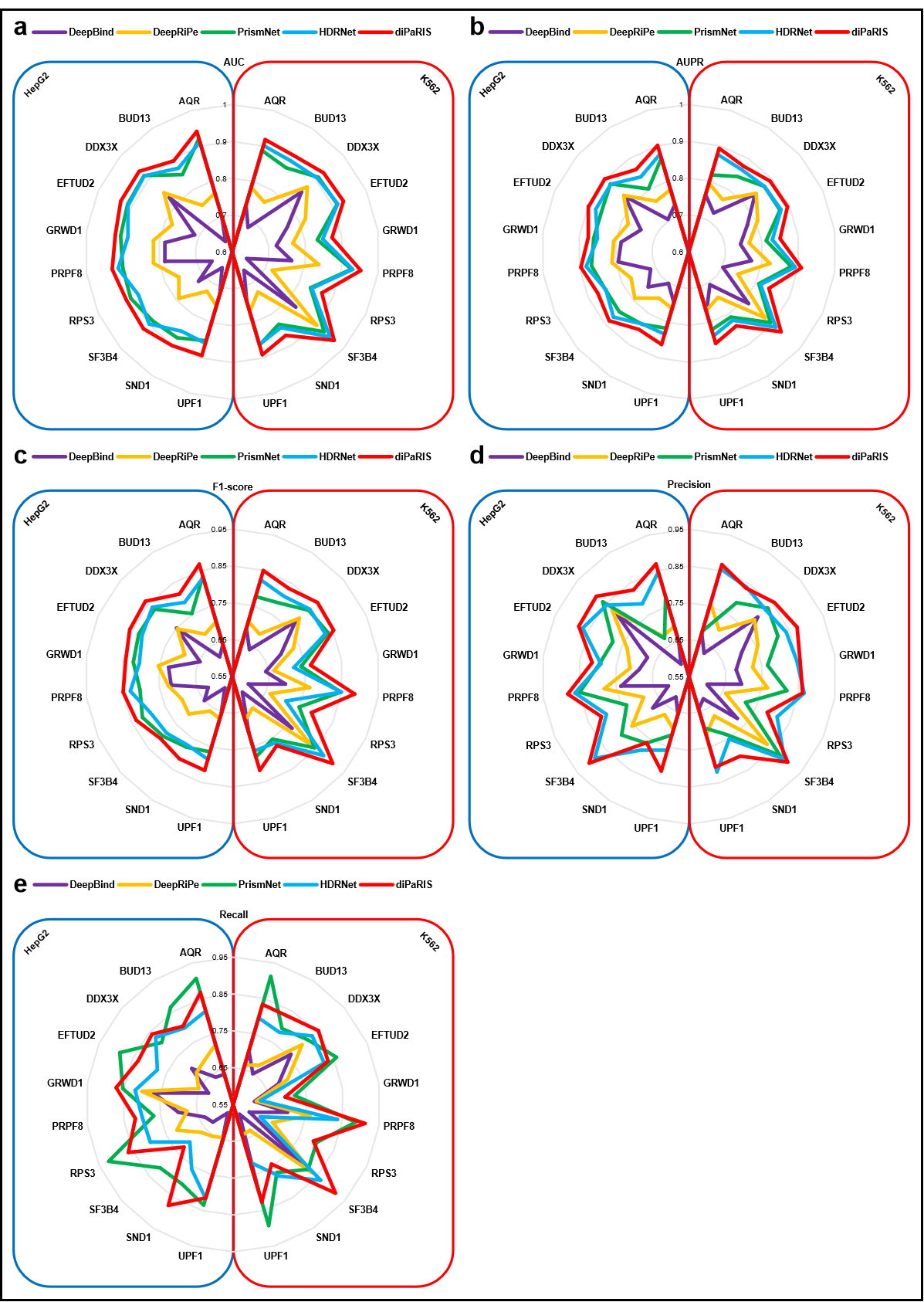
**

**Figure S6** Cross-test performance for each metric on each dataset. Sub-figures **a**, **b**, **c**, **d**, and **e** show the performance across the AUC, AUPR, F1-score, precision, and recall metrics, respectively. The left side shows the performance under the HepG2 cell line while the right side shows the performance under the K562 cell line. diPaRIS significantly outperforms state-of-the-art methods in AUC, AUPR, and F1-score.


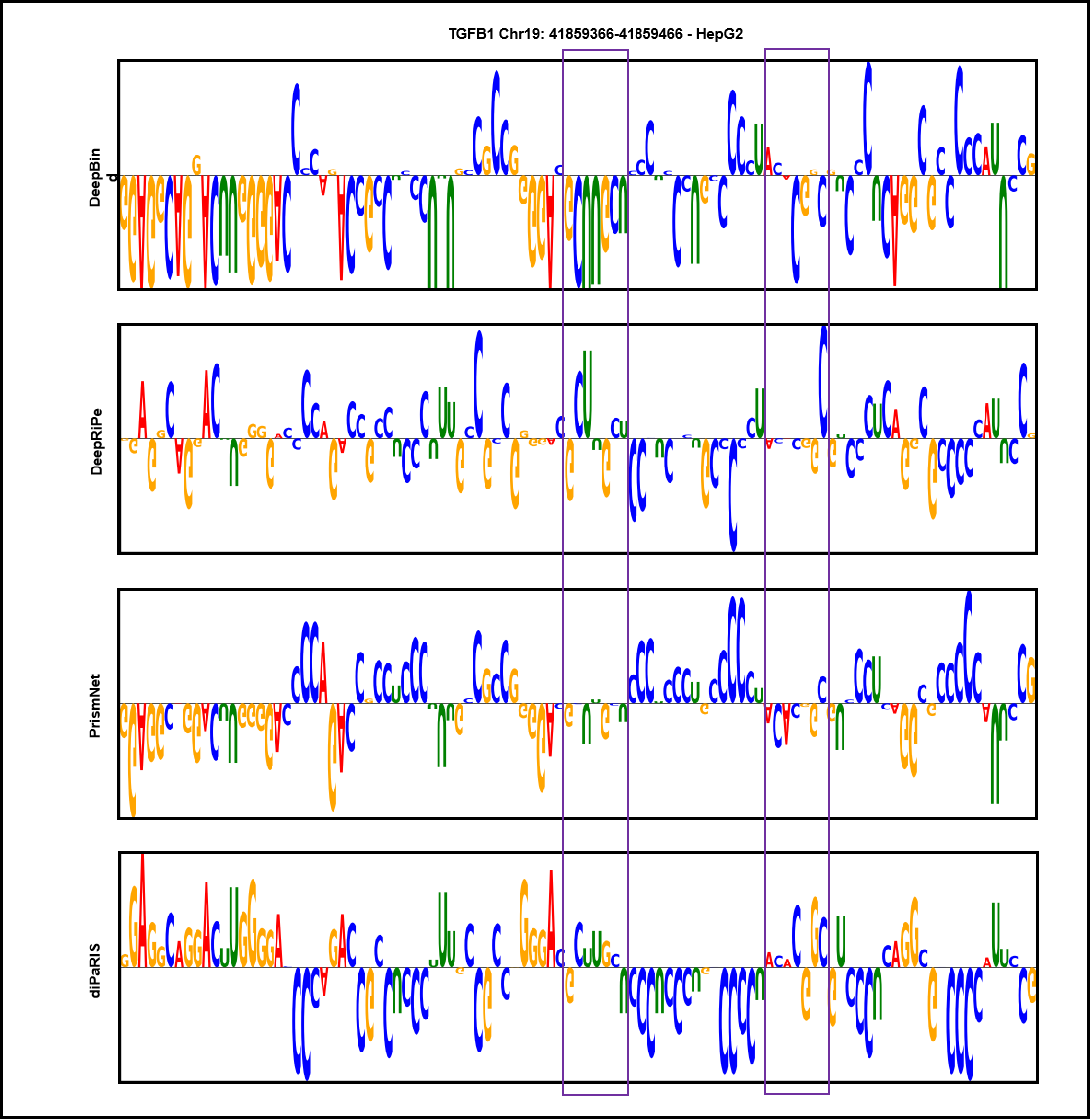


**Figure S7** Attribution maps of comparative methods for Chr19: 41859366-41859466 under the HepG2 cell line for DDX3X binding. Attribution map learned by diPaRIS shows that nucleotides with cytosine have higher weights compared to those learned by other methods. Purple boxes highlight areas where the weights predicted by diPaRIS significantly differ from those of the comparative methods. HDRNet is excluded from this analysis due to its low feature resolution.


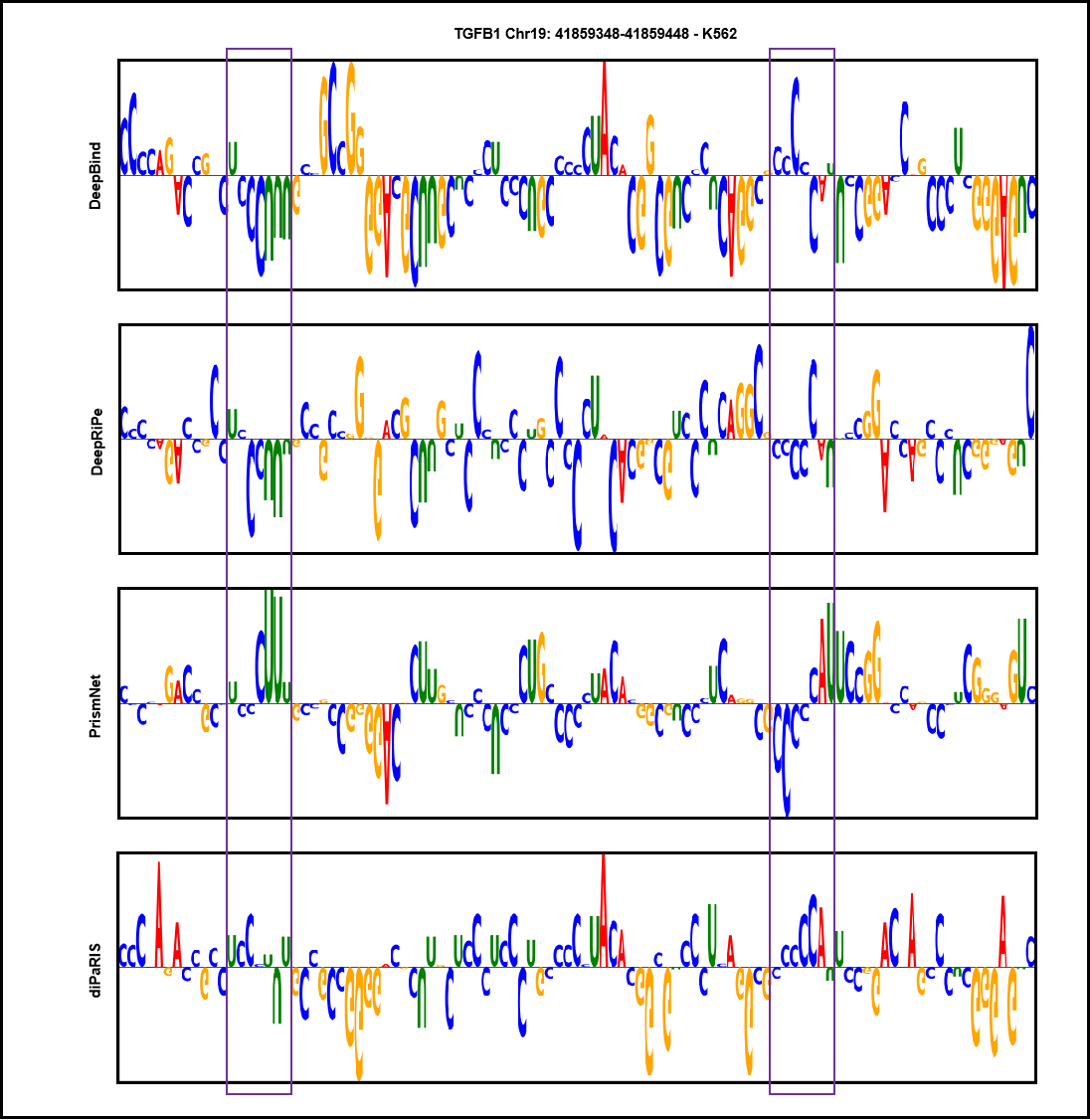


**Figure S8** Attribution maps of comparative methods for Chr19: 41859366-41859466 under the K562 cell line for DDX3X binding. Attribution map learned by diPaRIS shows that nucleotides with cytosine and uracil have lower weights compared to those learned by other methods. Purple boxes highlight areas where the weights predicted by diPaRIS significantly differ from those of the comparative methods. HDRNet is excluded from this analysis due to its low feature resolution.


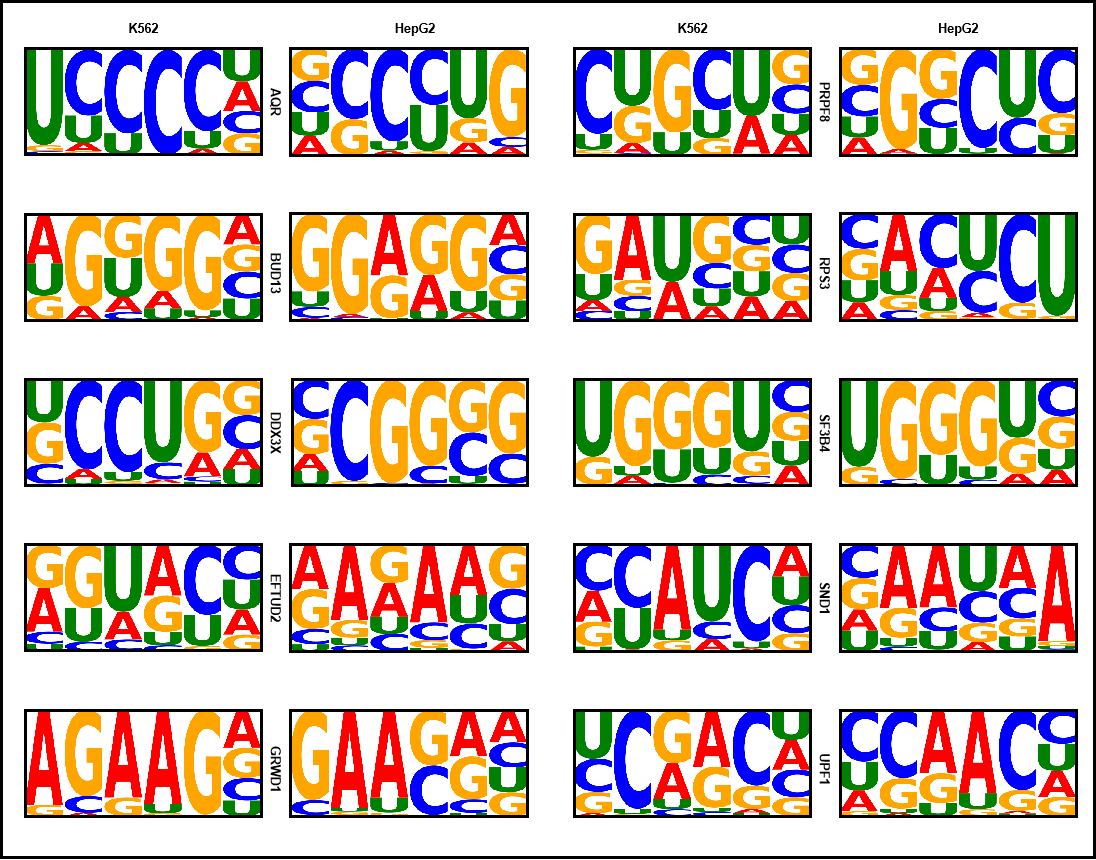


**Figure S9** Sequence motifs constructed from PWMs learned by DeepBind. The common proteins of two cell lines are labeled in the middle.


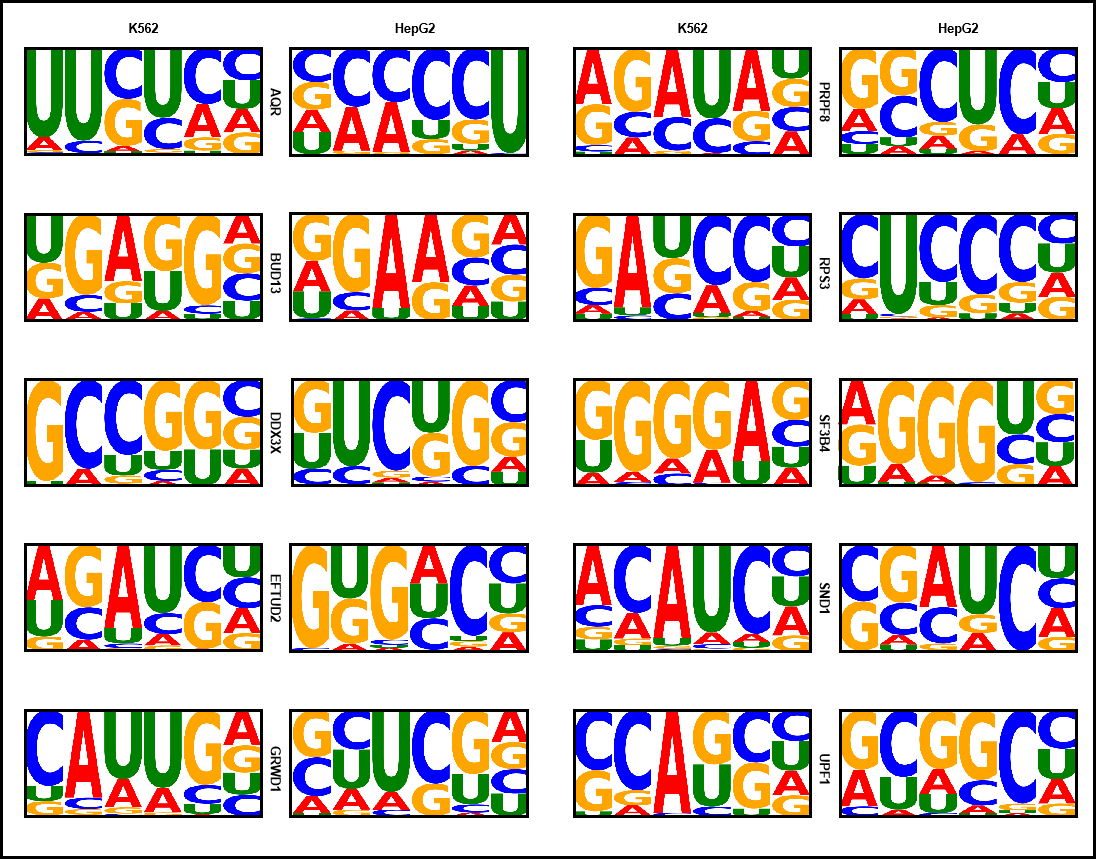


**Figure S10** Sequence motifs constructed from PWMs learned by DeepRiPe. The common proteins of two cell lines are labeled in the middle.


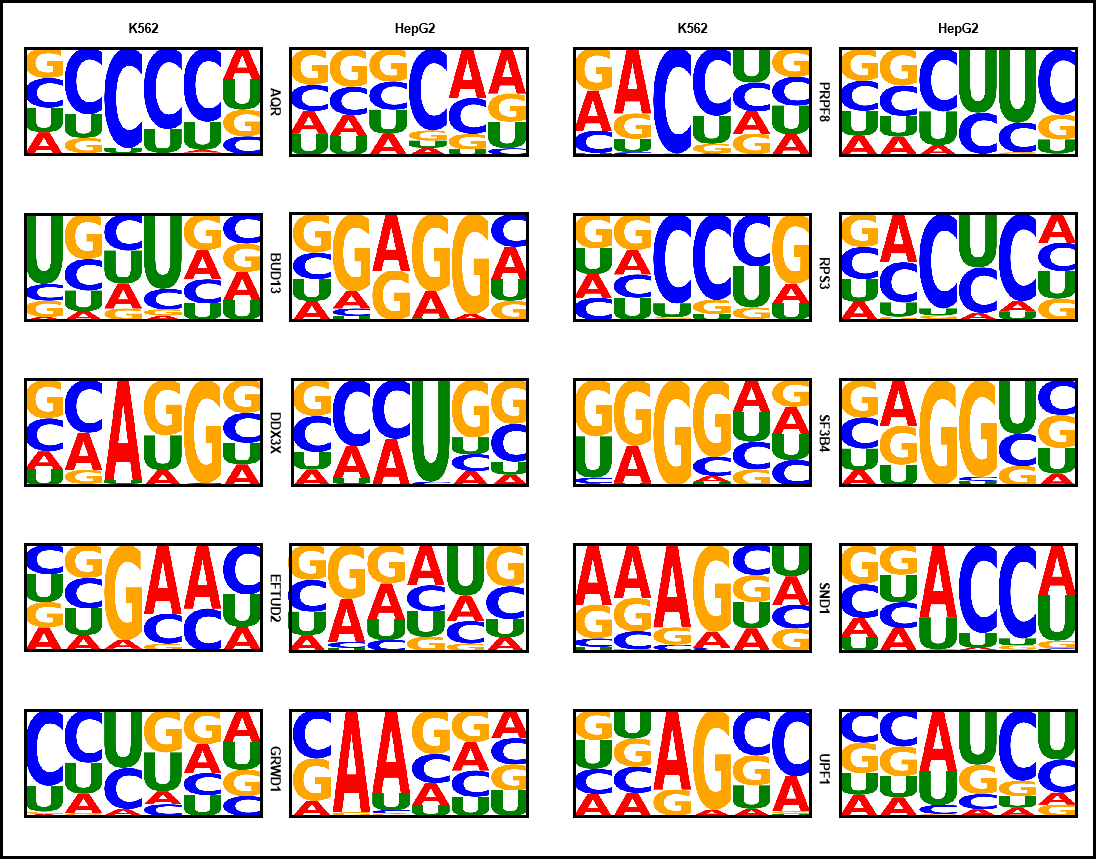


**Figure S11** Sequence motifs constructed from PWMs learned by PrismNet. The common proteins of two cell lines are labeled in the middle.


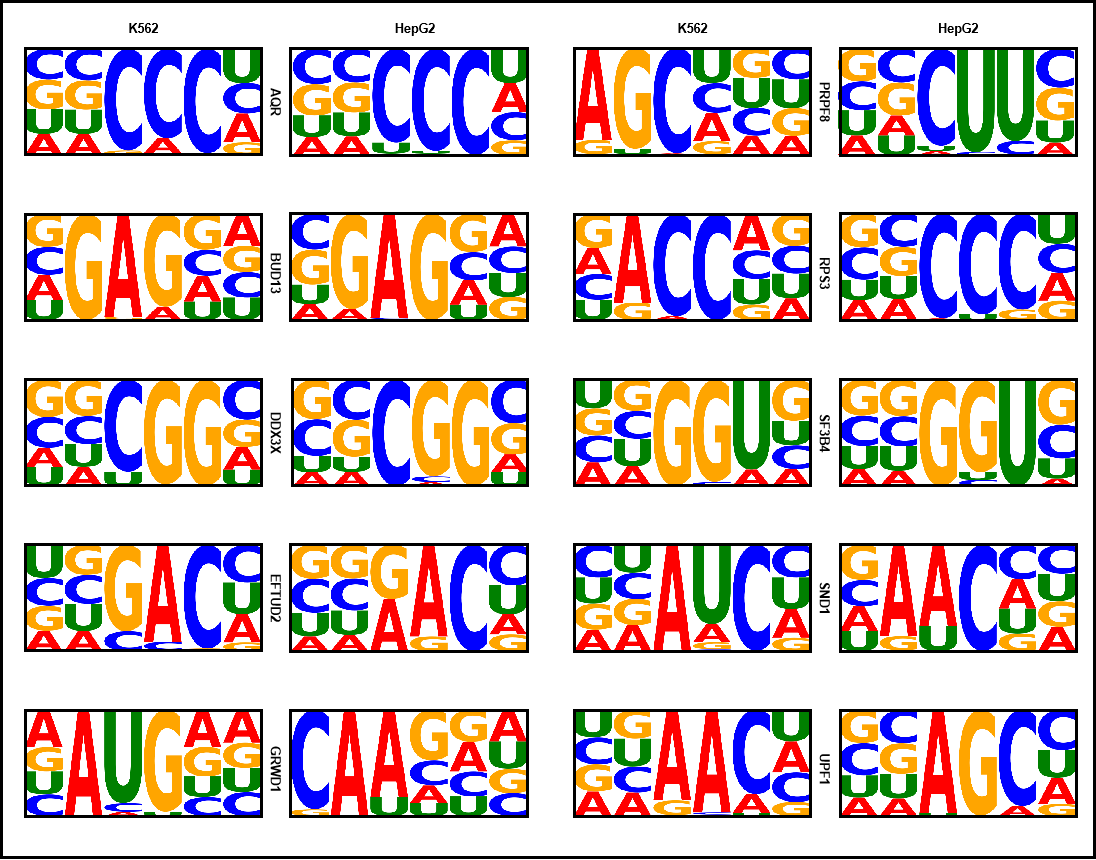


**Figure S12** Sequence motifs constructed from PWMs learned by HDRNet. The common proteins of two cell lines are labeled in the middle.


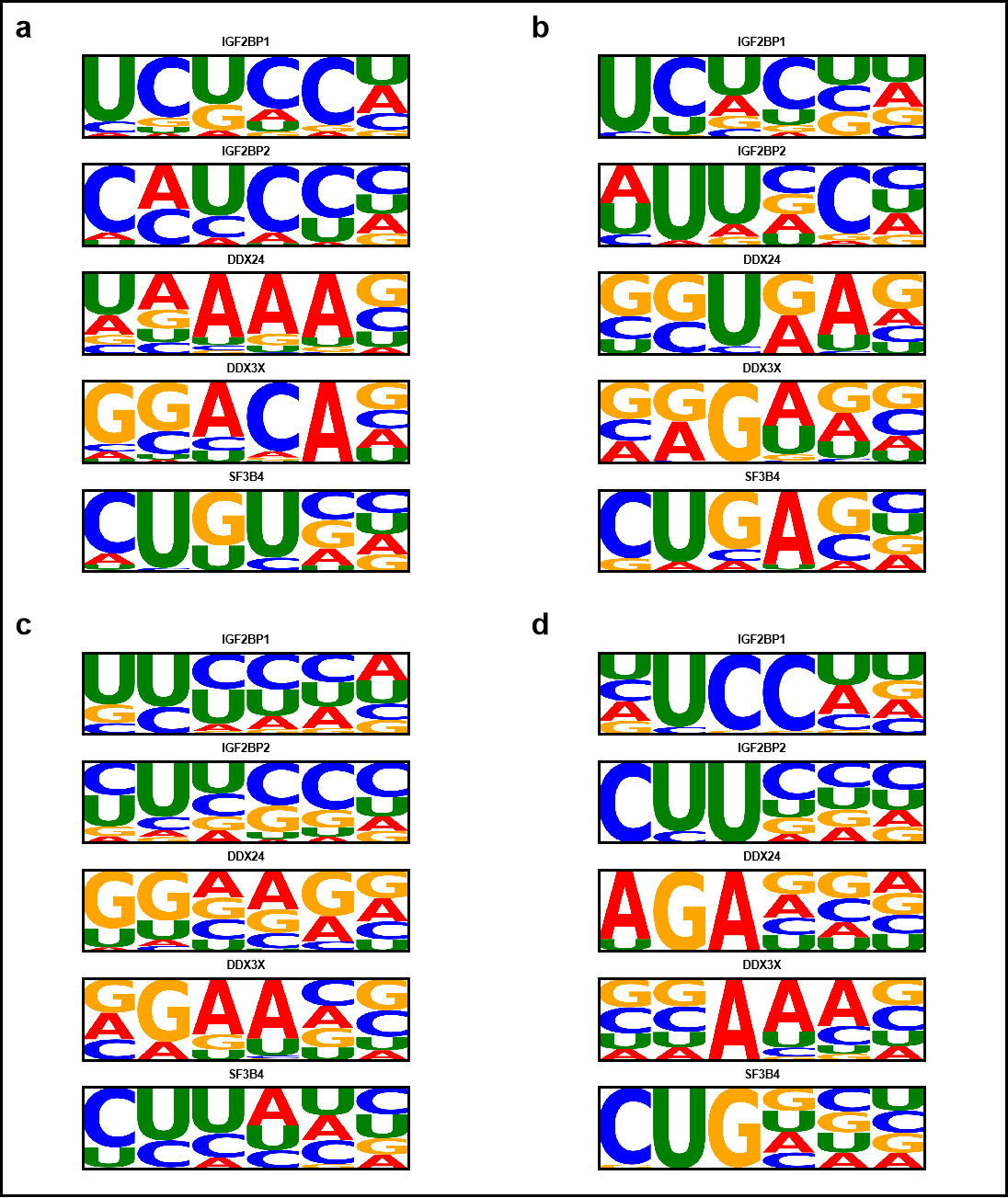


**Figure S13** Sequence motifs constructed from PWMs learned by comparative methods. Sub-figures **a**, **b**, **c**, and **d** display the sequence motifs learned by DeepBind, DeepRiPe, PrismNet, and HDRNet, respectively. For each method, the sequence motifs of proteins from the same family are more similar than those from different families.

## Supplementary Tables

**Table S1** The total number of samples for each dataset.

| Cell lines | Proteins | Sample numbers |
| --- | --- | --- |
| HepG2 | AKAP1 | 9408 |
|  | AQR | 12722 |
|  | BCLAF1 | 6870 |
|  | BUD13 | 4066 |
|  | DDX3X | 9116 |
|  | EFTUD2 | 4640 |
|  | G3BP1 | 7066 |
|  | GRWD1 | 14702 |
|  | LARP4 | 9782 |
|  | PABPN1 | 6450 |
|  | PCBP2 | 4390 |
|  | PPIG | 21556 |
|  | PRPF8 | 18826 |
|  | RPS3 | 8782 |
|  | SF3B4 | 5866 |
|  | SND1 | 8492 |
|  | SUB1 | 4048 |
|  | UPF1 | 13786 |
| K562 | AQR | 36904 |
|  | BUD13 | 10296 |
|  | DDX24 | 14122 |
|  | DDX3X | 10924 |
|  | EFTUD2 | 4290 |
|  | FAM120A | 6060 |
|  | FMR1 | 5262 |
|  | FXR2 | 2054 |
|  | GRWD1 | 6548 |
|  | IGF2BP1 | 7832 |
|  | IGF2BP2 | 6790 |
|  | LIN28B | 10240 |
|  | METAP2 | 5698 |
|  | PABPC4 | 10748 |
|  | PRPF8 | 7596 |
|  | PUM1 | 4484 |
|  | PUM2 | 5340 |
|  | RBM15 | 12266 |
|  | RPS3 | 4470 |
|  | SF3B4 | 14338 |
|  | SND1 | 6828 |
|  | UCHL5 | 22612 |
|  | UPF1 | 20592 |
|  | YBX3 | 31046 |
|  | ZNF622 | 16696 |
|  | ZNF800 | 4274 |

**Table S2** The t-test results of predicted scores between each pair of comparative methods. The table presents the p-values from these pairwise t-tests. Typically, a p-value less than 0.05 is considered statistically significant, while p < 0.0001 indicates an extremely statistically significant difference.

| Methods | Metrics | DeepRiPe | PrismNet | HDRNet | diPaRIS |
| --- | --- | --- | --- | --- | --- |
| DeepBind | AUC | < 0.0001 | < 0.0001 | < 0.0001 | < 0.0001 |
|  | ACC | < 0.0001 | < 0.0001 | < 0.0001 | < 0.0001 |
|  | Precision | < 0.0001 | < 0.0001 | < 0.0001 | < 0.0001 |
|  | Recall | < 0.0001 | < 0.0001 | < 0.0001 | < 0.0001 |
|  | F1-score | < 0.0001 | < 0.0001 | < 0.0001 | < 0.0001 |
|  | AUPR | < 0.0001 | < 0.0001 | < 0.0001 | < 0.0001 |
| DeepRiPe | AUC |  | < 0.0001 | < 0.0001 | < 0.0001 |
|  | ACC |  | < 0.0001 | < 0.0001 | < 0.0001 |
|  | Precision |  | < 0.0001 | < 0.0001 | < 0.0001 |
|  | Recall |  | < 0.0001 | < 0.0001 | < 0.0001 |
|  | F1-score |  | < 0.0001 | < 0.0001 | < 0.0001 |
|  | AUPR |  | < 0.0001 | < 0.0001 | < 0.0001 |
| PrismNet | AUC |  |  | < 0.0001 | < 0.0001 |
|  | ACC |  |  | < 0.0001 | < 0.0001 |
|  | Precision |  |  | < 0.0001 | < 0.0001 |
|  | Recall |  |  | 0.2102 | 0.0626 |
|  | F1-score |  |  | < 0.0001 | < 0.0001 |
|  | AUPR |  |  | < 0.0001 | < 0.0001 |
| HDRNet | AUC |  |  |  | < 0.0001 |
|  | ACC |  |  |  | < 0.0001 |
|  | Precision |  |  |  | 0.0245 |
|  | Recall |  |  |  | < 0.0001 |
|  | F1-score |  |  |  | < 0.0001 |
|  | AUPR |  |  |  | < 0.0001 |

**Table S3** ACC of comparative methods on each dataset. 'Without Attention' refers to the performance metrics when the attention mechanism is removed from the diPaRIS model, while 'Without icSHAPE-DS' indicates the metrics obtained by using raw data without incorporating icSHAPE-DS encoding.

| Datasets | Methods | | | | | | |
| --- | --- | --- | --- | --- | --- | --- | --- |
|  | DeepBind | DeepRiPe | PrismNet | HDRNet | diPaRIS | | |
|  |  |  |  |  |  | without Attention | without icSHAPE-DS |
| AKAP1-HepG2 | 0.6979 | 0.7308 | 0.7518 | 0.8090 | **0.8431** | 0.8137 | 0.8249 |
| AQR-HepG2 | 0.6514 | 0.7343 | 0.7874 | 0.8561 | **0.8828** | 0.8579 | 0.8644 |
| AQR-K562 | 0.7420 | 0.8096 | 0.8066 | 0.9014 | **0.9063** | 0.8901 | 0.9020 |
| BCLAF1-HepG2 | 0.7979 | 0.8058 | 0.8467 | 0.8552 | **0.9132** | 0.8706 | 0.8689 |
| BUD13-HepG2 | 0.6211 | 0.6933 | 0.7623 | 0.7924 | **0.8487** | 0.8204 | 0.8062 |
| BUD13-K562 | 0.6353 | 0.7094 | 0.7899 | 0.8169 | **0.8570** | 0.8292 | 0.8373 |
| DDX24-K562 | 0.6314 | 0.7067 | 0.8001 | 0.8175 | **0.8345** | 0.8297 | 0.8312 |
| DDX3X-HepG2 | 0.7953 | 0.7993 | 0.8161 | 0.8464 | **0.8778** | 0.8583 | 0.8563 |
| DDX3X-K562 | 0.7863 | 0.7959 | 0.8376 | 0.8626 | **0.8974** | 0.8655 | 0.8755 |
| EFTUD2-HepG2 | 0.7276 | 0.7553 | 0.8196 | 0.8397 | **0.8946** | 0.8588 | 0.8511 |
| EFTUD2-K562 | 0.6942 | 0.7376 | 0.8442 | 0.8466 | **0.8925** | 0.8653 | 0.8688 |
| FAM120A-K562 | 0.7144 | 0.7421 | 0.7821 | 0.8053 | **0.8521** | 0.8097 | 0.8256 |
| FMR1-K562 | 0.6459 | 0.7397 | 0.7805 | 0.8042 | **0.8515** | 0.8335 | 0.8278 |
| FXR2-K562 | 0.6449 | 0.6922 | 0.7764 | 0.8015 | **0.8314** | 0.8088 | 0.8104 |
| G3BP1-HepG2 | 0.6901 | 0.7165 | 0.7150 | 0.7895 | **0.8300** | 0.8076 | 0.8048 |
| GRWD1-HepG2 | 0.6887 | 0.7243 | 0.7538 | 0.7978 | **0.8259** | 0.8111 | 0.8079 |
| GRWD1-K562 | 0.7170 | 0.7485 | 0.8103 | 0.8246 | **0.8582** | 0.8292 | 0.8321 |
| IGF2BP1-K562 | 0.7734 | 0.8010 | 0.8383 | 0.8540 | **0.8898** | 0.8704 | 0.8656 |
| IGF2BP2-K562 | 0.7022 | 0.7386 | 0.8021 | 0.8162 | **0.8647** | 0.8264 | 0.8331 |
| LARP4-HepG2 | 0.6133 | 0.6451 | 0.8102 | 0.8235 | **0.8574** | 0.8574 | 0.8307 |
| LIN28B-K562 | 0.6658 | 0.7017 | 0.8000 | 0.7952 | **0.8479** | 0.8228 | 0.8479 |
| METAP2-K562 | 0.6687 | 0.6917 | 0.7912 | 0.8356 | **0.8789** | 0.8528 | 0.8374 |
| PABPC4-K562 | 0.6156 | 0.6430 | 0.8004 | 0.8409 | **0.8735** | 0.8485 | 0.8541 |
| PABPN1-HepG2 | 0.6040 | 0.6374 | 0.7420 | 0.7633 | **0.7992** | 0.7707 | 0.7766 |
| PCBP2-HepG2 | 0.8855 | 0.8900 | 0.8999 | 0.9002 | **0.9355** | 0.9062 | 0.9105 |
| PPIG-HepG2 | 0.7059 | 0.7692 | 0.8248 | 0.8402 | **0.8673** | 0.8367 | 0.8491 |
| PRPF8-HepG2 | 0.8023 | 0.8461 | 0.8766 | 0.9121 | **0.9181** | 0.9009 | 0.9102 |
| PRPF8-K562 | 0.6934 | 0.7642 | 0.8098 | 0.8479 | **0.8785** | 0.8485 | 0.8557 |
| PUM1-K562 | 0.6655 | 0.6801 | 0.7300 | 0.7657 | **0.8261** | 0.8105 | 0.8007 |
| PUM2-K562 | 0.8660 | 0.8827 | 0.8725 | 0.9212 | **0.9384** | 0.9097 | 0.9189 |
| RBM15-K562 | 0.6669 | 0.7328 | 0.7363 | 0.7900 | **0.8182** | 0.7951 | 0.8029 |
| RPS3-HepG2 | 0.6349 | 0.6854 | 0.7628 | 0.7910 | **0.8318** | 0.7864 | 0.8079 |
| RPS3-K562 | 0.6112 | 0.6816 | 0.7668 | 0.7935 | **0.8644** | 0.8306 | 0.8407 |
| SF3B4-HepG2 | 0.7524 | 0.8322 | 0.8642 | 0.8842 | **0.9209** | 0.8924 | 0.9050 |
| SF3B4-K562 | 0.7276 | 0.8104 | 0.8584 | 0.8805 | **0.9050** | 0.8851 | 0.8945 |
| SND1-HepG2 | 0.6477 | 0.6987 | 0.7196 | 0.7943 | **0.8166** | 0.7974 | 0.7945 |
| SND1-K562 | 0.6403 | 0.6932 | 0.7868 | 0.8161 | **0.8395** | 0.8092 | 0.8215 |
| SUB1-HepG2 | 0.8011 | 0.8033 | 0.8501 | 0.8598 | **0.9000** | 0.8842 | 0.8691 |
| UCHL5-K562 | 0.7226 | 0.7646 | 0.8273 | 0.8364 | **0.8623** | 0.8448 | 0.8516 |
| UPF1-HepG2 | 0.7063 | 0.7296 | 0.7665 | 0.8198 | **0.8419** | 0.8213 | 0.8297 |
| UPF1-K562 | 0.6977 | 0.7227 | 0.7660 | 0.8355 | **0.8539** | 0.8407 | 0.8382 |
| YBX3-K562 | 0.6678 | 0.7162 | 0.7717 | 0.8339 | **0.8513** | 0.8280 | 0.8391 |
| ZNF622-K562 | 0.7072 | 0.7485 | 0.8162 | 0.8310 | **0.8556** | 0.8349 | 0.8462 |
| ZNF800-K562 | 0.7150 | 0.7404 | 0.8441 | 0.8646 | **0.9032** | 0.8547 | 0.8788 |

**Table S4** AUC of comparative methods on each dataset. 'Without Attention' refers to the performance metrics when the attention mechanism is removed from the diPaRIS model, while 'Without icSHAPE-DS' indicates the metrics obtained by using raw data without incorporating icSHAPE-DS encoding.

| Datasets | Methods | | | | | | |
| --- | --- | --- | --- | --- | --- | --- | --- |
|  | DeepBind | DeepRiPe | PrismNet | HDRNet | diPaRIS | | |
|  |  |  |  |  |  | without Attention | without icSHAPE-DS |
| AKAP1-HepG2 | 0.7718 | 0.8068 | 0.8772 | 0.8932 | **0.9122** | 0.8963 | 0.9005 |
| AQR-HepG2 | 0.7183 | 0.8147 | 0.9236 | 0.9390 | **0.9506** | 0.9340 | 0.9390 |
| AQR-K562 | 0.8195 | 0.8836 | 0.9341 | 0.9644 | **0.9678** | 0.9575 | 0.9640 |
| BCLAF1-HepG2 | 0.8849 | 0.8915 | 0.9335 | 0.9388 | **0.9684** | 0.9464 | 0.9416 |
| BUD13-HepG2 | 0.6766 | 0.7632 | 0.8765 | 0.8831 | **0.9300** | 0.8980 | 0.8951 |
| BUD13-K562 | 0.6872 | 0.7916 | 0.8783 | 0.8999 | **0.9282** | 0.9062 | 0.9145 |
| DDX24-K562 | 0.6864 | 0.7804 | 0.8870 | 0.9000 | **0.9126** | 0.9086 | 0.9090 |
| DDX3X-HepG2 | 0.8729 | 0.8830 | 0.9255 | 0.9252 | **0.9460** | 0.9303 | 0.9333 |
| DDX3X-K562 | 0.8566 | 0.8763 | 0.9249 | 0.9378 | **0.9567** | 0.9390 | 0.9468 |
| EFTUD2-HepG2 | 0.8190 | 0.8458 | 0.9222 | 0.9267 | **0.9602** | 0.9294 | 0.9308 |
| EFTUD2-K562 | 0.7688 | 0.8198 | 0.9194 | 0.9224 | **0.9576** | 0.9325 | 0.9403 |
| FAM120A-K562 | 0.7925 | 0.8189 | 0.8692 | 0.9016 | **0.9160** | 0.8943 | 0.9047 |
| FMR1-K562 | 0.7009 | 0.8171 | 0.8798 | 0.8911 | **0.9277** | 0.9114 | 0.9033 |
| FXR2-K562 | 0.7068 | 0.7743 | 0.8848 | 0.8886 | **0.9081** | 0.8906 | 0.8938 |
| G3BP1-HepG2 | 0.7678 | 0.7918 | 0.8702 | 0.8892 | **0.9157** | 0.8933 | 0.8924 |
| GRWD1-HepG2 | 0.7683 | 0.8028 | 0.8666 | 0.8858 | **0.9112** | 0.8963 | 0.8913 |
| GRWD1-K562 | 0.8055 | 0.8330 | 0.9095 | 0.9155 | **0.9337** | 0.9164 | 0.9145 |
| IGF2BP1-K562 | 0.8520 | 0.8815 | 0.9244 | 0.9309 | **0.9576** | 0.9419 | 0.9377 |
| IGF2BP2-K562 | 0.7759 | 0.8160 | 0.8933 | 0.8991 | **0.9323** | 0.9090 | 0.9127 |
| LARP4-HepG2 | 0.6642 | 0.7026 | 0.9070 | 0.9101 | **0.9353** | 0.9353 | 0.9117 |
| LIN28B-K562 | 0.7396 | 0.7716 | 0.8963 | 0.8944 | **0.9269** | 0.9052 | 0.9269 |
| METAP2-K562 | 0.7458 | 0.7659 | 0.9073 | 0.9180 | **0.9457** | 0.9327 | 0.9190 |
| PABPC4-K562 | 0.6798 | 0.7063 | 0.9203 | 0.9258 | **0.9502** | 0.9258 | 0.9313 |
| PABPN1-HepG2 | 0.6593 | 0.6959 | 0.8484 | 0.8410 | **0.8851** | 0.8518 | 0.8620 |
| PCBP2-HepG2 | 0.9575 | 0.9602 | 0.9664 | 0.9652 | **0.9848** | 0.9717 | 0.9742 |
| PPIG-HepG2 | 0.7809 | 0.8535 | 0.9139 | 0.9283 | **0.9429** | 0.9212 | 0.9320 |
| PRPF8-HepG2 | 0.8708 | 0.9181 | 0.9553 | 0.9720 | **0.9744** | 0.9642 | 0.9686 |
| PRPF8-K562 | 0.7727 | 0.8463 | 0.9044 | 0.9281 | **0.9498** | 0.9253 | 0.9306 |
| PUM1-K562 | 0.7439 | 0.7494 | 0.8574 | 0.8654 | **0.9050** | 0.9000 | 0.8956 |
| PUM2-K562 | 0.9421 | 0.9524 | 0.9493 | 0.9699 | **0.9815** | 0.9666 | 0.9710 |
| RBM15-K562 | 0.7430 | 0.8086 | 0.8415 | 0.8783 | **0.9005** | 0.8740 | 0.8852 |
| RPS3-HepG2 | 0.6909 | 0.7614 | 0.8714 | 0.8827 | **0.9116** | 0.8723 | 0.8918 |
| RPS3-K562 | 0.6916 | 0.7525 | 0.9025 | 0.8930 | **0.9414** | 0.9185 | 0.9285 |
| SF3B4-HepG2 | 0.8394 | 0.9154 | 0.9525 | 0.9567 | **0.9767** | 0.9633 | 0.9643 |
| SF3B4-K562 | 0.8005 | 0.8884 | 0.9379 | 0.9504 | **0.9675** | 0.9478 | 0.9618 |
| SND1-HepG2 | 0.7119 | 0.7724 | 0.8409 | 0.8831 | **0.9052** | 0.8756 | 0.8755 |
| SND1-K562 | 0.7032 | 0.7642 | 0.8931 | 0.9057 | **0.9208** | 0.8920 | 0.9064 |
| SUB1-HepG2 | 0.8827 | 0.8808 | 0.9341 | 0.9338 | **0.9625** | 0.9537 | 0.9405 |
| UCHL5-K562 | 0.8136 | 0.8470 | 0.9165 | 0.9215 | **0.9386** | 0.9289 | 0.9287 |
| UPF1-HepG2 | 0.7823 | 0.8109 | 0.8725 | 0.9007 | **0.9188** | 0.9002 | 0.9087 |
| UPF1-K562 | 0.7795 | 0.7984 | 0.8838 | 0.9164 | **0.9289** | 0.9174 | 0.9195 |
| YBX3-K562 | 0.7399 | 0.7903 | 0.8827 | 0.9164 | **0.9317** | 0.9134 | 0.9213 |
| ZNF622-K562 | 0.7961 | 0.8330 | 0.9053 | 0.9144 | **0.9321** | 0.9135 | 0.9256 |
| ZNF800-K562 | 0.7910 | 0.8236 | 0.9269 | 0.9454 | **0.9642** | 0.9375 | 0.9521 |

**Table S5** AUPR of comparative methods on each dataset. 'Without Attention' refers to the performance metrics when the attention mechanism is removed from the diPaRIS model, while 'Without icSHAPE-DS' indicates the metrics obtained by using raw data without incorporating icSHAPE-DS encoding.

| Datasets | Methods | | | | | | |
| --- | --- | --- | --- | --- | --- | --- | --- |
|  | DeepBind | DeepRiPe | PrismNet | HDRNet | diPaRIS | | |
|  |  |  |  |  |  | without Attention | without icSHAPE-DS |
| AKAP1-HepG2 | 0.7852 | 0.8007 | 0.8262 | 0.8568 | **0.8882** | 0.8626 | 0.8632 |
| AQR-HepG2 | 0.7598 | 0.8014 | 0.8515 | 0.9030 | **0.9128** | 0.8964 | 0.9004 |
| AQR-K562 | 0.8104 | 0.8578 | 0.8632 | 0.9261 | **0.9279** | 0.9189 | 0.9269 |
| BCLAF1-HepG2 | 0.8502 | 0.8529 | 0.8847 | 0.8958 | **0.9365** | 0.9081 | 0.9006 |
| BUD13-HepG2 | 0.7423 | 0.7780 | 0.8357 | 0.8415 | **0.8914** | 0.8699 | 0.8655 |
| BUD13-K562 | 0.7364 | 0.7795 | 0.8479 | 0.8668 | **0.8959** | 0.8693 | 0.8750 |
| DDX24-K562 | 0.7390 | 0.7848 | 0.8648 | 0.8601 | **0.8847** | 0.8692 | 0.8765 |
| DDX3X-HepG2 | 0.8546 | 0.8553 | 0.8761 | 0.8909 | **0.9074** | 0.8897 | 0.8942 |
| DDX3X-K562 | 0.8470 | 0.8463 | 0.8849 | 0.8907 | **0.9245** | 0.8967 | 0.9101 |
| EFTUD2-HepG2 | 0.8088 | 0.8153 | 0.8635 | 0.8826 | **0.9249** | 0.8938 | 0.8846 |
| EFTUD2-K562 | 0.7762 | 0.7995 | 0.8857 | 0.8783 | **0.9243** | 0.8937 | 0.9003 |
| FAM120A-K562 | 0.7969 | 0.8034 | 0.8432 | 0.8648 | **0.8958** | 0.8543 | 0.8725 |
| FMR1-K562 | 0.7423 | 0.7991 | 0.8403 | 0.8614 | **0.8896** | 0.8764 | 0.8817 |
| FXR2-K562 | 0.7600 | 0.7679 | 0.8389 | 0.8495 | **0.8742** | 0.8610 | 0.8584 |
| G3BP1-HepG2 | 0.7829 | 0.7874 | 0.8122 | 0.8481 | **0.8701** | 0.8543 | 0.8538 |
| GRWD1-HepG2 | 0.7772 | 0.7919 | 0.8282 | 0.8465 | **0.8717** | 0.8518 | 0.8612 |
| GRWD1-K562 | 0.7968 | 0.8040 | 0.8591 | 0.8738 | **0.9021** | 0.8777 | 0.8734 |
| IGF2BP1-K562 | 0.8324 | 0.8545 | 0.8839 | 0.8916 | **0.9224** | 0.8992 | 0.8993 |
| IGF2BP2-K562 | 0.7892 | 0.8080 | 0.8572 | 0.8688 | **0.8995** | 0.8706 | 0.8677 |
| LARP4-HepG2 | 0.7309 | 0.7365 | 0.8589 | 0.8653 | **0.8930** | 0.8930 | 0.8786 |
| LIN28B-K562 | 0.7606 | 0.7839 | 0.8542 | 0.8522 | **0.8857** | 0.8634 | 0.8857 |
| METAP2-K562 | 0.7698 | 0.7683 | 0.8505 | 0.8711 | **0.9100** | 0.8819 | 0.8805 |
| PABPC4-K562 | 0.7270 | 0.7360 | 0.8545 | 0.8802 | **0.9063** | 0.8859 | 0.8946 |
| PABPN1-HepG2 | 0.7191 | 0.7303 | 0.8176 | 0.8162 | **0.8420** | 0.8305 | 0.8373 |
| PCBP2-HepG2 | 0.9207 | 0.9222 | 0.9275 | 0.9206 | **0.9520** | 0.9278 | 0.9269 |
| PPIG-HepG2 | 0.7855 | 0.8312 | 0.8752 | 0.8778 | **0.8998** | 0.8806 | 0.8818 |
| PRPF8-HepG2 | 0.8517 | 0.8852 | 0.9197 | 0.9323 | **0.9391** | 0.9274 | 0.9320 |
| PRPF8-K562 | 0.7667 | 0.8271 | 0.8594 | 0.8919 | **0.9094** | 0.8904 | 0.8949 |
| PUM1-K562 | 0.7583 | 0.7611 | 0.8144 | 0.8177 | **0.8714** | 0.8542 | 0.8610 |
| PUM2-K562 | 0.9025 | 0.9108 | 0.9044 | 0.9372 | **0.9597** | 0.9318 | 0.9369 |
| RBM15-K562 | 0.7719 | 0.8046 | 0.8150 | 0.8474 | **0.8640** | 0.8429 | 0.8486 |
| RPS3-HepG2 | 0.7423 | 0.7654 | 0.8255 | 0.8431 | **0.8724** | 0.8452 | 0.8491 |
| RPS3-K562 | 0.7393 | 0.7610 | 0.8407 | 0.8417 | **0.8869** | 0.8727 | 0.8816 |
| SF3B4-HepG2 | 0.8203 | 0.8728 | 0.9057 | 0.9230 | **0.9429** | 0.9186 | 0.9289 |
| SF3B4-K562 | 0.8032 | 0.8610 | 0.8984 | 0.9133 | **0.9305** | 0.9159 | 0.9191 |
| SND1-HepG2 | 0.7444 | 0.7762 | 0.8123 | 0.8529 | **0.8666** | 0.8443 | 0.8412 |
| SND1-K562 | 0.7421 | 0.7745 | 0.8455 | 0.8708 | **0.8779** | 0.8571 | 0.8645 |
| SUB1-HepG2 | 0.8457 | 0.8504 | 0.8890 | 0.8970 | **0.9287** | 0.9112 | 0.9052 |
| UCHL5-K562 | 0.7935 | 0.8221 | 0.8760 | 0.8741 | **0.9021** | 0.8880 | 0.8884 |
| UPF1-HepG2 | 0.7881 | 0.7987 | 0.8335 | 0.8687 | **0.8845** | 0.8663 | 0.8744 |
| UPF1-K562 | 0.7893 | 0.7940 | 0.8339 | 0.8824 | **0.8898** | 0.8777 | 0.8789 |
| YBX3-K562 | 0.7634 | 0.7877 | 0.8352 | 0.8740 | **0.8910** | 0.8718 | 0.8758 |
| ZNF622-K562 | 0.7872 | 0.8127 | 0.8736 | 0.8780 | **0.8973** | 0.8764 | 0.8813 |
| ZNF800-K562 | 0.7982 | 0.8014 | 0.8857 | 0.8986 | **0.9269** | 0.8966 | 0.9059 |

**Table S6** F1-score of comparative methods on each dataset. 'Without Attention' refers to the performance metrics when the attention mechanism is removed from the diPaRIS model, while 'Without icSHAPE-DS' indicates the metrics obtained by using raw data without incorporating icSHAPE-DS encoding.

| Datasets | Methods | | | | | | |
| --- | --- | --- | --- | --- | --- | --- | --- |
|  | DeepBind | DeepRiPe | PrismNet | HDRNet | diPaRIS | | |
|  |  |  |  |  |  | without Attention | without icSHAPE-DS |
| AKAP1-HepG2 | 0.7211 | 0.7378 | 0.7882 | 0.8187 | **0.8515** | 0.8216 | 0.8261 |
| AQR-HepG2 | 0.6910 | 0.7416 | 0.8150 | 0.8483 | **0.8847** | 0.8598 | 0.8673 |
| AQR-K562 | 0.7617 | 0.8111 | 0.8310 | 0.8985 | **0.9081** | 0.8936 | 0.9023 |
| BCLAF1-HepG2 | 0.8097 | 0.8031 | 0.8457 | 0.8496 | **0.9123** | 0.8711 | 0.8653 |
| BUD13-HepG2 | 0.6676 | 0.6898 | 0.7599 | 0.7891 | **0.8498** | 0.8208 | 0.8098 |
| BUD13-K562 | 0.6601 | 0.7058 | 0.7861 | 0.8174 | **0.8567** | 0.8200 | 0.8282 |
| DDX24-K562 | 0.6619 | 0.7125 | 0.7923 | 0.8112 | **0.8378** | 0.8199 | 0.8270 |
| DDX3X-HepG2 | 0.7864 | 0.7989 | 0.8136 | 0.8480 | **0.8776** | 0.8552 | 0.8573 |
| DDX3X-K562 | 0.7788 | 0.7898 | 0.8311 | 0.8594 | **0.8954** | 0.8637 | 0.8744 |
| EFTUD2-HepG2 | 0.7475 | 0.7527 | 0.8315 | 0.8342 | **0.8968** | 0.8626 | 0.8498 |
| EFTUD2-K562 | 0.7108 | 0.7409 | 0.8446 | 0.8434 | **0.8922** | 0.8573 | 0.8637 |
| FAM120A-K562 | 0.7425 | 0.7450 | 0.7742 | 0.8107 | **0.8602** | 0.8134 | 0.8312 |
| FMR1-K562 | 0.6726 | 0.7398 | 0.7912 | 0.8183 | **0.8569** | 0.8351 | 0.8379 |
| FXR2-K562 | 0.6927 | 0.6769 | 0.7908 | 0.7997 | **0.8350** | 0.8170 | 0.8104 |
| G3BP1-HepG2 | 0.7208 | 0.7140 | 0.7687 | 0.7830 | **0.8305** | 0.7986 | 0.7977 |
| GRWD1-HepG2 | 0.7161 | 0.7265 | 0.7650 | 0.7982 | **0.8280** | 0.8033 | 0.8108 |
| GRWD1-K562 | 0.7428 | 0.7479 | 0.8233 | 0.8207 | **0.8629** | 0.8356 | 0.8302 |
| IGF2BP1-K562 | 0.7839 | 0.8039 | 0.8341 | 0.8605 | **0.8919** | 0.8643 | 0.8693 |
| IGF2BP2-K562 | 0.7247 | 0.7506 | 0.8181 | 0.8263 | **0.8642** | 0.8298 | 0.8250 |
| LARP4-HepG2 | 0.6479 | 0.6476 | 0.8242 | 0.8278 | **0.8574** | 0.8574 | 0.8395 |
| LIN28B-K562 | 0.6911 | 0.7098 | 0.8155 | 0.7975 | **0.8487** | 0.8200 | 0.8487 |
| METAP2-K562 | 0.7083 | 0.6880 | 0.8018 | 0.8240 | **0.8854** | 0.8529 | 0.8458 |
| PABPC4-K562 | 0.6461 | 0.6494 | 0.8252 | 0.8397 | **0.8744** | 0.8535 | 0.8575 |
| PABPN1-HepG2 | 0.6384 | 0.6477 | 0.7639 | 0.7618 | **0.7976** | 0.7783 | 0.7769 |
| PCBP2-HepG2 | 0.8900 | 0.8915 | 0.8986 | 0.8983 | **0.9390** | 0.9074 | 0.9066 |
| PPIG-HepG2 | 0.7212 | 0.7750 | 0.8154 | 0.8439 | **0.8659** | 0.8388 | 0.8453 |
| PRPF8-HepG2 | 0.8082 | 0.8462 | 0.8744 | 0.9114 | **0.9207** | 0.9027 | 0.9104 |
| PRPF8-K562 | 0.6935 | 0.7620 | 0.8087 | 0.8443 | **0.8818** | 0.8475 | 0.8591 |
| PUM1-K562 | 0.6899 | 0.6724 | 0.7613 | 0.7631 | **0.8307** | 0.8063 | 0.8029 |
| PUM2-K562 | 0.8716 | 0.8858 | 0.8762 | 0.9238 | **0.9385** | 0.9112 | 0.9194 |
| RBM15-K562 | 0.7111 | 0.7340 | 0.7505 | 0.7871 | **0.8192** | 0.7925 | 0.8013 |
| RPS3-HepG2 | 0.6678 | 0.6932 | 0.7709 | 0.8014 | **0.8382** | 0.7877 | 0.8043 |
| RPS3-K562 | 0.6586 | 0.6932 | 0.7839 | 0.7967 | **0.8582** | 0.8337 | 0.8406 |
| SF3B4-HepG2 | 0.7778 | 0.8330 | 0.8682 | 0.8891 | **0.9226** | 0.8935 | 0.9040 |
| SF3B4-K562 | 0.7445 | 0.8127 | 0.8592 | 0.8758 | **0.9053** | 0.8810 | 0.8927 |
| SND1-HepG2 | 0.6688 | 0.7056 | 0.6780 | 0.8071 | **0.8223** | 0.7939 | 0.7926 |
| SND1-K562 | 0.6684 | 0.6997 | 0.7851 | 0.8145 | **0.8394** | 0.8095 | 0.8185 |
| SUB1-HepG2 | 0.7987 | 0.7990 | 0.8487 | 0.8596 | **0.9025** | 0.8833 | 0.8685 |
| UCHL5-K562 | 0.7362 | 0.7653 | 0.8236 | 0.8328 | **0.8596** | 0.8467 | 0.8482 |
| UPF1-HepG2 | 0.7263 | 0.7259 | 0.7659 | 0.8181 | **0.8475** | 0.8246 | 0.8325 |
| UPF1-K562 | 0.7381 | 0.7253 | 0.7941 | 0.8316 | **0.8544** | 0.8398 | 0.8377 |
| YBX3-K562 | 0.7009 | 0.7136 | 0.7943 | 0.8304 | **0.8551** | 0.8292 | 0.8369 |
| ZNF622-K562 | 0.7281 | 0.7512 | 0.8089 | 0.8301 | **0.8525** | 0.8293 | 0.8380 |
| ZNF800-K562 | 0.7321 | 0.7294 | 0.8485 | 0.8612 | **0.9056** | 0.8568 | 0.8748 |

**Table S7** Precision of comparative methods on each dataset. 'Without Attention' refers to the performance metrics when the attention mechanism is removed from the diPaRIS model, while 'Without icSHAPE-DS' indicates the metrics obtained by using raw data without incorporating icSHAPE-DS encoding.

| Datasets | Methods | | | | | | |
| --- | --- | --- | --- | --- | --- | --- | --- |
|  | DeepBind | DeepRiPe | PrismNet | HDRNet | diPaRIS | | |
|  |  |  |  |  |  | without Attention | without icSHAPE-DS |
| AKAP1-HepG2 | 0.6804 | 0.7238 | 0.6929 | 0.7843 | **0.8429** | 0.8027 | 0.8016 |
| AQR-HepG2 | 0.6342 | 0.7186 | 0.7460 | **0.9051** | 0.8801 | 0.8634 | 0.8644 |
| AQR-K562 | 0.7086 | 0.8077 | 0.7613 | **0.9086** | 0.8946 | 0.8858 | 0.9025 |
| BCLAF1-HepG2 | 0.7751 | 0.8058 | 0.8443 | 0.8797 | **0.9211** | 0.8860 | 0.8736 |
| BUD13-HepG2 | 0.6028 | 0.7144 | 0.7911 | 0.7907 | **0.8613** | 0.8331 | 0.8306 |
| BUD13-K562 | 0.6163 | 0.7040 | 0.8045 | 0.8274 | **0.8678** | 0.8409 | 0.8479 |
| DDX24-K562 | 0.6166 | 0.7073 | 0.8520 | 0.8215 | **0.8549** | 0.8410 | 0.8504 |
| DDX3X-HepG2 | 0.8355 | 0.8157 | 0.8523 | 0.8620 | **0.8750** | 0.8515 | 0.8605 |
| DDX3X-K562 | 0.8195 | 0.8022 | 0.8670 | 0.8479 | **0.9073** | 0.8611 | 0.8895 |
| EFTUD2-HepG2 | 0.7200 | 0.7501 | 0.7805 | 0.8598 | **0.9026** | 0.8476 | 0.8409 |
| EFTUD2-K562 | 0.6765 | 0.7172 | 0.8495 | 0.8300 | **0.9097** | 0.8659 | 0.8752 |
| FAM120A-K562 | 0.6843 | 0.7236 | 0.8113 | 0.8155 | **0.8555** | 0.7894 | 0.8233 |
| FMR1-K562 | 0.6130 | 0.7199 | 0.7612 | 0.7936 | **0.8413** | 0.8335 | 0.8396 |
| FXR2-K562 | 0.6268 | 0.7110 | 0.7503 | 0.7971 | **0.8252** | 0.8044 | 0.8106 |
| G3BP1-HepG2 | 0.6709 | 0.7163 | 0.6582 | 0.8112 | 0.8197 | **0.8208** | 0.8187 |
| GRWD1-HepG2 | 0.6613 | 0.7120 | 0.7550 | 0.7901 | **0.8283** | 0.8074 | 0.8175 |
| GRWD1-K562 | 0.6873 | 0.7229 | 0.7784 | 0.8490 | **0.8744** | 0.8327 | 0.8337 |
| IGF2BP1-K562 | 0.7553 | 0.8044 | 0.8599 | 0.8410 | **0.9016** | 0.8730 | 0.8565 |
| IGF2BP2-K562 | 0.6948 | 0.7243 | 0.7801 | 0.8145 | **0.8682** | 0.8202 | 0.8275 |
| LARP4-HepG2 | 0.6033 | 0.6465 | 0.7746 | 0.8032 | **0.8571** | 0.8571 | 0.8286 |
| LIN28B-K562 | 0.6428 | 0.7060 | 0.7750 | 0.7971 | **0.8451** | 0.8168 | 0.8451 |
| METAP2-K562 | 0.6376 | 0.6903 | 0.7671 | 0.8413 | **0.8650** | 0.8235 | 0.8253 |
| PABPC4-K562 | 0.5997 | 0.6410 | 0.7380 | 0.8404 | **0.8750** | 0.8342 | 0.8592 |
| PABPN1-HepG2 | 0.5823 | 0.6247 | 0.7202 | 0.7433 | 0.7769 | 0.7609 | **0.7902** |
| PCBP2-HepG2 | 0.8953 | 0.9003 | 0.9072 | 0.8871 | **0.9276** | 0.8958 | 0.8984 |
| PPIG-HepG2 | 0.6898 | 0.7686 | 0.8602 | 0.8207 | **0.8683** | 0.8417 | 0.8404 |
| PRPF8-HepG2 | 0.7879 | 0.8473 | **0.9147** | 0.9072 | 0.9129 | 0.9025 | 0.9074 |
| PRPF8-K562 | 0.6726 | 0.7776 | 0.8090 | **0.8744** | 0.8718 | 0.8639 | 0.8580 |
| PUM1-K562 | 0.6431 | 0.6915 | 0.7038 | 0.7492 | 0.8210 | 0.8072 | **0.8275** |
| PUM2-K562 | 0.8596 | 0.8691 | 0.8575 | 0.8992 | **0.9386** | 0.9041 | 0.9089 |
| RBM15-K562 | 0.6381 | 0.7440 | 0.7279 | 0.8078 | **0.8166** | 0.7894 | 0.7943 |
| RPS3-HepG2 | 0.6145 | 0.6702 | 0.7360 | 0.7619 | **0.8103** | 0.7993 | 0.7928 |
| RPS3-K562 | 0.5946 | 0.6562 | 0.7532 | 0.7706 | 0.8374 | 0.8215 | **0.8435** |
| SF3B4-HepG2 | 0.7038 | 0.8251 | 0.8717 | 0.9033 | **0.9242** | 0.8872 | 0.9078 |
| SF3B4-K562 | 0.7140 | 0.8130 | 0.8700 | 0.9005 | **0.9104** | 0.9018 | 0.8923 |
| SND1-HepG2 | 0.6261 | 0.6887 | 0.8058 | 0.7877 | **0.8158** | 0.7926 | 0.7821 |
| SND1-K562 | 0.6207 | 0.6921 | 0.7877 | **0.8446** | 0.8327 | 0.8088 | 0.8226 |
| SUB1-HepG2 | 0.7861 | 0.8053 | 0.8512 | 0.8658 | **0.9060** | 0.8788 | 0.8813 |
| UCHL5-K562 | 0.6956 | 0.7574 | 0.8465 | 0.8329 | **0.8855** | 0.8545 | 0.8585 |
| UPF1-HepG2 | 0.6898 | 0.7385 | 0.7742 | 0.8339 | **0.8384** | 0.8135 | 0.8295 |
| UPF1-K562 | 0.6541 | 0.7223 | 0.7215 | **0.8617** | 0.8499 | 0.8324 | 0.8399 |
| YBX3-K562 | 0.6352 | 0.7214 | 0.7277 | 0.8366 | **0.8505** | 0.8278 | 0.8318 |
| ZNF622-K562 | 0.6814 | 0.7457 | 0.8597 | 0.8465 | **0.8804** | 0.8483 | 0.8529 |
| ZNF800-K562 | 0.7146 | 0.7489 | 0.8398 | 0.8716 | **0.8946** | 0.8672 | 0.8775 |

**Table S8** Recall of comparative methods on each dataset. 'Without Attention' refers to the performance metrics when the attention mechanism is removed from the diPaRIS model, while 'Without icSHAPE-DS' indicates the metrics obtained by using raw data without incorporating icSHAPE-DS encoding.

| Datasets | Methods | | | | | | |
| --- | --- | --- | --- | --- | --- | --- | --- |
|  | DeepBind | DeepRiPe | PrismNet | HDRNet | diPaRIS | | |
|  |  |  |  |  |  | without Attention | without icSHAPE-DS |
| AKAP1-HepG2 | 0.7777 | 0.7534 | **0.9195** | 0.8579 | 0.8606 | 0.8419 | 0.8532 |
| AQR-HepG2 | 0.7647 | 0.7691 | **0.9143** | 0.8000 | 0.8895 | 0.8563 | 0.8705 |
| AQR-K562 | 0.8243 | 0.8152 | **0.9296** | 0.8891 | 0.9220 | 0.9015 | 0.9022 |
| BCLAF1-HepG2 | 0.8484 | 0.8022 | 0.8540 | 0.8249 | **0.9038** | 0.8573 | 0.8575 |
| BUD13-HepG2 | 0.7604 | 0.6754 | 0.7643 | 0.7875 | **0.8395** | 0.8102 | 0.7920 |
| BUD13-K562 | 0.7147 | 0.7150 | 0.7833 | 0.8100 | **0.8463** | 0.8012 | 0.8102 |
| DDX24-K562 | 0.7204 | 0.7214 | 0.7482 | 0.8016 | **0.8216** | 0.8009 | 0.8049 |
| DDX3X-HepG2 | 0.7439 | 0.7863 | 0.8026 | 0.8353 | **0.8803** | 0.8592 | 0.8545 |
| DDX3X-K562 | 0.7460 | 0.7837 | 0.8069 | 0.8716 | **0.8841** | 0.8669 | 0.8601 |
| EFTUD2-HepG2 | 0.7928 | 0.7651 | **0.8942** | 0.8121 | 0.8914 | 0.8791 | 0.8593 |
| EFTUD2-K562 | 0.7507 | 0.7691 | 0.8432 | 0.8587 | **0.8757** | 0.8499 | 0.8539 |
| FAM120A-K562 | 0.8174 | 0.7711 | 0.7478 | 0.8234 | **0.8653** | 0.8405 | 0.8397 |
| FMR1-K562 | 0.7506 | 0.7632 | 0.8397 | 0.8538 | **0.8735** | 0.8374 | 0.8371 |
| FXR2-K562 | 0.7802 | 0.6501 | **0.8550** | 0.8061 | 0.8451 | 0.8304 | 0.8123 |
| G3BP1-HepG2 | 0.7851 | 0.7182 | **0.9323** | 0.7703 | 0.8427 | 0.7798 | 0.7803 |
| GRWD1-HepG2 | 0.7858 | 0.7476 | 0.7996 | 0.8078 | **0.8280** | 0.7996 | 0.8051 |
| GRWD1-K562 | 0.8106 | 0.7793 | **0.8802** | 0.7952 | 0.8521 | 0.8395 | 0.8274 |
| IGF2BP1-K562 | 0.8172 | 0.8060 | 0.8153 | 0.8818 | 0.8825 | 0.8559 | **0.8827** |
| IGF2BP2-K562 | 0.7597 | 0.7816 | **0.8647** | 0.8395 | 0.8615 | 0.8409 | 0.8237 |
| LARP4-HepG2 | 0.7133 | 0.6519 | **0.8860** | 0.8558 | 0.8579 | 0.8579 | 0.8509 |
| LIN28B-K562 | 0.7582 | 0.7178 | **0.8639** | 0.8126 | 0.8527 | 0.8237 | 0.8527 |
| METAP2-K562 | 0.8025 | 0.6957 | 0.8683 | 0.8107 | **0.9071** | 0.8847 | 0.8676 |
| PABPC4-K562 | 0.7102 | 0.6605 | **0.9419** | 0.8410 | 0.8742 | 0.8738 | 0.8564 |
| PABPN1-HepG2 | 0.7162 | 0.6752 | **0.8290** | 0.7846 | 0.8206 | 0.7979 | 0.7644 |
| PCBP2-HepG2 | 0.8872 | 0.8854 | 0.8960 | 0.9111 | **0.9507** | 0.9194 | 0.9151 |
| PPIG-HepG2 | 0.7612 | 0.7836 | 0.7819 | **0.8707** | 0.8637 | 0.8364 | 0.8511 |
| PRPF8-HepG2 | 0.8304 | 0.8458 | 0.8448 | 0.9160 | **0.9287** | 0.9030 | 0.9134 |
| PRPF8-K562 | 0.7329 | 0.7505 | 0.8222 | 0.8167 | **0.8922** | 0.8317 | 0.8605 |
| PUM1-K562 | 0.7479 | 0.6599 | **0.8486** | 0.7784 | 0.8413 | 0.8064 | 0.7799 |
| PUM2-K562 | 0.8869 | 0.9038 | 0.9013 | **0.9501** | 0.9390 | 0.9186 | 0.9303 |
| RBM15-K562 | 0.8078 | 0.7257 | 0.8015 | 0.7715 | **0.8226** | 0.7964 | 0.8088 |
| RPS3-HepG2 | 0.7398 | 0.7236 | 0.8326 | 0.8492 | **0.8686** | 0.7775 | 0.8167 |
| RPS3-K562 | 0.7678 | 0.7374 | 0.8570 | 0.8293 | **0.8806** | 0.8473 | 0.8388 |
| SF3B4-HepG2 | 0.8743 | 0.8420 | 0.8764 | 0.8781 | **0.9213** | 0.9000 | 0.9007 |
| SF3B4-K562 | 0.7805 | 0.8158 | 0.8510 | 0.8536 | **0.9004** | 0.8616 | 0.8938 |
| SND1-HepG2 | 0.7294 | 0.7290 | 0.6401 | 0.8295 | **0.8306** | 0.7959 | 0.8051 |
| SND1-K562 | 0.7268 | 0.7112 | 0.8094 | 0.7883 | **0.8473** | 0.8108 | 0.8151 |
| SUB1-HepG2 | 0.8164 | 0.7945 | 0.8568 | 0.8555 | **0.8998** | 0.8885 | 0.8567 |
| UCHL5-K562 | 0.7862 | 0.7755 | 0.8102 | 0.8346 | 0.8358 | **0.8394** | 0.8383 |
| UPF1-HepG2 | 0.7695 | 0.7173 | 0.7863 | 0.8049 | **0.8575** | 0.8374 | 0.8367 |
| UPF1-K562 | 0.8485 | 0.7305 | **0.8920** | 0.8038 | 0.8597 | 0.8481 | 0.8358 |
| YBX3-K562 | 0.7847 | 0.7076 | **0.8846** | 0.8251 | 0.8600 | 0.8309 | 0.8425 |
| ZNF622-K562 | 0.7862 | 0.7588 | 0.7717 | 0.8165 | **0.8268** | 0.8113 | 0.8259 |
| ZNF800-K562 | 0.7574 | 0.7126 | 0.8612 | 0.8537 | **0.9173** | 0.8478 | 0.8723 |

**Table S9** ACC of comparative methods on each dataset using only the one-hot encoding sequence feature.

| Datasets | Methods | | | | |
| --- | --- | --- | --- | --- | --- |
|  | DeepBind | DeepRiPe | PrismNet | HDRNet | diPaRIS |
| AKAP1-HepG2 | 0.6979 | 0.7308 | 0.6876 | 0.7271 | **0.7772** |
| AQR-HepG2 | 0.6514 | 0.7343 | 0.6746 | 0.6978 | **0.7945** |
| AQR-K562 | 0.7420 | 0.8096 | 0.7340 | 0.8199 | **0.8463** |
| BCLAF1-HepG2 | 0.7979 | 0.8058 | 0.7791 | 0.7850 | **0.8413** |
| BUD13-HepG2 | 0.6211 | 0.6933 | 0.6284 | 0.6113 | **0.7191** |
| BUD13-K562 | 0.6353 | 0.7094 | 0.6926 | 0.7170 | **0.7675** |
| DDX24-K562 | 0.6314 | 0.7067 | 0.6349 | 0.7093 | **0.7451** |
| DDX3X-HepG2 | 0.7953 | 0.7993 | 0.7811 | 0.7910 | **0.8201** |
| DDX3X-K562 | 0.7863 | 0.7959 | 0.7598 | 0.8111 | **0.8351** |
| EFTUD2-HepG2 | 0.7276 | 0.7553 | 0.7074 | 0.7442 | **0.7998** |
| EFTUD2-K562 | 0.6942 | 0.7376 | 0.6900 | 0.7242 | **0.7776** |
| FAM120A-K562 | 0.7144 | 0.7421 | 0.6951 | 0.6517 | **0.7757** |
| FMR1-K562 | 0.6459 | 0.7397 | 0.6690 | 0.6496 | **0.7551** |
| FXR2-K562 | 0.6449 | 0.6922 | 0.6769 | 0.6484 | **0.7359** |
| G3BP1-HepG2 | 0.6901 | 0.7165 | 0.6493 | 0.6979 | **0.7485** |
| GRWD1-HepG2 | 0.6887 | 0.7243 | 0.6899 | 0.7235 | **0.7584** |
| GRWD1-K562 | 0.7170 | 0.7485 | 0.6888 | 0.7359 | **0.7731** |
| IGF2BP1-K562 | 0.7734 | 0.8010 | 0.7573 | 0.7718 | **0.8273** |
| IGF2BP2-K562 | 0.7022 | 0.7386 | 0.7092 | 0.7046 | **0.7797** |
| LARP4-HepG2 | 0.6133 | 0.6451 | 0.6099 | 0.6049 | **0.6713** |
| LIN28B-K562 | 0.6658 | 0.7017 | 0.6623 | 0.6534 | **0.7354** |
| METAP2-K562 | 0.6687 | 0.6917 | 0.6182 | 0.6447 | **0.7291** |
| PABPC4-K562 | 0.6156 | 0.6430 | 0.5955 | 0.6147 | **0.6840** |
| PABPN1-HepG2 | 0.6040 | 0.6374 | 0.5877 | 0.6212 | **0.6798** |
| PCBP2-HepG2 | 0.8855 | 0.8900 | 0.8824 | 0.8214 | **0.9173** |
| PPIG-HepG2 | 0.7059 | 0.7692 | 0.7017 | 0.7818 | **0.8059** |
| PRPF8-HepG2 | 0.8023 | 0.8461 | 0.7563 | 0.8160 | **0.8660** |
| PRPF8-K562 | 0.6934 | 0.7642 | 0.6771 | 0.7908 | **0.8146** |
| PUM1-K562 | 0.6655 | 0.6801 | 0.5916 | 0.6861 | **0.7273** |
| PUM2-K562 | 0.8660 | 0.8827 | 0.8494 | 0.8485 | **0.9101** |
| RBM15-K562 | 0.6669 | 0.7328 | 0.7049 | 0.7004 | **0.7748** |
| RPS3-HepG2 | 0.6349 | 0.6854 | 0.6474 | 0.6539 | **0.7187** |
| RPS3-K562 | 0.6112 | 0.6816 | 0.6060 | 0.6528 | **0.7130** |
| SF3B4-HepG2 | 0.7524 | 0.8322 | 0.7726 | 0.8658 | **0.8813** |
| SF3B4-K562 | 0.7276 | 0.8104 | 0.7417 | 0.8158 | **0.8579** |
| SND1-HepG2 | 0.6477 | 0.6987 | 0.6027 | 0.6456 | **0.7246** |
| SND1-K562 | 0.6403 | 0.6932 | 0.6266 | 0.6669 | **0.7376** |
| SUB1-HepG2 | 0.8011 | 0.8033 | 0.7857 | 0.7788 | **0.8363** |
| UCHL5-K562 | 0.7226 | 0.7646 | 0.7285 | 0.7613 | **0.7907** |
| UPF1-HepG2 | 0.7063 | 0.7296 | 0.6851 | 0.7303 | **0.7656** |
| UPF1-K562 | 0.6977 | 0.7227 | 0.6602 | 0.7327 | **0.7557** |
| YBX3-K562 | 0.6678 | 0.7162 | 0.6588 | 0.7238 | **0.7531** |
| ZNF622-K562 | 0.7072 | 0.7485 | 0.7067 | 0.7722 | **0.7877** |
| ZNF800-K562 | 0.7150 | 0.7404 | 0.6606 | 0.7214 | **0.7754** |

**Table S10** AUC of comparative methods on each dataset using only the one-hot encoding sequence feature.

| Datasets | Methods | | | | |
| --- | --- | --- | --- | --- | --- |
|  | DeepBind | DeepRiPe | PrismNet | HDRNet | diPaRIS |
| AKAP1-HepG2 | 0.7718 | 0.8068 | 0.7995 | 0.8276 | **0.8476** |
| AQR-HepG2 | 0.7183 | 0.8147 | 0.7736 | 0.8063 | **0.8742** |
| AQR-K562 | 0.8195 | 0.8836 | 0.8468 | 0.8912 | **0.9185** |
| BCLAF1-HepG2 | 0.8849 | 0.8915 | 0.8815 | 0.9041 | **0.9214** |
| BUD13-HepG2 | 0.6766 | 0.7632 | 0.7266 | 0.7254 | **0.7991** |
| BUD13-K562 | 0.6872 | 0.7916 | 0.7798 | 0.8045 | **0.8438** |
| DDX24-K562 | 0.6864 | 0.7804 | 0.7261 | 0.8196 | **0.8320** |
| DDX3X-HepG2 | 0.8729 | 0.8830 | 0.8846 | 0.8838 | **0.9024** |
| DDX3X-K562 | 0.8566 | 0.8763 | 0.8600 | 0.8970 | **0.9042** |
| EFTUD2-HepG2 | 0.8190 | 0.8458 | 0.8412 | 0.8471 | **0.8786** |
| EFTUD2-K562 | 0.7688 | 0.8198 | 0.8070 | 0.8167 | **0.8615** |
| FAM120A-K562 | 0.7925 | 0.8189 | 0.8119 | 0.8380 | **0.8608** |
| FMR1-K562 | 0.7009 | 0.8171 | 0.7692 | 0.7824 | **0.8359** |
| FXR2-K562 | 0.7068 | 0.7743 | 0.7751 | 0.7763 | **0.8098** |
| G3BP1-HepG2 | 0.7678 | 0.7918 | 0.7632 | 0.7888 | **0.8310** |
| GRWD1-HepG2 | 0.7683 | 0.8028 | 0.7873 | 0.8268 | **0.8372** |
| GRWD1-K562 | 0.8055 | 0.8330 | 0.8143 | **0.8559** | 0.8539 |
| IGF2BP1-K562 | 0.8520 | 0.8815 | 0.8569 | 0.8792 | **0.9034** |
| IGF2BP2-K562 | 0.7759 | 0.8160 | 0.8136 | 0.8054 | **0.8533** |
| LARP4-HepG2 | 0.6642 | 0.7026 | 0.7006 | 0.7124 | **0.7387** |
| LIN28B-K562 | 0.7396 | 0.7716 | 0.7572 | 0.7927 | **0.8112** |
| METAP2-K562 | 0.7458 | 0.7659 | 0.7541 | 0.7535 | **0.8044** |
| PABPC4-K562 | 0.6798 | 0.7063 | 0.6980 | 0.7159 | **0.7536** |
| PABPN1-HepG2 | 0.6593 | 0.6959 | 0.7032 | 0.7034 | **0.7442** |
| PCBP2-HepG2 | 0.9575 | 0.9602 | 0.9562 | 0.9423 | **0.9740** |
| PPIG-HepG2 | 0.7809 | 0.8535 | 0.7952 | 0.8772 | **0.8857** |
| PRPF8-HepG2 | 0.8708 | 0.9181 | 0.8676 | 0.9025 | **0.9377** |
| PRPF8-K562 | 0.7727 | 0.8463 | 0.8125 | 0.8817 | **0.8904** |
| PUM1-K562 | 0.7439 | 0.7494 | 0.7377 | 0.7661 | **0.7932** |
| PUM2-K562 | 0.9421 | 0.9524 | 0.9450 | 0.9534 | **0.9645** |
| RBM15-K562 | 0.7430 | 0.8086 | 0.8097 | 0.7893 | **0.8518** |
| RPS3-HepG2 | 0.6909 | 0.7614 | 0.7342 | 0.7739 | **0.7920** |
| RPS3-K562 | 0.6916 | 0.7525 | 0.6917 | 0.7292 | **0.7815** |
| SF3B4-HepG2 | 0.8394 | 0.9154 | 0.8795 | 0.9430 | **0.9517** |
| SF3B4-K562 | 0.8005 | 0.8884 | 0.8435 | 0.9078 | **0.9255** |
| SND1-HepG2 | 0.7119 | 0.7724 | 0.7028 | 0.7650 | **0.7866** |
| SND1-K562 | 0.7032 | 0.7642 | 0.7271 | 0.7509 | **0.8075** |
| SUB1-HepG2 | 0.8827 | 0.8808 | 0.8940 | 0.8907 | **0.9122** |
| UCHL5-K562 | 0.8136 | 0.8470 | 0.8310 | 0.8723 | **0.8729** |
| UPF1-HepG2 | 0.7823 | 0.8109 | 0.8011 | 0.8329 | **0.8472** |
| UPF1-K562 | 0.7795 | 0.7984 | 0.7798 | 0.8248 | **0.8353** |
| YBX3-K562 | 0.7399 | 0.7903 | 0.7400 | 0.8082 | **0.8312** |
| ZNF622-K562 | 0.7961 | 0.8330 | 0.8159 | 0.8675 | **0.8750** |
| ZNF800-K562 | 0.7910 | 0.8236 | 0.7970 | 0.8147 | **0.8547** |

**Table S11** AUPR of comparative methods on each dataset using only the one-hot encoding sequence feature.

| Datasets | Methods | | | | |
| --- | --- | --- | --- | --- | --- |
|  | DeepBind | DeepRiPe | PrismNet | HDRNet | diPaRIS |
| AKAP1-HepG2 | 0.7852 | 0.8007 | 0.7872 | 0.8116 | **0.8356** |
| AQR-HepG2 | 0.7598 | 0.8014 | 0.7728 | 0.7940 | **0.8406** |
| AQR-K562 | 0.8104 | 0.8578 | 0.8146 | 0.8632 | **0.8819** |
| BCLAF1-HepG2 | 0.8502 | 0.8529 | 0.8431 | 0.8472 | **0.8786** |
| BUD13-HepG2 | 0.7423 | 0.7780 | 0.7570 | 0.7747 | **0.8024** |
| BUD13-K562 | 0.7364 | 0.7795 | 0.7767 | 0.7999 | **0.8272** |
| DDX24-K562 | 0.7390 | 0.7848 | 0.7505 | **0.8091** | **0.8091** |
| DDX3X-HepG2 | 0.8546 | 0.8553 | 0.8526 | 0.8497 | **0.8708** |
| DDX3X-K562 | 0.8470 | 0.8463 | 0.8391 | 0.8691 | **0.8746** |
| EFTUD2-HepG2 | 0.8088 | 0.8153 | 0.8107 | 0.7952 | **0.8469** |
| EFTUD2-K562 | 0.7762 | 0.7995 | 0.7889 | 0.7944 | **0.8289** |
| FAM120A-K562 | 0.7969 | 0.8034 | 0.7920 | 0.7933 | **0.8311** |
| FMR1-K562 | 0.7423 | 0.7991 | 0.7723 | 0.7948 | **0.8137** |
| FXR2-K562 | 0.7600 | 0.7679 | 0.7757 | 0.7712 | **0.8069** |
| G3BP1-HepG2 | 0.7829 | 0.7874 | 0.7758 | 0.7890 | **0.8148** |
| GRWD1-HepG2 | 0.7772 | 0.7919 | 0.7796 | 0.8066 | **0.8232** |
| GRWD1-K562 | 0.7968 | 0.8040 | 0.7883 | 0.8121 | **0.8346** |
| IGF2BP1-K562 | 0.8324 | 0.8545 | 0.8284 | 0.8294 | **0.8701** |
| IGF2BP2-K562 | 0.7892 | 0.8080 | 0.7910 | 0.7998 | **0.8311** |
| LARP4-HepG2 | 0.7309 | 0.7365 | 0.7391 | 0.7421 | **0.7623** |
| LIN28B-K562 | 0.7606 | 0.7839 | 0.7687 | 0.7776 | **0.8072** |
| METAP2-K562 | 0.7698 | 0.7683 | 0.7550 | 0.7709 | **0.7928** |
| PABPC4-K562 | 0.7270 | 0.7360 | 0.7389 | 0.7587 | **0.7597** |
| PABPN1-HepG2 | 0.7191 | 0.7303 | 0.7176 | 0.7418 | **0.7644** |
| PCBP2-HepG2 | 0.9207 | 0.9222 | 0.9095 | 0.8765 | **0.9363** |
| PPIG-HepG2 | 0.7855 | 0.8312 | 0.7921 | 0.8390 | **0.8504** |
| PRPF8-HepG2 | 0.8517 | 0.8852 | 0.8367 | 0.8646 | **0.8979** |
| PRPF8-K562 | 0.7667 | 0.8271 | 0.7810 | 0.8462 | **0.8567** |
| PUM1-K562 | 0.7583 | 0.7611 | 0.7542 | 0.7724 | **0.7858** |
| PUM2-K562 | 0.9025 | 0.9108 | 0.8886 | 0.8876 | **0.9353** |
| RBM15-K562 | 0.7719 | 0.8046 | 0.7887 | 0.7856 | **0.8342** |
| RPS3-HepG2 | 0.7423 | 0.7654 | 0.7535 | 0.7759 | **0.7908** |
| RPS3-K562 | 0.7393 | 0.7610 | 0.7592 | 0.7718 | **0.7949** |
| SF3B4-HepG2 | 0.8203 | 0.8728 | 0.8439 | 0.8985 | **0.9097** |
| SF3B4-K562 | 0.8032 | 0.8610 | 0.8188 | 0.8717 | **0.8914** |
| SND1-HepG2 | 0.7444 | 0.7762 | 0.7322 | 0.7395 | **0.7894** |
| SND1-K562 | 0.7421 | 0.7745 | 0.7595 | 0.7901 | **0.8136** |
| SUB1-HepG2 | 0.8457 | 0.8504 | 0.8524 | 0.8485 | **0.8696** |
| UCHL5-K562 | 0.7935 | 0.8221 | 0.8041 | 0.8319 | **0.8417** |
| UPF1-HepG2 | 0.7881 | 0.7987 | 0.7916 | 0.8044 | **0.8225** |
| UPF1-K562 | 0.7893 | 0.7940 | 0.7776 | 0.8029 | **0.8194** |
| YBX3-K562 | 0.7634 | 0.7877 | 0.7469 | 0.8017 | **0.8177** |
| ZNF622-K562 | 0.7872 | 0.8127 | 0.7981 | 0.8383 | **0.8429** |
| ZNF800-K562 | 0.7982 | 0.8014 | 0.7893 | 0.8101 | **0.8406** |

**Table S12** F1-score of comparative methods on each dataset using only the one-hot encoding sequence feature.

| Datasets | Methods | | | | |
| --- | --- | --- | --- | --- | --- |
|  | DeepBind | DeepRiPe | PrismNet | HDRNet | diPaRIS |
| AKAP1-HepG2 | 0.7211 | 0.7378 | 0.6984 | 0.7666 | **0.7883** |
| AQR-HepG2 | 0.6910 | 0.7416 | 0.6695 | 0.7466 | **0.7969** |
| AQR-K562 | 0.7617 | 0.8111 | 0.7429 | 0.8225 | **0.8497** |
| BCLAF1-HepG2 | 0.8097 | 0.8031 | 0.7742 | 0.8136 | **0.8387** |
| BUD13-HepG2 | 0.6676 | 0.6898 | 0.6390 | 0.7154 | **0.7334** |
| BUD13-K562 | 0.6601 | 0.7058 | 0.6772 | 0.7035 | **0.7595** |
| DDX24-K562 | 0.6619 | 0.7125 | 0.6453 | **0.7580** | 0.7476 |
| DDX3X-HepG2 | 0.7864 | 0.7989 | 0.7745 | 0.7940 | **0.8229** |
| DDX3X-K562 | 0.7788 | 0.7898 | 0.7542 | 0.7977 | **0.8349** |
| EFTUD2-HepG2 | 0.7475 | 0.7527 | 0.7036 | 0.6763 | **0.7968** |
| EFTUD2-K562 | 0.7108 | 0.7409 | 0.6895 | 0.6953 | **0.7808** |
| FAM120A-K562 | 0.7425 | 0.7450 | 0.6870 | 0.7129 | **0.7793** |
| FMR1-K562 | 0.6726 | 0.7398 | 0.6750 | 0.7308 | **0.7609** |
| FXR2-K562 | 0.6927 | 0.6769 | 0.6923 | 0.7025 | **0.7502** |
| G3BP1-HepG2 | 0.7208 | 0.7140 | 0.6977 | 0.7064 | **0.7627** |
| GRWD1-HepG2 | 0.7161 | 0.7265 | 0.6910 | 0.7571 | **0.7636** |
| GRWD1-K562 | 0.7428 | 0.7479 | 0.6642 | 0.6840 | **0.7804** |
| IGF2BP1-K562 | 0.7839 | 0.8039 | 0.7718 | 0.7955 | **0.8376** |
| IGF2BP2-K562 | 0.7247 | 0.7506 | 0.7072 | 0.7155 | **0.7741** |
| LARP4-HepG2 | 0.6479 | 0.6476 | 0.6113 | 0.5594 | **0.6869** |
| LIN28B-K562 | 0.6911 | 0.7098 | 0.6792 | 0.5839 | **0.7433** |
| METAP2-K562 | 0.7083 | 0.6880 | 0.5578 | 0.7116 | **0.7325** |
| PABPC4-K562 | 0.6461 | 0.6494 | 0.6104 | 0.6503 | **0.6782** |
| PABPN1-HepG2 | 0.6384 | 0.6477 | 0.5283 | 0.5798 | **0.6931** |
| PCBP2-HepG2 | 0.8900 | 0.8915 | 0.8770 | 0.8476 | **0.9179** |
| PPIG-HepG2 | 0.7212 | 0.7750 | 0.7327 | 0.7759 | **0.8090** |
| PRPF8-HepG2 | 0.8082 | 0.8462 | 0.7235 | 0.8209 | **0.8663** |
| PRPF8-K562 | 0.6935 | 0.7620 | 0.6112 | 0.7705 | **0.8169** |
| PUM1-K562 | 0.6899 | 0.6724 | 0.6211 | 0.6820 | **0.7194** |
| PUM2-K562 | 0.8716 | 0.8858 | 0.8411 | 0.8584 | **0.9120** |
| RBM15-K562 | 0.7111 | 0.7340 | 0.6533 | 0.7306 | **0.7771** |
| RPS3-HepG2 | 0.6678 | 0.6932 | 0.6644 | 0.6611 | **0.7224** |
| RPS3-K562 | 0.6586 | 0.6932 | 0.6722 | 0.7057 | **0.7272** |
| SF3B4-HepG2 | 0.7778 | 0.8330 | 0.7781 | 0.8696 | **0.8828** |
| SF3B4-K562 | 0.7445 | 0.8127 | 0.7558 | 0.8034 | **0.8588** |
| SND1-HepG2 | 0.6688 | 0.7056 | 0.5807 | 0.5460 | **0.7286** |
| SND1-K562 | 0.6684 | 0.6997 | 0.6860 | 0.7253 | **0.7542** |
| SUB1-HepG2 | 0.7987 | 0.7990 | 0.8006 | 0.7501 | **0.8369** |
| UCHL5-K562 | 0.7362 | 0.7653 | 0.7206 | 0.7127 | **0.7919** |
| UPF1-HepG2 | 0.7263 | 0.7259 | 0.7341 | 0.7591 | **0.7697** |
| UPF1-K562 | 0.7381 | 0.7253 | 0.6837 | 0.7177 | **0.7687** |
| YBX3-K562 | 0.7009 | 0.7136 | 0.6333 | 0.7335 | **0.7602** |
| ZNF622-K562 | 0.7281 | 0.7512 | 0.7237 | 0.7643 | **0.7888** |
| ZNF800-K562 | 0.7321 | 0.7294 | 0.6548 | 0.7459 | **0.7827** |

**Table S13** Precision of comparative methods on each dataset using only the one-hot encoding sequence feature.

| Datasets | Methods | | | | |
| --- | --- | --- | --- | --- | --- |
|  | DeepBind | DeepRiPe | PrismNet | HDRNet | diPaRIS |
| AKAP1-HepG2 | 0.6804 | 0.7238 | 0.7010 | 0.6818 | **0.7604** |
| AQR-HepG2 | 0.6342 | 0.7186 | 0.6971 | 0.6416 | **0.7708** |
| AQR-K562 | 0.7086 | 0.8077 | 0.7290 | 0.8055 | **0.8274** |
| BCLAF1-HepG2 | 0.7751 | 0.8058 | 0.7986 | 0.7336 | **0.8389** |
| BUD13-HepG2 | 0.6028 | 0.7144 | 0.6499 | 0.5773 | **0.7276** |
| BUD13-K562 | 0.6163 | 0.7040 | 0.7192 | 0.7650 | **0.7876** |
| DDX24-K562 | 0.6166 | 0.7073 | 0.6474 | 0.6776 | **0.7403** |
| DDX3X-HepG2 | **0.8355** | 0.8157 | 0.8260 | 0.7968 | 0.8307 |
| DDX3X-K562 | 0.8195 | 0.8022 | 0.8086 | 0.8630 | **0.8290** |
| EFTUD2-HepG2 | 0.7200 | 0.7501 | 0.7565 | **0.8098** | 0.7965 |
| EFTUD2-K562 | 0.6765 | 0.7172 | 0.7137 | **0.7678** | 0.7545 |
| FAM120A-K562 | 0.6843 | 0.7236 | 0.7247 | 0.6613 | **0.7653** |
| FMR1-K562 | 0.6130 | 0.7199 | 0.6601 | 0.6406 | **0.7321** |
| FXR2-K562 | 0.6268 | 0.7110 | 0.6640 | 0.6184 | **0.7211** |
| G3BP1-HepG2 | 0.6709 | 0.7163 | 0.6314 | 0.7062 | **0.7277** |
| GRWD1-HepG2 | 0.6613 | 0.7120 | 0.6970 | 0.6836 | **0.7582** |
| GRWD1-K562 | 0.6873 | 0.7229 | 0.7310 | **0.8007** | 0.7719 |
| IGF2BP1-K562 | 0.7553 | 0.8044 | 0.7442 | 0.6954 | **0.7986** |
| IGF2BP2-K562 | 0.6948 | 0.7243 | 0.7112 | 0.7163 | **0.7792** |
| LARP4-HepG2 | 0.6033 | 0.6465 | 0.6461 | **0.6725** | 0.6677 |
| LIN28B-K562 | 0.6428 | 0.7060 | 0.6636 | **0.7860** | 0.7357 |
| METAP2-K562 | 0.6376 | 0.6903 | 0.7027 | 0.6016 | **0.7084** |
| PABPC4-K562 | 0.5997 | 0.6410 | 0.6340 | 0.6309 | **0.6849** |
| PABPN1-HepG2 | 0.5823 | 0.6247 | 0.6323 | 0.6485 | **0.6673** |
| PCBP2-HepG2 | 0.8953 | 0.9003 | 0.8826 | 0.7883 | **0.9086** |
| PPIG-HepG2 | 0.6898 | 0.7686 | 0.6705 | **0.7930** | 0.7839 |
| PRPF8-HepG2 | 0.7879 | 0.8473 | 0.8293 | 0.8069 | **0.8593** |
| PRPF8-K562 | 0.6726 | 0.7776 | 0.7644 | **0.8350** | 0.7941 |
| PUM1-K562 | 0.6431 | 0.6915 | 0.6470 | 0.7056 | **0.7125** |
| PUM2-K562 | 0.8596 | 0.8691 | 0.8586 | 0.8166 | **0.9150** |
| RBM15-K562 | 0.6381 | 0.7440 | 0.7729 | 0.6591 | **0.7788** |
| RPS3-HepG2 | 0.6145 | 0.6702 | 0.6357 | 0.6618 | **0.7151** |
| RPS3-K562 | 0.5946 | 0.6562 | 0.6109 | 0.6403 | **0.7152** |
| SF3B4-HepG2 | 0.7038 | 0.8251 | 0.7828 | 0.8500 | **0.8719** |
| SF3B4-K562 | 0.7140 | 0.8130 | 0.7358 | **0.8638** | 0.8482 |
| SND1-HepG2 | 0.6261 | 0.6887 | 0.6372 | **0.7319** | 0.7031 |
| SND1-K562 | 0.6207 | 0.6921 | 0.6027 | 0.6527 | **0.7351** |
| SUB1-HepG2 | 0.7861 | 0.8053 | 0.7813 | **0.8547** | 0.8064 |
| UCHL5-K562 | 0.6956 | 0.7574 | 0.7434 | **0.8628** | 0.7828 |
| UPF1-HepG2 | 0.6898 | 0.7385 | 0.6528 | 0.6769 | **0.7510** |
| UPF1-K562 | 0.6541 | 0.7223 | 0.6605 | **0.7661** | 0.7342 |
| YBX3-K562 | 0.6352 | 0.7214 | 0.6796 | 0.7235 | **0.7469** |
| ZNF622-K562 | 0.6814 | 0.7457 | 0.7060 | **0.8075** | 0.7916 |
| ZNF800-K562 | 0.7146 | 0.7489 | 0.7317 | 0.7204 | **0.7869** |

**Table S14** Recall of comparative methods on each dataset using only the one-hot encoding sequence feature.

| Datasets | Methods | | | | |
| --- | --- | --- | --- | --- | --- |
|  | DeepBind | DeepRiPe | PrismNet | HDRNet | diPaRIS |
| AKAP1-HepG2 | 0.7777 | 0.7534 | 0.7441 | **0.8807** | 0.8193 |
| AQR-HepG2 | 0.7647 | 0.7691 | 0.6961 | **0.8933** | 0.8249 |
| AQR-K562 | 0.8243 | 0.8152 | 0.8010 | 0.8426 | **0.8734** |
| BCLAF1-HepG2 | 0.8484 | 0.8022 | 0.7739 | **0.9213** | 0.8391 |
| BUD13-HepG2 | 0.7604 | 0.6754 | 0.7218 | **0.9420** | 0.7434 |
| BUD13-K562 | 0.7147 | 0.7150 | 0.6691 | 0.6610 | **0.7335** |
| DDX24-K562 | 0.7204 | 0.7214 | 0.7016 | **0.8768** | 0.7558 |
| DDX3X-HepG2 | 0.7439 | 0.7863 | 0.7529 | 0.8035 | **0.8173** |
| DDX3X-K562 | 0.7460 | 0.7837 | 0.7344 | 0.7515 | **0.8416** |
| EFTUD2-HepG2 | 0.7928 | 0.7651 | 0.7245 | 0.5912 | **0.7974** |
| EFTUD2-K562 | 0.7507 | 0.7691 | 0.7272 | 0.6485 | **0.8105** |
| FAM120A-K562 | 0.8174 | 0.7711 | 0.7160 | **0.8494** | 0.7944 |
| FMR1-K562 | 0.7506 | 0.7632 | 0.7723 | **0.8919** | 0.7942 |
| FXR2-K562 | 0.7802 | 0.6501 | 0.7767 | **0.8530** | 0.7827 |
| G3BP1-HepG2 | 0.7851 | 0.7182 | **0.8400** | 0.7381 | 0.8022 |
| GRWD1-HepG2 | 0.7858 | 0.7476 | 0.7251 | **0.8583** | 0.7730 |
| GRWD1-K562 | **0.8106** | 0.7793 | 0.6879 | 0.6554 | 0.7901 |
| IGF2BP1-K562 | 0.8172 | 0.8060 | 0.8240 | **0.9299** | 0.8819 |
| IGF2BP2-K562 | 0.7597 | 0.7816 | 0.7464 | 0.7579 | **0.7703** |
| LARP4-HepG2 | 0.7133 | 0.6519 | 0.6580 | 0.6229 | **0.7083** |
| LIN28B-K562 | **0.7582** | 0.7178 | 0.7442 | 0.5280 | 0.7523 |
| METAP2-K562 | 0.8025 | 0.6957 | 0.6041 | **0.8816** | 0.7598 |
| PABPC4-K562 | 0.7102 | 0.6605 | 0.6871 | **0.7629** | 0.6719 |
| PABPN1-HepG2 | 0.7162 | 0.6752 | 0.6094 | 0.6609 | **0.7221** |
| PCBP2-HepG2 | 0.8872 | 0.8854 | 0.8773 | 0.9259 | **0.9283** |
| PPIG-HepG2 | 0.7612 | 0.7836 | 0.8277 | 0.7715 | **0.8369** |
| PRPF8-HepG2 | 0.8304 | 0.8458 | 0.6840 | 0.8443 | **0.8735** |
| PRPF8-K562 | 0.7329 | 0.7505 | 0.6028 | 0.7198 | **0.8416** |
| PUM1-K562 | **0.7479** | 0.6599 | 0.7216 | 0.6751 | 0.7282 |
| PUM2-K562 | 0.8869 | 0.9038 | 0.8429 | **0.9197** | 0.9090 |
| RBM15-K562 | 0.8078 | 0.7257 | 0.6130 | **0.8259** | 0.7763 |
| RPS3-HepG2 | 0.7398 | 0.7236 | 0.7449 | **0.7736** | 0.7327 |
| RPS3-K562 | 0.7678 | 0.7374 | **0.8053** | 0.7962 | 0.7407 |
| SF3B4-HepG2 | 0.8743 | 0.8420 | 0.8038 | 0.8944 | **0.8949** |
| SF3B4-K562 | 0.7805 | 0.8158 | 0.8006 | 0.7587 | **0.8700** |
| SND1-HepG2 | 0.7294 | 0.7290 | 0.6538 | 0.4808 | **0.7566** |
| SND1-K562 | 0.7268 | 0.7112 | 0.8320 | **0.8487** | 0.7752 |
| SUB1-HepG2 | 0.8164 | 0.7945 | 0.8424 | 0.6856 | **0.8701** |
| UCHL5-K562 | 0.7862 | 0.7755 | 0.7326 | 0.6110 | **0.8023** |
| UPF1-HepG2 | 0.7695 | 0.7173 | 0.8599 | **0.8663** | 0.7898 |
| UPF1-K562 | **0.8485** | 0.7305 | 0.7878 | 0.6772 | 0.8081 |
| YBX3-K562 | **0.7847** | 0.7076 | 0.6331 | 0.7558 | 0.7748 |
| ZNF622-K562 | 0.7862 | 0.7588 | 0.7800 | 0.7349 | **0.7867** |
| ZNF800-K562 | 0.7574 | 0.7126 | 0.6836 | **0.7928** | 0.7802 |

**Table S15** The average performance of diPaRIS full model, using only the one-hot encoding sequence feature, without our icSHAPE-DS coding scheme and attention mechanisms. The metrics used for comparison include AUC, AUPR, ACC, F1-score, precision, and recall.

| Compared Group | Metrics  0.7305  0.7878  0.6772  0.7890 | | | | | |
| --- | --- | --- | --- | --- | --- | --- |
|  | AUC | ACC | Precision | Recall | F1-score | AUPR |
| Full model | **0.9381** | **0.8667** | **0.8669** | **0.8710** | **0.8685** | **0.9017** |
| Only one-hot encoding sequence feature | 0.8543 | 0.7793 | 0.7710 | 0.7985 | 0.7837 | 0.8352 |
| Without Attention | 0.9191 | 0.8415 | 0.8407 | 0.8431 | 0.8414 | 0.8810 |
| Without icSHAPE-DS | 0.9227 | 0.8456 | 0.8468 | 0.8445 | 0.8451 | 0.8845 |

**Table S16** Protein-binding domains and their corresponding proteins. Information on structural domains within proteins was obtained from the NCBI Reference Sequences (RefSeq) database^[2]^ and the Pfam database^[3]^.

| Protein-binding domains | RRM | ZNF | WD40 | Helicase_C | KH | RGG | G-patch |
| --- | --- | --- | --- | --- | --- | --- | --- |
| Proteins | AKAP1 | UCHL5 | GRWD1 | AQR | IGF2BP1 | FMR1 | EFTUD2 |
|  | AQR | ZNF622 |  | DDX24 | IGF2BP2 |  |  |
|  | YBX3 | ZNF800 |  | DDX3X | PCBP2 |  |  |
|  | DDX24 |  |  |  |  |  |  |
|  | LARP4 |  |  |  |  |  |  |
|  | LIN28B |  |  |  |  |  |  |
|  | PUM1 |  |  |  |  |  |  |
|  | PUM2 |  |  |  |  |  |  |
|  | RBM15 |  |  |  |  |  |  |
|  | SF3B4 |  |  |  |  |  |  |
|  | SUB1 |  |  |  |  |  |  |

**Table S17** The t-test results of cross-test predicted scores between each pair of comparative methods. The table presents the p-values from these pairwise t-tests. Typically, a p-value less than 0.05 is considered statistically significant, while p < 0.0001 indicates an extremely statistically significant difference.

| Cell lines | Methods | DeepRiPe | PrismNet | HDRNet | diPaRIS |
| --- | --- | --- | --- | --- | --- |
| HepG2 | DeepBind | < 0.0001 | < 0.0001 | < 0.0001 | < 0.0001 |
|  | DeepRiPe |  | < 0.0001 | < 0.0001 | < 0.0001 |
|  | PrismNet |  |  | 0.0147 | 0.0033 |
|  | HDRNet |  |  |  | 0.0013 |
| K562 | DeepBind | < 0.0001 | < 0.0001 | < 0.0001 | < 0.0001 |
|  | DeepRiPe |  | < 0.0001 | < 0.0001 | < 0.0001 |
|  | PrismNet |  |  | 0.0307 | 0.0053 |
|  | HDRNet |  |  |  | 0.0002 |

**Table S18** ACC of cross-test on each dataset. 'Without Attention' refers to the performance metrics when the attention mechanism is removed from the diPaRIS model, while 'Without icSHAPE-DS' indicates the metrics obtained by using raw data without incorporating icSHAPE-DS encoding.

| Cell lines | Proteins | Methods | | | | | | |
| --- | --- | --- | --- | --- | --- | --- | --- | --- |
|  |  | DeepBind | DeepRiPe | PrismNet | HDRNet | diPaRIS | | |
|  |  |  |  |  |  |  | without Attention | without icSHAPE-DS |
| HepG2 | AQR | 0.6449 | 0.6995 | 0.8177 | 0.8331 | **0.8678** | 0.8471 | 0.8587 |
|  | BUD13 | 0.5974 | 0.6836 | 0.7166 | 0.7872 | **0.8186** | 0.7975 | 0.8035 |
|  | DDX3X | 0.7673 | 0.7692 | 0.8342 | 0.8385 | **0.8633** | 0.8445 | 0.8546 |
|  | EFTUD2 | 0.6594 | 0.7086 | 0.8165 | 0.8287 | **0.8597** | 0.8378 | 0.8368 |
|  | GRWD1 | 0.7084 | 0.7370 | 0.8144 | 0.8013 | **0.8372** | 0.8181 | 0.8215 |
|  | PRPF8 | 0.7247 | 0.7442 | 0.8132 | 0.8360 | **0.8491** | 0.8257 | 0.8366 |
|  | RPS3 | 0.6152 | 0.6933 | 0.7948 | 0.7915 | **0.8306** | 0.8201 | 0.8242 |
|  | SF3B4 | 0.6649 | 0.7234 | 0.7954 | 0.8082 | **0.8268** | 0.7933 | 0.7917 |
|  | SND1 | 0.6034 | 0.6670 | 0.7739 | 0.7765 | **0.8021** | 0.7862 | 0.8027 |
|  | UPF1 | 0.6576 | 0.6832 | 0.7414 | 0.7765 | **0.8099** | 0.7923 | 0.7890 |
|  | Averages | 0.6643 | 0.7109 | 0.7918 | 0.8078 | **0.8365** | 0.8163 | 0.8219 |
| K562 | AQR | 0.6800 | 0.7248 | 0.7346 | 0.8297 | **0.8525** | 0.8431 | 0.8466 |
|  | BUD13 | 0.6299 | 0.6928 | 0.7851 | 0.8154 | **0.8342** | 0.8126 | 0.8214 |
|  | DDX3X | 0.7822 | 0.7897 | 0.8259 | 0.8276 | **0.8566** | 0.8356 | 0.8451 |
|  | EFTUD2 | 0.7001 | 0.7403 | 0.8317 | 0.8304 | **0.8558** | 0.8435 | 0.8427 |
|  | GRWD1 | 0.6605 | 0.6901 | 0.7463 | 0.7555 | **0.7809** | 0.7665 | 0.7681 |
|  | PRPF8 | 0.6951 | 0.7635 | 0.8402 | 0.8520 | **0.8827** | 0.8595 | 0.8716 |
|  | RPS3 | 0.6012 | 0.6610 | 0.7359 | 0.7427 | **0.7813** | 0.7690 | 0.7721 |
|  | SF3B4 | 0.7505 | 0.8310 | 0.8512 | 0.8802 | **0.9062** | 0.8674 | 0.8829 |
|  | SND1 | 0.6138 | 0.6642 | 0.7468 | 0.7600 | **0.7824** | 0.7531 | 0.7637 |
|  | UPF1 | 0.6759 | 0.6914 | 0.7436 | 0.7771 | **0.8121** | 0.7967 | 0.7986 |
|  | Averages | 0.6789 | 0.7249 | 0.7841 | 0.8071 | **0.8345** | 0.8147 | 0.8213 |

**Table S19** AUC of cross-test on each dataset. 'Without Attention' refers to the performance metrics when the attention mechanism is removed from the diPaRIS model, while 'Without icSHAPE-DS' indicates the metrics obtained by using raw data without incorporating icSHAPE-DS encoding.

| Cell lines | Proteins | Methods | | | | | | |
| --- | --- | --- | --- | --- | --- | --- | --- | --- |
|  |  | DeepBind | DeepRiPe | PrismNet | HDRNet | diPaRIS | | |
|  |  |  |  |  |  |  | without Attention | without icSHAPE-DS |
| HepG2 | AQR | 0.7012 | 0.7669 | 0.9211 | 0.9114 | **0.9414** | 0.9231 | 0.9338 |
|  | BUD13 | 0.6339 | 0.7505 | 0.8499 | 0.8705 | **0.8941** | 0.8778 | 0.8858 |
|  | DDX3X | 0.8263 | 0.8456 | 0.9170 | 0.9159 | **0.9345** | 0.9248 | 0.9306 |
|  | EFTUD2 | 0.7122 | 0.7791 | 0.9123 | 0.9094 | **0.9328** | 0.9135 | 0.9137 |
|  | GRWD1 | 0.7832 | 0.8167 | 0.9063 | 0.8861 | **0.9209** | 0.9049 | 0.9064 |
|  | PRPF8 | 0.7848 | 0.8161 | 0.9003 | 0.9130 | **0.9293** | 0.9082 | 0.9200 |
|  | RPS3 | 0.6603 | 0.7614 | 0.9030 | 0.8800 | **0.9155** | 0.9063 | 0.9045 |
|  | SF3B4 | 0.7214 | 0.7918 | 0.8857 | 0.8985 | **0.9186** | 0.8830 | 0.8870 |
|  | SND1 | 0.6507 | 0.7268 | 0.8767 | 0.8553 | **0.9020** | 0.8752 | 0.8757 |
|  | UPF1 | 0.7197 | 0.7524 | 0.8510 | 0.8581 | **0.8929** | 0.8720 | 0.8710 |
|  | Averages | 0.7194 | 0.7807 | 0.8923 | 0.8898 | **0.9182** | 0.8989 | 0.9029 |
| K562 | AQR | 0.7332 | 0.7832 | 0.8873 | 0.9016 | **0.9180** | 0.9132 | 0.9179 |
|  | BUD13 | 0.6794 | 0.7610 | 0.8716 | 0.8940 | **0.9108** | 0.8957 | 0.9040 |
|  | DDX3X | 0.8494 | 0.8696 | 0.9099 | 0.9064 | **0.9283** | 0.9195 | 0.9220 |
|  | EFTUD2 | 0.7650 | 0.8186 | 0.9134 | 0.9148 | **0.9321** | 0.9227 | 0.9229 |
|  | GRWD1 | 0.7211 | 0.7664 | 0.8336 | 0.8481 | **0.8738** | 0.8506 | 0.8575 |
|  | PRPF8 | 0.7631 | 0.8375 | 0.9273 | 0.9307 | **0.9528** | 0.9355 | 0.9438 |
|  | RPS3 | 0.6426 | 0.7193 | 0.8337 | 0.8377 | **0.8675** | 0.8577 | 0.8525 |
|  | SF3B4 | 0.8283 | 0.9037 | 0.9304 | 0.9514 | **0.9664** | 0.9435 | 0.9557 |
|  | SND1 | 0.6591 | 0.7290 | 0.8333 | 0.8442 | **0.8690** | 0.8414 | 0.8516 |
|  | UPF1 | 0.7405 | 0.7688 | 0.8632 | 0.8617 | **0.8906** | 0.8793 | 0.8834 |
|  | Averages | 0.7382 | 0.7957 | 0.8804 | 0.8891 | **0.9109** | 0.8959 | 0.9011 |

**Table S20** AUPR of cross-test on each dataset. 'Without Attention' refers to the performance metrics when the attention mechanism is removed from the diPaRIS model, while 'Without icSHAPE-DS' indicates the metrics obtained by using raw data without incorporating icSHAPE-DS encoding.

| Cell lines | Proteins | Methods | | | | | | |
| --- | --- | --- | --- | --- | --- | --- | --- | --- |
|  |  | DeepBind | DeepRiPe | PrismNet | HDRNet | diPaRIS | | |
|  |  |  |  |  |  |  | without Attention | without icSHAPE-DS |
| HepG2 | AQR | 0.7337 | 0.7761 | 0.8648 | 0.8766 | **0.9010** | 0.8873 | 0.8959 |
|  | BUD13 | 0.7046 | 0.7635 | 0.8027 | 0.8414 | **0.8652** | 0.8491 | 0.8595 |
|  | DDX3X | 0.8298 | 0.8338 | 0.8817 | 0.8801 | **0.9019** | 0.8931 | 0.8972 |
|  | EFTUD2 | 0.7430 | 0.7808 | 0.8608 | 0.8779 | **0.8981** | 0.8841 | 0.8810 |
|  | GRWD1 | 0.7854 | 0.8060 | 0.8609 | 0.8507 | **0.8757** | 0.8639 | 0.8654 |
|  | PRPF8 | 0.7935 | 0.8105 | 0.8671 | 0.8814 | **0.8959** | 0.8760 | 0.8842 |
|  | RPS3 | 0.7140 | 0.7723 | 0.8485 | 0.8475 | **0.8715** | 0.8674 | 0.8685 |
|  | SF3B4 | 0.7459 | 0.7937 | 0.8493 | 0.8708 | **0.8860** | 0.8582 | 0.8590 |
|  | SND1 | 0.7022 | 0.7496 | 0.8341 | 0.8330 | **0.8506** | 0.8395 | 0.8545 |
|  | UPF1 | 0.7435 | 0.7610 | 0.8163 | 0.8332 | **0.8623** | 0.8464 | 0.8463 |
|  | Averages | 0.7496 | 0.7847 | 0.8486 | 0.8593 | **0.8808** | 0.8665 | 0.8712 |
| K562 | AQR | 0.7617 | 0.7942 | 0.8173 | 0.8760 | **0.8928** | 0.8870 | 0.8916 |
|  | BUD13 | 0.7250 | 0.7690 | 0.8430 | 0.8650 | **0.8758** | 0.8647 | 0.8666 |
|  | DDX3X | 0.8379 | 0.8421 | 0.8713 | 0.8714 | **0.8925** | 0.8744 | 0.8887 |
|  | EFTUD2 | 0.7748 | 0.8057 | 0.8722 | 0.8754 | **0.8946** | 0.8833 | 0.8842 |
|  | GRWD1 | 0.7418 | 0.7657 | 0.8124 | 0.8291 | **0.8504** | 0.8318 | 0.8334 |
|  | PRPF8 | 0.7718 | 0.8228 | 0.8809 | 0.8920 | **0.9088** | 0.8946 | 0.9036 |
|  | RPS3 | 0.7016 | 0.7464 | 0.8090 | 0.8141 | **0.8391** | 0.8291 | 0.8321 |
|  | SF3B4 | 0.8146 | 0.8734 | 0.8934 | 0.9125 | **0.9316** | 0.9047 | 0.9119 |
|  | SND1 | 0.7062 | 0.7470 | 0.8108 | 0.8210 | **0.8390** | 0.8178 | 0.8260 |
|  | UPF1 | 0.7697 | 0.7669 | 0.8207 | 0.8383 | **0.8594** | 0.8505 | 0.8541 |
|  | Averages | 0.7605 | 0.7933 | 0.8431 | 0.8595 | **0.8784** | 0.8638 | 0.8692 |

**Table S21** F1-score of cross-test on each dataset. 'Without Attention' refers to the performance metrics when the attention mechanism is removed from the diPaRIS model, while 'Without icSHAPE-DS' indicates the metrics obtained by using raw data without incorporating icSHAPE-DS encoding.

| Cell lines | Proteins | Methods | | | | | | |
| --- | --- | --- | --- | --- | --- | --- | --- | --- |
|  |  | DeepBind | DeepRiPe | PrismNet | HDRNet | diPaRIS | | |
|  |  |  |  |  |  |  | without Attention | without icSHAPE-DS |
| HepG2 | AQR | 0.6401 | 0.7051 | 0.8336 | 0.8299 | **0.8676** | 0.8443 | 0.8564 |
|  | BUD13 | 0.6129 | 0.6866 | 0.7529 | 0.7890 | **0.8156** | 0.7939 | 0.7892 |
|  | DDX3X | 0.7500 | 0.7471 | 0.8289 | 0.8369 | **0.8618** | 0.8334 | 0.8478 |
|  | EFTUD2 | 0.6460 | 0.6916 | 0.8289 | 0.8192 | **0.8560** | 0.8297 | 0.8316 |
|  | GRWD1 | 0.7247 | 0.7526 | 0.8214 | 0.8047 | **0.8425** | 0.8174 | 0.8234 |
|  | PRPF8 | 0.7175 | 0.7256 | 0.8031 | 0.8296 | **0.8493** | 0.8151 | 0.8275 |
|  | RPS3 | 0.6218 | 0.7000 | 0.8180 | 0.7922 | **0.8365** | 0.8153 | 0.8232 |
|  | SF3B4 | 0.6500 | 0.7058 | 0.7980 | 0.7855 | **0.8076** | 0.7686 | 0.7641 |
|  | SND1 | 0.5870 | 0.6617 | 0.7786 | 0.7724 | **0.8157** | 0.7869 | 0.7959 |
|  | UPF1 | 0.6578 | 0.6700 | 0.7633 | 0.7847 | **0.8159** | 0.7851 | 0.7760 |
|  | Averages | 0.6608 | 0.7046 | 0.8027 | 0.8044 | **0.8369** | 0.8090 | 0.8135 |
| K562 | AQR | 0.6867 | 0.7060 | 0.7759 | 0.8235 | **0.8495** | 0.8368 | 0.8375 |
|  | BUD13 | 0.6365 | 0.6868 | 0.7867 | 0.8089 | **0.8339** | 0.8022 | 0.8203 |
|  | DDX3X | 0.7768 | 0.7913 | 0.8243 | 0.8285 | **0.8565** | 0.8412 | 0.8388 |
|  | EFTUD2 | 0.6953 | 0.7326 | 0.8360 | 0.8280 | **0.8525** | 0.8424 | 0.8394 |
|  | GRWD1 | 0.6418 | 0.6644 | 0.7384 | 0.7189 | **0.7649** | 0.7431 | 0.7446 |
|  | PRPF8 | 0.6960 | 0.7627 | 0.8480 | 0.8494 | **0.8860** | 0.8596 | 0.8717 |
|  | RPS3 | 0.5979 | 0.6631 | 0.7499 | 0.7102 | **0.7864** | 0.7571 | 0.7592 |
|  | SF3B4 | 0.7648 | 0.8312 | 0.8457 | 0.8782 | **0.9110** | 0.8633 | 0.8831 |
|  | SND1 | 0.6008 | 0.6526 | 0.7523 | 0.7646 | **0.7734** | 0.7347 | 0.7477 |
|  | UPF1 | 0.7101 | 0.6734 | 0.7782 | 0.7609 | **0.8157** | 0.7879 | 0.7860 |
|  | Averages | 0.6807 | 0.7164 | 0.7935 | 0.7971 | **0.8330** | 0.8068 | 0.8128 |

**Table S22** Precision of cross-test on each dataset. 'Without Attention' refers to the performance metrics when the attention mechanism is removed from the diPaRIS model, while 'Without icSHAPE-DS' indicates the metrics obtained by using raw data without incorporating icSHAPE-DS encoding.

| Cell lines | Proteins | Methods | | | | | | |
| --- | --- | --- | --- | --- | --- | --- | --- | --- |
|  |  | DeepBind | DeepRiPe | PrismNet | HDRNet | diPaRIS | | |
|  |  |  |  |  |  |  | without Attention | without icSHAPE-DS |
| HepG2 | AQR | 0.6501 | 0.6921 | 0.7763 | 0.8460 | **0.8687** | 0.8599 | **0.8704** |
|  | BUD13 | 0.5901 | 0.6803 | 0.6732 | 0.7848 | **0.8293** | 0.8083 | **0.8514** |
|  | DDX3X | 0.8100 | 0.8267 | 0.8600 | 0.8460 | **0.8830** | 0.8970 | **0.8894** |
|  | EFTUD2 | 0.6742 | 0.7348 | 0.7774 | 0.8676 | **0.8791** | 0.8729 | 0.8589 |
|  | GRWD1 | 0.6866 | 0.7116 | 0.7955 | 0.7919 | **0.8158** | 0.8208 | 0.8145 |
|  | PRPF8 | 0.7370 | 0.7828 | 0.8502 | 0.8630 | **0.8827** | 0.8679 | 0.8766 |
|  | RPS3 | 0.6114 | 0.6856 | 0.7361 | 0.7965 | 0.8115 | 0.8377 | **0.8277** |
|  | SF3B4 | 0.6803 | 0.7548 | 0.7922 | 0.8888 | **0.9086** | 0.8731 | 0.8806 |
|  | SND1 | 0.6157 | 0.6728 | 0.7648 | 0.7868 | 0.7633 | 0.7842 | **0.8245** |
|  | UPF1 | 0.6572 | 0.7000 | 0.7151 | 0.7582 | 0.8173 | 0.8135 | **0.8272** |
|  | Averages | 0.6713 | 0.7242 | 0.7741 | 0.8230 | 0.8459 | 0.8435 | **0.8521** |
| K562 | AQR | 0.6728 | 0.7576 | 0.6782 | 0.8546 | 0.8668 | 0.8716 | **0.8902** |
|  | BUD13 | 0.6255 | 0.7007 | 0.7885 | 0.8388 | 0.8356 | **0.8494** | 0.8256 |
|  | DDX3X | 0.7970 | 0.7853 | 0.8337 | 0.8259 | 0.8570 | 0.8135 | **0.8745** |
|  | EFTUD2 | 0.7070 | 0.7554 | 0.8152 | 0.8401 | **0.8727** | 0.8483 | 0.8571 |
|  | GRWD1 | 0.6793 | 0.7246 | 0.7655 | 0.8458 | **0.8546** | 0.8258 | 0.8287 |
|  | PRPF8 | 0.6940 | 0.7653 | 0.8184 | 0.8660 | 0.8617 | 0.8590 | **0.8709** |
|  | RPS3 | 0.6041 | 0.6592 | 0.7188 | **0.8128** | 0.7835 | 0.7981 | 0.8048 |
|  | SF3B4 | 0.7235 | 0.8307 | 0.8770 | 0.8929 | **0.9045** | 0.8905 | 0.8812 |
|  | SND1 | 0.6217 | 0.6771 | 0.7368 | 0.7516 | **0.8068** | 0.7937 | 0.8018 |
|  | UPF1 | 0.6423 | 0.7153 | 0.6951 | 0.8197 | 0.8058 | 0.8234 | **0.8383** |
|  | Averages | 0.6767 | 0.7371 | 0.7727 | 0.8348 | 0.8449 | 0.8373 | **0.8473** |

**Table S23** Recall of cross-test on each dataset. 'Without Attention' refers to the performance metrics when the attention mechanism is removed from the diPaRIS model, while 'Without icSHAPE-DS' indicates the metrics obtained by using raw data without incorporating icSHAPE-DS encoding.

| Cell lines | Proteins | Methods | | | | | | |
| --- | --- | --- | --- | --- | --- | --- | --- | --- |
|  |  | DeepBind | DeepRiPe | PrismNet | HDRNet | diPaRIS | | |
|  |  |  |  |  |  |  | without Attention | without icSHAPE-DS |
| HepG2 | AQR | 0.6347 | 0.7202 | **0.9065** | 0.8144 | 0.8665 | 0.8292 | 0.8428 |
|  | BUD13 | 0.6382 | 0.6932 | **0.8641** | 0.7961 | 0.8023 | 0.7799 | 0.7354 |
|  | DDX3X | 0.6990 | 0.6819 | 0.8068 | 0.8286 | **0.8416** | 0.7783 | 0.8100 |
|  | EFTUD2 | 0.6236 | 0.6533 | **0.8886** | 0.7764 | 0.8340 | 0.7907 | 0.8061 |
|  | GRWD1 | 0.7683 | 0.8005 | 0.8529 | 0.8191 | **0.8711** | 0.8140 | 0.8326 |
|  | PRPF8 | 0.7002 | 0.6766 | 0.7680 | 0.7994 | **0.8183** | 0.7683 | 0.7836 |
|  | RPS3 | 0.6333 | 0.7180 | **0.9217** | 0.7973 | 0.8631 | 0.7942 | 0.8188 |
|  | SF3B4 | 0.6232 | 0.6652 | **0.8127** | 0.7057 | 0.7267 | 0.6864 | 0.6749 |
|  | SND1 | 0.5771 | 0.6530 | 0.8068 | 0.7586 | **0.8758** | 0.7897 | 0.7692 |
|  | UPF1 | 0.6597 | 0.6440 | **0.8348** | 0.8161 | 0.8145 | 0.7586 | 0.7307 |
|  | Averages | 0.6557 | 0.6906 | **0.8463** | 0.7912 | 0.8314 | 0.7789 | 0.7804 |
| K562 | AQR | 0.7013 | 0.6613 | **0.9125** | 0.7950 | 0.8329 | 0.8047 | 0.7906 |
|  | BUD13 | 0.6489 | 0.6745 | 0.7949 | 0.7825 | **0.8323** | 0.7600 | 0.8151 |
|  | DDX3X | 0.7576 | 0.7979 | 0.8180 | 0.8339 | **0.8561** | 0.8708 | 0.8058 |
|  | EFTUD2 | 0.6852 | 0.7119 | **0.8584** | 0.8215 | 0.8332 | 0.8366 | 0.8224 |
|  | GRWD1 | 0.6085 | 0.6135 | **0.7185** | 0.6251 | 0.6923 | 0.6754 | 0.6760 |
|  | PRPF8 | 0.6991 | 0.7605 | 0.8865 | 0.8359 | **0.9118** | 0.8602 | 0.8725 |
|  | RPS3 | 0.5980 | 0.6675 | **0.7987** | 0.6308 | 0.7893 | 0.7201 | 0.7185 |
|  | SF3B4 | 0.8113 | 0.8320 | 0.8194 | 0.8642 | **0.9175** | 0.8377 | 0.8851 |
|  | SND1 | 0.5815 | 0.6337 | **0.7694** | 0.7810 | 0.7426 | 0.6839 | 0.7004 |
|  | UPF1 | 0.7941 | 0.6370 | **0.8927** | 0.7137 | 0.8259 | 0.7554 | 0.7399 |
|  | Averages | 0.6886 | 0.6990 | **0.8269** | 0.7684 | 0.8234 | 0.7805 | 0.7826 |

**Table S24** Prediction of IGF2BP1 binding sites in the HepG2 cell line using a model trained on the IGF2BP1-K562 dataset. A total of 1,856 binding sites are predicted, and the number of correctly predicted binding sites is listed.

| DeepBind | DeepRiPe | PrismNet | HDRNet | diPaRIS |
| --- | --- | --- | --- | --- |
| 943 | 1048 | 1396 | 1402 | **1455** |

**Table S25** Prediction of ZNF800 binding sites in the HepG2 and HEK293 cell lines using diPaRIS trained on the ZNF800-K562 dataset.

| Cell lines | HepG2 | HEK293 |
| --- | --- | --- |
| Number of binding sites | 1106 | 89 |
| Number of correctly predicted binding sites | 839 | 64 |

**Table S26** ACC of cross-test on IGF2BPs family (IGF2BP1 and IGF2BP2), DDXs family (DDX24 and DDX3X), and SF3B4.

| Methods | Proteins | | | | |
| --- | --- | --- | --- | --- | --- |
|  |  | IGF2BP1 | IGF2BP2 | DDX24 | DDX3X |
| DeepBind | IGF2BP2 | 0.6108 |  |  |  |
| DeepRiPe |  | 0.6110 |  |  |  |
| PrismNet |  | 0.7125 |  |  |  |
| HDRNet |  | 0.6571 |  |  |  |
| diPaRIS |  | **0.7413** |  |  |  |
| DeepBind | DDX24 | 0.4764 | 0.4671 |  |  |
| DeepRiPe |  | 0.5358 | 0.5456 |  |  |
| PrismNet |  | 0.6341 | 0.6356 |  |  |
| HDRNet |  | 0.5764 | 0.5924 |  |  |
| diPaRIS |  | **0.6561** | **0.6461** |  |  |
| DeepBind | DDX3X | 0.4164 | 0.4701 | 0.5203 |  |
| DeepRiPe |  | 0.4682 | 0.5278 | 0.5835 |  |
| PrismNet |  | **0.5955** | 0.6129 | 0.7326 |  |
| HDRNet |  | 0.5248 | 0.6070 | 0.6335 |  |
| diPaRIS |  | 0.5849 | **0.6201** | **0.7548** |  |
| DeepBind | SF3B4 | 0.5270 | 0.5271 | 0.5326 | 0.4831 |
| DeepRiPe |  | 0.5189 | 0.5131 | 0.5191 | 0.4813 |
| PrismNet |  | 0.6148 | 0.5655 | 0.6645 | 0.6307 |
| HDRNet |  | 0.5231 | 0.5129 | 0.5830 | 0.5280 |
| diPaRIS |  | **0.6818** | **0.5926** | **0.6802** | **0.6328** |

**Table S27** AUC of cross-test on IGF2BPs family (IGF2BP1 and IGF2BP2), DDXs family (DDX24 and DDX3X), and SF3B4.

| Methods | Proteins | | | | |
| --- | --- | --- | --- | --- | --- |
|  |  | IGF2BP1 | IGF2BP2 | DDX24 | DDX3X |
| DeepBind | IGF2BP2 | 0.6600 |  |  |  |
| DeepRiPe |  | 0.6543 |  |  |  |
| PrismNet |  | 0.7802 |  |  |  |
| HDRNet |  | 0.8007 |  |  |  |
| diPaRIS |  | **0.8324** |  |  |  |
| DeepBind | DDX24 | 0.4719 | 0.4509 |  |  |
| DeepRiPe |  | 0.5533 | 0.5775 |  |  |
| PrismNet |  | 0.6873 | 0.7416 |  |  |
| HDRNet |  | 0.6903 | 0.7205 |  |  |
| diPaRIS |  | **0.7171** | **0.7422** |  |  |
| DeepBind | DDX3X | 0.3275 | 0.4479 | 0.5310 |  |
| DeepRiPe |  | 0.3990 | 0.5669 | 0.6445 |  |
| PrismNet |  | 0.6111 | 0.7070 | 0.8450 |  |
| HDRNet |  | 0.6408 | **0.7494** | 0.7947 |  |
| diPaRIS |  | **0.6573** | 0.7319 | **0.8466** |  |
| DeepBind | SF3B4 | 0.5421 | 0.5415 | 0.5517 | 0.4832 |
| DeepRiPe |  | 0.5377 | 0.5250 | 0.5272 | 0.4557 |
| PrismNet |  | 0.6229 | 0.6003 | 0.6929 | 0.6787 |
| HDRNet |  | 0.6272 | 0.5856 | **0.7466** | 0.6409 |
| diPaRIS |  | **0.6274** | **0.6274** | 0.7312 | **0.6781** |

**Table S28** AUPR of cross-test on IGF2BPs family (IGF2BP1 and IGF2BP2), DDXs family (DDX24 and DDX3X), and SF3B4.

| Methods | Proteins | | | | |
| --- | --- | --- | --- | --- | --- |
|  |  | IGF2BP1 | IGF2BP2 | DDX24 | DDX3X |
| DeepBind | IGF2BP2 | 0.7014 |  |  |  |
| DeepRiPe |  | 0.6966 |  |  |  |
| PrismNet |  | 0.7838 |  |  |  |
| HDRNet |  | 0.7631 |  |  |  |
| diPaRIS |  | **0.8108** |  |  |  |
| DeepBind | DDX24 | 0.5552 | 0.5751 |  |  |
| DeepRiPe |  | 0.6177 | 0.6312 |  |  |
| PrismNet |  | 0.7243 | 0.7185 |  |  |
| HDRNet |  | 0.6925 | 0.7037 |  |  |
| diPaRIS |  | **0.7438** | **0.7310** |  |  |
| DeepBind | DDX3X | 0.4677 | 0.5606 | 0.6290 |  |
| DeepRiPe |  | 0.4995 | 0.6030 | 0.6695 |  |
| PrismNet |  | **0.6720** | 0.6988 | 0.8194 |  |
| HDRNet |  | 0.6040 | **0.7116** | 0.7643 |  |
| diPaRIS |  | 0.6619 | 0.7076 | **0.8243** |  |
| DeepBind | SF3B4 | 0.6109 | 0.6283 | 0.6347 | 0.5630 |
| DeepRiPe |  | 0.5871 | 0.5834 | 0.5945 | 0.5266 |
| PrismNet |  | **0.7014** | 0.6484 | 0.7473 | 0.7253 |
| HDRNet |  | 0.6149 | 0.5876 | 0.7436 | 0.6389 |
| diPaRIS |  | 0.6336 | **0.6718** | **0.7676** | **0.7254** |

**Table S29** F1-score of cross-test on IGF2BPs family (IGF2BP1 and IGF2BP2), DDXs family (DDX24 and DDX3X), and SF3B4.

| Methods | Proteins | | | | |
| --- | --- | --- | --- | --- | --- |
|  |  | IGF2BP1 | IGF2BP2 | DDX24 | DDX3X |
| DeepBind | IGF2BP2 | 0.5885 |  |  |  |
| DeepRiPe |  | 0.5642 |  |  |  |
| PrismNet |  | 0.7034 |  |  |  |
| HDRNet |  | 0.5400 |  |  |  |
| diPaRIS |  | **0.7118** |  |  |  |
| DeepBind | DDX24 | 0.3572 | 0.4157 |  |  |
| DeepRiPe |  | 0.4376 | 0.4641 |  |  |
| PrismNet |  | 0.5488 | **0.5861** |  |  |
| HDRNet |  | 0.3354 | 0.3889 |  |  |
| diPaRIS |  | **0.5690** | 0.5554 |  |  |
| DeepBind | DDX3X | 0.2497 | 0.3806 | 0.4937 |  |
| DeepRiPe |  | 0.2416 | 0.3998 | 0.5111 |  |
| PrismNet |  | **0.5000** | **0.5525** | 0.6750 |  |
| HDRNet |  | 0.1835 | 0.4055 | 0.4603 |  |
| diPaRIS |  | 0.4224 | 0.5043 | **0.7250** |  |
| DeepBind | SF3B4 | 0.4335 | 0.4871 | 0.4952 | 0.3728 |
| DeepRiPe |  | 0.3577 | 0.3687 | 0.3886 | 0.2728 |
| PrismNet |  | 0.5031 | **0.4590** | **0.6110** | 0.5056 |
| HDRNet |  | 0.1576 | 0.1601 | 0.3114 | 0.1594 |
| diPaRIS |  | **0.5808** | 0.4217 | 0.6082 | **0.5108** |

**Table S30** Precision of cross-test on IGF2BPs family (IGF2BP1 and IGF2BP2), DDXs family (DDX24 and DDX3X), and SF3B4.

| Methods | Proteins | | | | |
| --- | --- | --- | --- | --- | --- |
|  |  | IGF2BP1 | IGF2BP2 | DDX24 | DDX3X |
| DeepBind | IGF2BP2 | 0.6169 |  |  |  |
| DeepRiPe |  | 0.6413 |  |  |  |
| PrismNet |  | 0.7261 |  |  |  |
| HDRNet |  | **0.8244** |  |  |  |
| diPaRIS |  | 0.7990 |  |  |  |
| DeepBind | DDX24 | 0.4574 | 0.4598 |  |  |
| DeepRiPe |  | 0.5534 | 0.5653 |  |  |
| PrismNet |  | 0.7247 | 0.6701 |  |  |
| HDRNet |  | **0.7780** | **0.7734** |  |  |
| diPaRIS |  | 0.7597 | 0.7363 |  |  |
| DeepBind | DDX3X | 0.3327 | 0.4581 | 0.5238 |  |
| DeepRiPe |  | 0.4112 | 0.5481 | 0.6198 |  |
| PrismNet |  | 0.6221 | 0.6553 | 0.8610 |  |
| HDRNet |  | 0.6544 | **0.7759** | **0.8723** |  |
| diPaRIS |  | **0.6892** | 0.7192 | 0.8254 |  |
| DeepBind | SF3B4 | 0.5398 | 0.5318 | 0.5366 | 0.4702 |
| DeepRiPe |  | 0.5391 | 0.5236 | 0.5346 | 0.4559 |
| PrismNet |  | **0.7070** | 0.6082 | 0.7306 | **0.7592** |
| HDRNet |  | 0.6845 | 0.6271 | **0.8929** | 0.7330 |
| diPaRIS |  | 0.5736 | **0.6808** | 0.7862 | 0.7564 |

**Table S31** Recall of cross-test on IGF2BPs family (IGF2BP1 and IGF2BP2), DDXs family (DDX24 and DDX3X), and SF3B4.

| Methods | Proteins | | | | |
| --- | --- | --- | --- | --- | --- |
|  |  | IGF2BP1 | IGF2BP2 | DDX24 | DDX3X |
| DeepBind | IGF2BP2 | 0.5717 |  |  |  |
| DeepRiPe |  | 0.5036 |  |  |  |
| PrismNet |  | **0.6832** |  |  |  |
| HDRNet |  | 0.4033 |  |  |  |
| diPaRIS |  | 0.6452 |  |  |  |
| DeepBind | DDX24 | 0.3059 | 0.3808 |  |  |
| DeepRiPe |  | 0.3637 | 0.3941 |  |  |
| PrismNet |  | 0.4475 | **0.5336** |  |  |
| HDRNet |  | 0.2138 | 0.2682 |  |  |
| diPaRIS |  | **0.4560** | 0.4513 |  |  |
| DeepBind | DDX3X | 0.2055 | 0.3261 | 0.4684 |  |
| DeepRiPe |  | 0.1755 | 0.3159 | 0.4384 |  |
| PrismNet |  | **0.4436** | 0.4844 | **0.5553** |  |
| HDRNet |  | 0.1069 | 0.2945 | 0.3126 |  |
| diPaRIS |  | 0.3052 | **0.6667** | 0.4901 |  |
| DeepBind | SF3B4 | 0.3638 | **0.4497** | 0.4655 | 0.3114 |
| DeepRiPe |  | 0.2701 | 0.2863 | 0.3088 | 0.1947 |
| PrismNet |  | 0.3917 | 0.3775 | **0.5281** | 0.3830 |
| HDRNet |  | 0.0906 | 0.0960 | 0.1886 | 0.0895 |
| diPaRIS |  | **0.4287** | 0.3254 | 0.4980 | **0.3888** |

**Table S32** Prediction of IGF2BP1 binding sites in the HepG2 cell line using a model trained on the IGF2BP1-K562 dataset. A total of 1,856 binding sites are predicted, and the number of correctly predicted binding sites is listed.

| Training dataset | Methods | | | | |
| --- | --- | --- | --- | --- | --- |
|  | DeepBind | DeepRiPe | PrismNet | HDRNet | diPaRIS |
| IGF2BP1-K562 | 778 | 882 | **1222** | 1179 | 1210 |
| IGF2BP2-K562 | **1270** | 1103 | 1175 | 1151 | 1217 |
| Sum | 2048 | 1985 | 2399 | 2330 | **2427** |

**Table S33** Statistics of protein binding sites with significant changes before and after editing^[4]^.

| Proteins | Chromosomes | Positions | Strands | Predicted scores | |
| --- | --- | --- | --- | --- | --- |
|  |  |  |  | Before editing | After editing |
| AQR | Chr3 | 38,416,056 | + | 0.0724 | 0.8227 |
| AQR | Chr4 | 4,281,057 | - | 0.2942 | 0.6196 |
| AQR | Chr13 | 27,829,728 | + | 0.1694 | 0.8569 |
| BUD13 | Chr3 | 141,638,149 | + | 0.1471 | 0.8577 |
| BUD13 | Chr7 | 157,025,069 | + | 0.2287 | 0.7777 |
| BUD13 | Chr8 | 30,921,263 | + | 0.2352 | 0.8506 |
| BUD13 | Chr9 | 134,082,063 | + | 0.1899 | 0.6642 |
| BUD13 | Chr20 | 29,633,900 | + | 0.2940 | 0.7772 |
| EFTUD2 | Chr1 | 207,933,313 | + | 0.2729 | 0.6213 |
| FAM120A | Chr14 | 74,526,945 | - | 0.3453 | 0.8799 |
| FMR1 | Chr5 | 56,535,963 | + | 0.0082 | 0.6236 |
| GRWD1 | Chr19 | 34,883,447 | + | 0.6767 | 0.3053 |
| IGF2BP2 | Chr2 | 37,326,527 | + | 0.0598 | 0.6348 |
| IGF2BP2 | Chr6 | 122,765,207 | - | 0.6758 | 0.1306 |
| IGF2BP2 | Chr17 | 38,974,612 | - | 0.2165 | 0.9742 |
| IGF2BP2 | Chr18 | 47,918,787 | + | 0.0197 | 0.8559 |
| IGF2BP2 | Chr19 | 58,373,217 | - | 0.0815 | 0.8428 |
| LIN28B | Chr8 | 11,683,622 | - | 0.9835 | 0.3033 |
| LIN28B | Chr10 | 32,192,253 | - | 0.5722 | 0.4733 |
| METAP2 | Chr3 | 10,086,903 | + | 0.4966 | 0.6789 |
| PUM2 | Chr5 | 154,196,157 | + | 0.7703 | 0.0197 |
| RBM15 | Chr18 | 204,652 | + | 0.4459 | 0.5355 |
| RPS3 | Chr1 | 149,859,159 | + | 0.5415 | 0.0530 |
| RPS3 | Chr19 | 34,883,447 | + | 0.4724 | 0.9126 |
| SF3B4 | Chr22 | 17,082,952 | + | 0.7478 | 0.0027 |
| SND1 | Chr7 | 139,051,749 | + | 0.4156 | 0.7478 |
| SND1 | Chr18 | 9,110,528 | + | 0.2346 | 0.7654 |
| SND1 | Chr18 | 9,110,530 | + | 0.2118 | 0.5450 |
| UPF1 | Chr1 | 225,976,051 | + | 0.4399 | 0.8433 |
| UPF1 | Chr3 | 45,722,047 | + | 0.6064 | 0.3703 |
| UPF1 | Chr3 | 48,787,909 | - | 0.8675 | 0.4686 |
| UPF1 | Chr7 | 44,841,489 | + | 0.7241 | 0.0525 |
| UPF1 | Chr7 | 158,530,894 | - | 0.4818 | 0.7510 |
| UPF1 | Chr9 | 37,503,346 | + | 0.1624 | 0.6164 |
| UPF1 | Chr12 | 69,237,007 | + | 0.0843 | 0.5157 |
| UPF1 | Chr12 | 69,237,043 | + | 0.4838 | 0.9983 |
| UPF1 | Chr19 | 1,590,602 | - | 0.3390 | 0.6173 |
| UPF1 | Chr19 | 11,256,725 | - | 0.7016 | 0.1050 |
| UPF1 | Chr19 | 11,560,427 | + | 0.3084 | 0.9467 |
| UPF1 | Chr22 | 29,902,996 | - | 0.2408 | 0.8046 |
| UPF1 | Chr22 | 40,357,027 | + | 0.1582 | 0.5658 |
| UPF1 | ChrX | 100,265,385 | - | 0.5791 | 0.0839 |
| YBX3 | Chr1 | 225,974,614 | + | 0.4409 | 0.8405 |
| YBX3 | Chr5 | 68,400,178 | + | 0.7264 | 0.4564 |
| YBX3 | Chr16 | 29,679,052 | + | 0.8434 | 0.2853 |
| ZNF622 | Chr21 | 34,821,105 | - | 0.4969 | 0.7423 |

**Table S34** List of parameter settings for diPaRIS.

| Parameter | Description | Default value |
| --- | --- | --- |
| F_alignment_ | Number of filters for CNNs in the feature alignment module. | 64 |
| K_sequence_ | Kernel size for CNNs processing sequence features in the feature alignment module. | (6, 4) |
| K_structure_ | Kernel size for CNNs processing structure features in the feature alignment module. | (10, 7) |
| U | Number of units for Bi-LSTMs in the feature alignment module. | 32 |
| F_downsample_ | Number of filters for (1, 1) CNNs in the downsampling module. | [32, 64, 128] |
| F_downsample_ | Number of filters for (3, 3) CNNs in the downsampling module. | [8, 16, 32] |
| P_downsample_ | Pool size for MaxPoolings in the downsampling module. | (1, 2) |
| R_dropout_ | Rate for Dropout in diPaRIS. | 0.3 |
| R_regularizert_ | Rate for Regularizers in diPaRIS | 0.001 |
| H | Number of heads for the MultiHeadAttention in the jump connection module. | [1, 2, 4] |
| F_bottleneck_ | Number of filters for (1, 1) CNNs in the bottleneck module. | 256 |
| F_bottleneck_ | Number of filters for (3, 3) CNNs in the bottleneck module. | 64 |
| K_transpose_ | Kernel size for transpose CNNs in the upsampling module. | 3 |
| S | Strides for transpose CNNs in the upsampling module. | 2 |
| F_upsample_ | Number of filters for (1, 1) CNNs in the upsampling module. | [128, 64, 32] |
| F_upsample_ | Number of filters for (3, 3) CNNs in the upsampling module. | [32, 16, 8] |
| P_classify_ | Pool size for AveragePoolings in the binding site prediction module. | [201, 40, 8] |
| U_classify_ | Unit for MLP in the binding site prediction module. | [16, 8, 4, 2] |

## Supplementary References

[1] a) P. D. Thomas, D. Ebert, A. Muruganujan, T. Mushayahama, L. P. Albou, H. Mi, *Protein Sci.* **2022**, *31*, 8; b) C. Gene Ontology, S. A. Aleksander, J. Balhoff, S. Carbon, J. M. Cherry, H. J. Drabkin, D. Ebert, M. Feuermann, P. Gaudet, N. L. Harris, D. P. Hill, R. Lee, H. Mi, S. Moxon, C. J. Mungall, A. Muruganugan, T. Mushayahama, P. W. Sternberg, P. D. Thomas, K. Van Auken, J. Ramsey, D. A. Siegele, R. L. Chisholm, P. Fey, M. C. Aspromonte, M. V. Nugnes, F. Quaglia, S. Tosatto, M. Giglio, S. Nadendla, G. Antonazzo, H. Attrill, G. Dos Santos, S. Marygold, V. Strelets, C. J. Tabone, J. Thurmond, P. Zhou, S. H. Ahmed, P. Asanitthong, D. Luna Buitrago, M. N. Erdol, M. C. Gage, M. Ali Kadhum, K. Y. C. Li, M. Long, A. Michalak, A. Pesala, A. Pritazahra, S. C. C. Saverimuttu, R. Su, K. E. Thurlow, R. C. Lovering, C. Logie, S. Oliferenko, J. Blake, K. Christie, L. Corbani, M. E. Dolan, H. J. Drabkin, D. P. Hill, L. Ni, D. Sitnikov, C. Smith, A. Cuzick, J. Seager, L. Cooper, J. Elser, P. Jaiswal, P. Gupta, P. Jaiswal, S. Naithani, M. Lera-Ramirez, K. Rutherford, V. Wood, J. L. De Pons, M. R. Dwinell, G. T. Hayman, M. L. Kaldunski, A. E. Kwitek, S. J. F. Laulederkind, M. A. Tutaj, M. Vedi, S. J. Wang, P. D'Eustachio, L. Aimo, K. Axelsen, A. Bridge, N. Hyka-Nouspikel, A. Morgat, S. A. Aleksander, J. M. Cherry, S. R. Engel, K. Karra, S. R. Miyasato, R. S. Nash, M. S. Skrzypek, S. Weng, E. D. Wong, E. Bakker, T. Z. Berardini, L. Reiser, A. Auchincloss, K. Axelsen, G. Argoud-Puy, M. C. Blatter, E. Boutet, L. Breuza, A. Bridge, C. Casals-Casas, E. Coudert, A. Estreicher, M. Livia Famiglietti, M. Feuermann, A. Gos, N. Gruaz-Gumowski, C. Hulo, N. Hyka-Nouspikel, F. Jungo, P. Le Mercier, D. Lieberherr, P. Masson, A. Morgat, I. Pedruzzi, L. Pourcel, S. Poux, C. Rivoire, S. Sundaram, A. Bateman, E. Bowler-Barnett, A. J. H. Bye, P. Denny, A. Ignatchenko, R. Ishtiaq, A. Lock, Y. Lussi, M. Magrane, M. J. Martin, S. Orchard, P. Raposo, E. Speretta, N. Tyagi, K. Warner, R. Zaru, A. D. Diehl, R. Lee, J. Chan, S. Diamantakis, D. Raciti, M. Zarowiecki, M. Fisher, C. James-Zorn, V. Ponferrada, A. Zorn, S. Ramachandran, L. Ruzicka, M. Westerfield, *Genetics.* **2023**, *224*, iyad031.

[2] N. A. O'Leary, M. W. Wright, J. R. Brister, S. Ciufo, D. Haddad, R. McVeigh, B. Rajput, B. Robbertse, B. Smith-White, D. Ako-Adjei, A. Astashyn, A. Badretdin, Y. Bao, O. Blinkova, V. Brover, V. Chetvernin, J. Choi, E. Cox, O. Ermolaeva, C. M. Farrell, T. Goldfarb, T. Gupta, D. Haft, E. Hatcher, W. Hlavina, V. S. Joardar, V. K. Kodali, W. Li, D. Maglott, P. Masterson, K. M. McGarvey, M. R. Murphy, K. O'Neill, S. Pujar, S. H. Rangwala, D. Rausch, L. D. Riddick, C. Schoch, A. Shkeda, S. S. Storz, H. Sun, F. Thibaud-Nissen, I. Tolstoy, R. E. Tully, A. R. Vatsan, C. Wallin, D. Webb, W. Wu, M. J. Landrum, A. Kimchi, T. Tatusova, M. DiCuccio, P. Kitts, T. D. Murphy, K. D. Pruitt, *Nucleic Acids Res.* **2016**, *44*, D733.

[3] J. Mistry, S. Chuguransky, L. Williams, M. Qureshi, G. A. Salazar, E. L. L. Sonnhammer, S. C. E. Tosatto, L. Paladin, S. Raj, L. J. Richardson, R. D. Finn, A. Bateman, *Nucleic Acids Res.* **2021**, *49*, D412.

[4] X. Hu, Q. Zou, L. Yao, X. Yang, *Genome Biol.* **2022**, *23*, 169.
